# Multi-UAV Cooperative Path Planning Using a Behavior-Adaptive Aquila Optimizer Under Multiple Constraints

**DOI:** 10.3390/biomimetics11030166

**Published:** 2026-03-01

**Authors:** Xiaojie Tang, Chengfen Jia, Pengju Qu, Qian Zhang, Pan Zhang

**Affiliations:** 1 School of Intelligent Manufacturing, Sichuan University Jinjiang College, Meishan 620860, China; jiachengfen@scujj.edu.cn (C.J.); zhangqian@scujj.edu.cn (Q.Z.); zhangpan@scujj.edu.cn (P.Z.); 2 Key Laboratory of Advanced Manufacturing Technology of the Ministry of Education, Guizhou University, Guiyang 550025, China; 20140497@git.edu.cn; 3 Engineering Training Center, Guizhou Institute of Technology, Guiyang 550025, China

**Keywords:** aquila optimizer, multi-UAV cooperative path planning, metaheuristic, behavior-adaptive selection

## Abstract

Addressing the challenges of high dimensionality, strong nonlinearity, and multiple constraints in multi-UAV cooperative path planning, this paper proposes a Behavior-Adaptive Aquila Optimizer (EAO) achieved by enhancing Aquila Optimizer (AO). EAO constructs a multi-strategy cooperative framework that integrates a periodic diversity maintenance mechanism, a diversity-based dynamic neighborhood guidance mechanism, a narrowed exploitation behavior based on neighborhood differential evolution, and a search-state-aware adaptive behavior selection mechanism. Through dynamic behavior adjustment during the search process, the proposed algorithm improves search performance and stability. To validate its effectiveness, EAO was systematically evaluated on the CEC2017 and CEC2020 benchmark suites and compared with the original AO and 13 representative high-performance optimization algorithms. Parameter sensitivity analysis, an ablation study, and an exploration–exploitation experiment were also conducted. The results show that EAO achieves the best overall performance ranking. Furthermore, EAO was applied to multi-UAV cooperative path-planning simulations in complex environments that considered UAV dynamic constraints. Comparative experiments with five competitive algorithms demonstrate that EAO achieves superior performance in terms of path-planning fitness, number of effective trajectories, and runtime. Compared with AO, EAO improves the average fitness by 80.42%, 81.25%, 81.34%, and 84.84% across different map environments, confirming its feasibility and effectiveness for multi-UAV cooperative path planning.

## 1. Introduction

In recent years, unmanned aerial vehicles have been widely applied in military reconnaissance, disaster relief, logistics distribution, and complex environment inspection due to their advantages of high mobility, flexible deployment, and relatively low cost. As task scales expand and operational environments become more complex, single-UAV systems have gradually exposed deficiencies in efficiency and robustness when performing multi-target, high-risk tasks [1,2]. Multi-UAV cooperative task execution has thus become an important research direction in the field.

Path planning is a core problem in multi-UAV systems that affects mission success rate and system safety. Effective path planning must not only ensure that UAVs can avoid threat zones and no-fly areas in complex environments, but also comprehensively consider flight energy consumption, task coordination efficiency, and overall system stability. Consequently, multi-UAV path planning is typically characterized as a complex optimization problem with high dimensionality, strong nonlinearity, and multiple constraints [3], placing high demands on solution algorithms.

Traditional deterministic methods for multi-UAV path planning, such as graph-search or mathematical programming approaches, including Dijkstra [4], A-star [5], D* Lite [6] algorithms, and mixed-integer linear programming [7], generally rely on precise modeling and problem-structure assumptions. When dealing with high-dimensional continuous spaces and complex constraints, these methods often suffer from high computational complexity, poor scalability, and susceptibility to local optima. In contrast, swarm intelligence optimization algorithms have gradually become a significant research direction in UAV path planning due to their strong global search capabilities and lower dependency on problem modeling [8].

In recent years, particle swarm optimization (PSO, 1995) [9], differential evolution (DE, 1995) [10], whale optimization algorithm (WOA, 2016) [11], moth–flame optimization (MFO, 2015) [12], and various emerging bio-inspired swarm intelligence algorithms, such as golden jackal optimization (GJO, 2024) [13], dung beetle optimizer (DBO, 2023) [14], and secretary bird optimization algorithm (SBOA, 2024) [15], have been widely applied to multi-UAV cooperative path-planning problems. Qi et al. [16] combined the artificial bee colony algorithm with particle swarm optimization and proposed an improved bee-foraging learning particle swarm optimization algorithm (IBFLPSO). The algorithm was applied to multi-UAV path-planning problems considering multiple energy consumption factors. Zheng et al. [17] proposed a differential evolution-based multi-UAV cooperative coverage algorithm (DECSMU) to address coverage tasks in different regions. Experimental results showed that DECSMU achieved high coverage rates and low energy consumption under collision avoidance constraints. Yu et al. [18] applied a whale optimization algorithm enhanced by simulated annealing (SA-WOA) to multi-UAV cooperative atmospheric sensing problems. The experimental results demonstrated that the proposed method exhibited good planning performance. Karthik et al. [19] proposed a hybrid golden jackal and moth–flame optimization algorithm (HGJMFOA) for coverage path planning, which was used to generate optimal paths supporting efficient and complete multi-UAV coverage. Lou et al. [20] proposed a novel multi-UAV three-dimensional terrain cooperative trajectory planning method based on a cuckoo search golden jackal optimization algorithm (CS-GJO). The simulation results verified that CS-GJO achieved better stability, higher optimization accuracy, and faster convergence. Yang et al. [21] proposed a landmark operator-based dung beetle optimizer (LODBO) by integrating multiple improvement strategies, and extensive simulations showed that LODBO could find optimal paths within shorter time. Zheng et al. [22] developed an improved secretary bird optimization algorithm incorporating weighted multidirectional dynamic learning and adaptive strategy selection mechanisms. The simulation results indicated that the proposed ASHSBOA generated lower-cost flight paths with more stable convergence behavior. Although these methods have improved path-planning performance to some extent, they still suffer from insufficient search efficiency, unstable solution quality, and premature convergence when dealing with high-dimensional search spaces, complex threat environments, and multi-UAV cooperative constraints.

Aquila Optimizer (AO) [23] is a novel bio-inspired swarm intelligence optimization algorithm inspired by the diverse flight and attack behaviors of the Aquila during hunting. By simulating different behavior strategies at various search stages, AO switches between exploration and exploitation, resulting in a clear and intuitive search mechanism. The existing studies have shown that AO exhibits good global search capability on various continuous optimization benchmark functions. However, as problem dimensionality and complexity increase, the original AO still has several limitations [24,25]. These include: (1) rapid loss of population diversity during iterations, which easily leads to premature convergence; (2) a relatively fixed behavior selection mechanism that cannot adapt dynamically to the search state; and (3) insufficient global search ability and stability when addressing high-dimensional and complex optimization problems.

To address these issues, many researchers have proposed improvements to the AO algorithm. For multi-area power system networks, Al-Majidi et al. [26] designed a hybrid Aquila Optimizer–sine cosine algorithm (HSCAO) to solve the sensitivity problem of PID-AGC parameters. Bai et al. [27] developed an improved Aquila Optimizer (MIAO) by introducing phasor operators and other strategies and applied it to hyperparameter optimization of long short-term memory (LSTM) networks. This led to the construction of an MIAO-LSTM model for monthly railway freight volume prediction. Wang et al. [28] applied an optimal scheduling strategy based on a differential mutation Aquila Optimizer integrated within a multi-objective optimization model considering total cost and greenhouse gas (GHG) emission constraints. To address the parameter identification of proton exchange membrane fuel cells (PEMFCs), Singla et al. [29] proposed an improved AO algorithm incorporating novel mutation strategies, referred to as AOAAO. The experimental results demonstrated its high accuracy, robustness, and time efficiency in real-time fuel cell modeling. Zhang et al. [30] combined opposition-based learning with a local escape operator to propose an improved Aquila Optimizer (LEOAO), achieving better flight paths for UAV path planning in three-dimensional environments. To meet the requirements of multi-task trajectory planning for multiple UAVs in three-dimensional agriculture, Liu et al. [31] proposed an interference-enhanced portfolio Aquila Optimizer (IEP-AO). This method improves trajectory search capability in complex operational spaces and large-scale task scenarios, enabling UAVs to escape local optimal trajectories more effectively.

However, existing studies on improving Aquila Optimizer still lack a systematic investigation into the adaptive regulation of its behavior selection mechanism. To address this gap, this paper focuses on multi-UAV cooperative path planning and conducts an in-depth analysis and improvement of AO. An enhanced behavior-adaptive Aquila Optimizer (EAO) is proposed and applied to multi-UAV cooperative path planning in complex environments.

The main research work of this paper can be summarized as follows:

- A multi-strategy cooperative behavior-adaptive Aquila Optimizer is proposed. By incorporating a periodic diversity maintenance mechanism, a diversity-based dynamic neighborhood guidance mechanism, a narrowed exploitation behavior based on neighborhood differential evolution, and a search-state-aware adaptive behavior selection mechanism, agent behaviors can be adaptively selected according to the current search state. This design effectively enhances the global search capability and convergence stability of the algorithm in complex search spaces.
- Through parameter sensitivity experiments, reasonable parameter configurations for EAO are determined, and ablation experiments systematically verify the effectiveness of each improvement strategy. Further exploration–exploitation capability analysis experiments evaluate the behavioral evolution characteristics of the algorithm during the search process. Additionally, based on the CEC2017 and CEC2020 standard benchmark suites, the global optimization performance of EAO on low-, medium-, and high-dimensional problems is systematically tested. The experimental results demonstrate that EAO exhibits significant overall advantages compared to 14 other representative competitive algorithms.
- A multi-UAV cooperative path-planning model is constructed, comprehensively considering threat zones, no-fly zones, and UAV dynamic constraints to improve the feasibility of the algorithm in practical application scenarios. Based on this, four simulation map environments ranging from simple to complex are designed, and multi-UAV cooperative path-planning simulation experiments are conducted to systematically verify the proposed algorithm’s performance in path quality, result stability, and cooperative efficiency.

The structure of this paper is arranged as follows: Section 1 introduces the research background, related research status, and the main research content of this paper. Section 2 presents the mathematical modeling of the multi-UAV path-planning problem. Section 3 explains the principles of the original AO algorithm. Section 4 elaborates on the design philosophy and implementation process of the improved Aquila Optimizer. Section 5 presents an experimental analysis of parameter sensitivity for the EAO algorithm. Section 6 validates the optimization performance of the algorithm based on the CEC2017 and CEC2020 benchmark suites. Section 7 conducts simulation experiments for multi-UAV cooperative path planning and analyzes the results. Section 8 summarizes the entire work.

## 2. Multi-UAV Cooperative Path-Planning Modeling

This section discusses the modeling of multi-UAV cooperative path planning [32,33,34], including decision variables, objective functions, and cost function components.

### 2.1. Decision Variables

The multi-UAV path is composed of $N_{uav}$ UAVs. The trajectory of the ith UAV is represented by $n_{i}$ waypoints. For planning convenience, each waypoint is described using spherical coordinates. Its decision variable x is defined as follows:

(1) $$\;x=\left[\begin{array}{c} \begin{array}{c} r_{1,1},r_{1,2},\cdots ,r_{1,n_{1}},\theta_{1,1},\theta_{1,2},\cdots ,\theta_{1,n_{1}},\varphi_{1,1},\varphi_{1,2},\cdots ,\varphi_{1,n_{1}},\\ r_{2,1},r_{2,2},\cdots ,r_{2,n_{2}},\theta_{2,1},\theta_{2,2},\cdots ,\theta_{1,n_{1}},\varphi_{2,1},\varphi_{2,2},\cdots ,\varphi_{2,n_{2}}, \end{array}\\ \vdots \\ r_{N_{uav},1},r_{N_{uav},2},\cdots ,r_{N_{uav},n_{uav}},\theta_{N_{uav},1},\theta_{N_{uav},2},\cdots ,\theta_{N_{uav},N_{uav}},\varphi_{N_{uav},1},\varphi_{N_{uav},2},\cdots ,\varphi_{N_{uav},n_{uav}},\\ v_{1},v_{2},\cdots ,v_{N_{uav}} \end{array}\right]$$

where $r_{i,j}$ denotes the radial distance of the jth waypoint of the ith UAV; $\theta_{i,j}$ represents the pitch angle of the jth waypoint of the ith UAV, used for vertical obstacle avoidance; $\varphi_{i,j}$ is the azimuth angle of the jth waypoint of the ith UAV, employed for horizontal path planning; and $v_{i}$ indicates the number of waypoints for the ith UAV.

**Figure 1:**
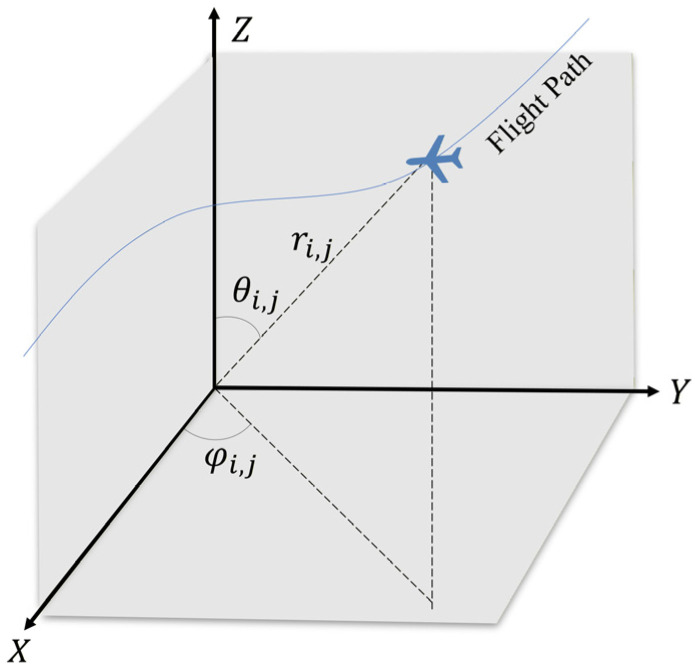
The principle of the decision variable space.

The principle of the decision variable space is illustrated in Figure 1. By applying the transformation function from spherical to Cartesian coordinates, the actual sequence of UAV positions in three-dimensional space can be obtained:

(2) $$\left\{\begin{array}{c} x_{i,j}=x_{i,j-1}+r_{i,j}\cdot cos\theta_{i,j}\cdot sin\varphi_{i,j}\\ y_{i,j}=y_{i,j-1}+r_{i,j}\cdot cos\theta_{i,j}\cdot cos\varphi_{i,j}\\ z_{i,j}=z_{i,j-1}+r_{i,j}\cdot sin\theta_{i,j}\;\;\;\;\;\;\;\;\;\;\;\;\;\;\;\;\;\; \end{array}\right.$$

### 2.2. Objective Function

The objective of the path-planning problem is to find an optimal solution that minimizes the total objective function value. The objective function $F_{T}$ adopts a two-tier penalty design. The weighted cost function $F_{W}$ provides smooth guidance within the feasible region, while the additional penalty term $F_{P}$ strictly enforces critical safety constraints and guarantees feasibility. Their formulas are defined as follows:

(3) $$\;F_{T}=F_{W}+F_{P}$$

The weighted cost function is expressed as a linear combination of multiple sub-cost functions:

(4) $$\;F_{W}=\omega_{1}\cdot f_{1}+\omega_{2}\cdot f_{2}+\omega_{3}\cdot f_{3}+\omega_{4}\cdot f_{4}+\omega_{5}\cdot f_{5}+\omega_{6}\cdot f_{6}+\omega_{7}\cdot f_{7}+\omega_{8}\cdot f_{8}$$

where $f_{1}$ to $f_{8}$ represent the path length cost, altitude cost, threat cost, no-fly zone constraint cost, time coordination cost, collision avoidance cost, angle constraint cost, and trajectory segment constraint cost, respectively. The corresponding weight coefficients are denoted as $\omega_{1}$ to $\omega_{8}$.

To strictly enforce constraints related to threat regions, no-fly zones, and terrain safety, an additional penalty term is imposed beyond the weighted cost function. The penalty term is formulated as follows:

(5) $$F_{P}=F_{M}+100\cdot \left(N_{rc}+N_{ac}\right)+10\cdot f_{7}+200\cdot N_{tc}$$

(6) $$F_{M}=\left\{\begin{array}{cc} 0, & If\;no\;violation\;occurs\;\\ F_{2}\cdot \left(1+2\cdot N_{T}\right)+1000, & otherwise \end{array}\right.$$

(7) $$\;N_{T}=N_{rc}+N_{ac}+N_{tc}+N_{nfz}$$

where $N_{rc}$ denotes the number of penetrations into the core area of the radar threat zone; $N_{ac}$ denotes the number of penetrations into the core area of the artillery threat zone; $N_{tc}$ denotes the number of terrain collisions; $N_{nfz}$ denotes the number of violations in the no-fly zone.

### 2.3. Cost Function Components

#### 2.3.1. Flight Path Cost

The flight path cost function $f_{1}$ aims to generate the shortest flight path to minimize mission duration and energy consumption. It is defined as

(8) $$\;f_{1}=\sum_{i=1}^{N_{uav}}\frac{L_{i}}{L_{max,i}}$$

(9) $$L_{i}=\sum_{j=1}^{n_{i}}\left\|P_{i,j+1}-P_{i,j}\right\|$$

(10) $$\;L_{max,i}=5\cdot \left\|P_{i,n_{i}}-P_{i,1}\right\|$$

where $L_{i}$ the total path length of the ith UAV; $L_{max,i}$ the maximum allowable path length for the ith UAV; and $P_{i,j}$ denotes the Cartesian coordinates of the jth waypoint of the ith UAV.

#### 2.3.2. Altitude Cost

The altitude cost $f_{2}$ ensures that UAVs operate within a safe altitude range to avoid terrain collisions and comply with airspace regulations. It is defined as

(11) $$\;f_{2}=\sum_{i=1}^{N_{uav}}\sum_{k=1}^{n_{i}}{(f}_{col}(i,k)+f_{bo}(i,k))$$

(12) $$f_{col}\left(i,k\right)=\left\{\begin{array}{cc} 1000, & if\;c_{i,k}\leq 10\\ 100, & if\;10<c_{i,k}\leq 20\\ 20, & if\;20<c_{i,k}\leq 30\\ 0, & otherwise \end{array}\right.$$

(13) $$f_{bo\left(i,\;k\right)}=\left\{\begin{array}{cc} 10, & if\;z_{i,k}<H_{min}\;or\;z_{i,k}>H_{max}\\ 0, & otherwise \end{array}\right.$$

where $f_{col}\left(i,k\right)$ denotes the terrain collision penalty, $f_{bo}(i,k)$ represents the altitude boundary violation penalty, and $c_{i,k}$ indicates the ground clearance (altitude above terrain). $H_{min}$ and $H_{max}$ denote the lower and upper bounds of the allowable flight altitude, respectively.

#### 2.3.3. Threat Cost

The threat cost $f_{3}$ forces UAVs to avoid radar and artillery threat zones, which are modeled as spheres in three-dimensional space. The threat cost includes an inverse-distance penalty and a fixed-level penalty. The formula is as follows:

(14) $$f_{3}=\sum_{i=1}^{N_{uav}}\left(\sum_{j=1}^{N_{r}}f_{r}\left(i,j\right)+\sum_{j=1}^{N_{a}}f_{a}\left(i,j\right)\right)$$

(15) $$f_{r}\left(i,j\right)=\left\{\begin{array}{cc} \frac{1}{d_{ij}^{2}}+500, & if\;d_{ij}<0.3R_{r,j}\\ \frac{1}{d_{ij}^{2}}+100, & if\;0.3R_{r,j}\leq d_{ij}<0.6R_{r,j}\\ \frac{1}{d_{ij}^{2}}+20,\; & if\;{0.6R_{r,j}\leq d}_{ij}<R_{r,j}\\ 0, & otherwise \end{array}\right.$$

(16) $$f_{a}\left(i,j\right)=\left\{\begin{array}{cc} \frac{R_{a,j}^{2}}{d_{ij}^{2}+1}+500, & \;if\;d_{ij}<0.3R_{r,j}\\ \frac{R_{a,j}^{2}}{d_{ij}^{2}+1}+100, & if\;{0.3R_{r,j}\leq d}_{ij}<0.6R_{r,j}\\ \frac{R_{a,j}^{2}}{d_{ij}^{2}+1}+20,\; & if\;{0.6R_{r,j}\leq d}_{ij}<R_{r,j}\\ 0, & otherwise \end{array}\right.$$

where $f_{r}(i,j)$ represents the radar threat cost; $f_{a}(i,j)$ represents the artillery threat cost; $d_{ij}$ denotes the shortest three-dimensional Euclidean distance from trajectory segment i of the UAV to threat center j; $R_{r,j}$ represents the effective radius of radar threat zone j; $R_{a,j}$ denotes the effective radius of artillery threat zone j; $N_{r}$ indicates the number of radar threat zones; and $N_{a}$ denotes the number of artillery threat zones.

#### 2.3.4. No-Fly Zone Constraint Cost

The no-fly zone constraint cost $f_{4}$ compels UAVs to avoid designated prohibited areas, which are modeled as vertical cylinders extending from the ground to a specified height. The formulation of $f_{4}$ is given as follows:

(17) $$\;f_{4}=\sum_{i=1}^{N_{uav}}\sum_{j=1}^{N_{nfz}}\sum_{k=1}^{n_{i}}f_{nfz}\left(i,j,k\right)$$

(18) $$\;f_{nfz}\left(i,j,k\right)=\left\{\begin{array}{cc} 1000, & if\;d_{2D}\left(i,j,k\right)\leq R_{nfz,j}\;and\;H_{t}\leq z_{i,k}\leq H_{t}+H_{nfz}\\ 20, & {if\;R_{nfz,j}<d}_{2D}\left(i,j,k\right)\leq 1.2R_{nfz,j}\;and\;H_{t,j}\leq z_{i,k}\leq H_{t,j}+H_{nfz}\\ 0, & otherwise \end{array}\right.$$

where $f_{nfz}\left(i,j,k\right)$ denotes the constraint cost of the kth trajectory segment of the ith drone in the jth no-fly zone; $N_{nfz}$ represents the number of no-fly zones; $d_{2D}(i,j,k)$ is the shortest distance from the projection of the kth trajectory segment of the ith drone onto the xy-plane to the center of the jth no-fly zone; $R_{nfz,j}$ is the cylindrical radius of the jth no-fly zone; $H_{nfz}$ denotes the cylindrical height of the no-fly zone; and $H_{t,j}$ represents the terrain height of the jth no-fly zone.

#### 2.3.5. Time Synchronization Cost

The time synchronization cost $f_{5}$ ensures multiple UAVs arrive at their respective target points within a reasonable time window, achieving temporal coordination. $f_{5}$ is defined as

(19) $$\;\;f_{5}=\sum_{i=1}^{N_{uav}}∆t_{i}$$

(20) $$∆t_{i}=\left|T_{s,i}-\frac{L_{i}}{v_{i}}\right|$$

(21) $$T_{s,i}=\frac{\frac{L_{i}}{v_{max,i}}+\frac{L_{i}}{v_{min,i}}}{2}$$

where $∆t_{i}$ represents the time deviation of the ith drone; $T_{s,i}$ denotes the synchronization target time for the ith drone; $L_{i}$ indicates the path length of the ith drone; $v_{i}$ is the actual flight time of the ith drone; $v_{max}$ and $v_{min}$ respectively denote the maximum and minimum speeds of the ith drone.

#### 2.3.6. Collision Avoidance Cost

The collision avoidance cost $f_{6}$ ensures multiple UAVs maintain safe distances simultaneously to prevent mid-air collisions. $f_{6}$ is defined as

(22) $$\;f_{6}=\sum_{i=1}^{N_{uav}-1}\sum_{j=k+1}^{N_{uav}}\sum_{k=1}^{\min\left(n_{i},n_{j}\right)}f_{col}\left(i,j,k\right)$$

(23) $$f_{col}\left(i,j,k\right)=\left\{\begin{array}{c} \begin{array}{cc} {\left(50-d_{ij,k}\right)}^{2}+500, & if\;d_{ij,k}<30 \end{array}\\ \begin{array}{cc} {\left(50-d_{ij,k}\right)}^{2}+100, & if\;30\leq d_{ij,k}<40 \end{array}\\ \begin{array}{c} \begin{array}{cc} {\left(50-d_{ij,k}\right)}^{2}+20, & if\;40\leq d_{ij,k}<50 \end{array}\\ \begin{array}{cc} 0, & otherwise \end{array} \end{array} \end{array}\right.$$

where $f_{col}\left(i,j,k\right)$ represents the collision avoidance cost between UAV i and UAV j at time k; $d_{ij,k}$ denotes the Euclidean distance between UAV i and UAV j at time k.

#### 2.3.7. Angular Constraint Cost

The angular constraint cost $f_{7}$ limits the turn angle between consecutive waypoints in a UAV’s flight path, ensuring the planned route aligns with the UAV’s physical maneuverability. $f_{7}$ is defined as

(24) $$\;f_{7}=\sum_{i=1}^{U_{uav}}\sum_{k=1}^{n_{i}-1}\left\{\begin{array}{c} \begin{array}{cc} 10, & if\;\alpha_{i,k}>\alpha_{max} \end{array}\\ \begin{array}{cc} 0, & \;\;otherwise \end{array}\;\;\;\; \end{array}\right.$$

where $\alpha_{i,k}$ represents the turn angle of the ith UAV at the kth path point; $\alpha_{max}$ denotes the maximum allowable turn angle.

#### 2.3.8. Trajectory Segment Constraint Cost

To prevent overly dense and impractical paths, each trajectory segment must possess sufficient length to meet the minimum step size requirement for flight control. The trajectory segment constraint cost $f_{8}$ is defined as

(25) $$\;f_{8}=\sum_{i=1}^{N_{uav}}\sum_{k=1}^{n_{i}}\left\{\begin{array}{cc} 5, & if\;\left\|P_{i,k+1}-P_{i,k}\right\|<L_{min}\\ 0, & otherwise \end{array}\right.$$

where $L_{min}$ is the minimum allowable track segment length.

## 3. Aquila Optimizer (AO)

AO mimics four different hunting strategies of the aquila to establish a mathematical model. Based on the varying characteristics of solutions within the search space, it flexibly applies different search strategies. Table 1 lists the symbols and naming conventions used by AO.

### 3.1. Expanded Exploration

In this phase, the eagle flies at high altitude and extensively explores the search space. Once the eagle identifies the area where prey is located, it executes a vertical dive. The mathematical model for this behavior is expressed as

(26) $$X_{1}\left(t+1\right)=X_{best}\left(t\right)\cdot \left(1-\frac{t}{T}\right)+\left[X_{M}\left(t\right)-X_{best}\left(t\right)\right]\cdot rand$$

(27) $$X_{M}\left(t\right)=\frac{1}{N}\sum_{i=1}^{N}X_{i}\left(t\right),\;\;\;\;\;\;\forall j=1,2,\cdots ,Dim$$

where $X_{1}(t+1)$ is the solution of the next iteration for $X_{1}(t$).

### 3.2. Narrowed Exploration

After identifying the prey’s area from high altitude, the eagle hovers above the target prey, prepares to land, and then launches an attack. This method is called contour flight followed by a short glide attack. The mathematical expression for this behavior is

(28) $${\;\;\;\;\;\;\;X}_{2}\left(t+1\right)=X_{best}\left(t\right)\cdot Levy\left(D\right)+X_{R}\left(t\right)+\left(y-x\right)\cdot rand$$

(29) $$Levy\left(D\right)=s\cdot \frac{\zeta \cdot \sigma }{v^{\frac{1}{\beta }}}$$

(30) $$\;\;\sigma =\frac{\Gamma \left(1+\beta \right)\cdot sine\left(\frac{\pi \beta }{2}\right)}{\Gamma \left(\frac{1+\beta }{2}\right)\cdot \beta \cdot 2^{\frac{\beta -1}{2}}}$$

where $X_{2}(t+1)$ is the solution for the next iteration of $X_{2}(t)$; $X_{R}(t+1)$ is a random solution taken within the range at the tth iteration; s is a constant fixed at 0.01; and β is a constant fixed at 1.5.

The spiral shape in the search is represented by y and x, calculated as follows:

(31) $$\;\;y=r\cdot \cos\left(\theta \right)$$

(32) $$\;x=r\cdot \sin\left(\theta \right)$$

(33) $$\;r=r_{1}+U\cdot D_{1}$$

(34) $$\theta =-\omega \cdot D_{1}+\frac{3\pi }{2}$$

where $r_{1}$ takes a value between 1 and 20 to fix the number of search cycles; U is fixed at 0.00565; $D_{1}$ is an integer from 1 to the search space length Dim; and ω is a small fixed value of 0.005.

### 3.3. Expanded Exploitation

After precisely identifying the prey’s area, the eagle performs an initial vertical descent to test the prey’s reaction. This method is called the low-flying slow descent attack. The mathematical expression for this behavior is

(35) $$X_{3}\left(t+1\right)=\alpha \cdot \left(X_{best}\left(t\right)-X_{M}\left(t\right)\right)-rand+\delta \cdot \left(\left(UB-LB\right)\cdot rand+LB\right)$$

where $X_{3}\left(t+1\right)$ is the solution for the next iteration of $X_{3}\left(t\right)$; α and δ  are adjustment parameters, typically set to 0.1.

### 3.4. Narrowed Exploitation

When the eagle approaches its prey, it chases and attacks the prey on the ground. The mathematical expression for this behavior is

(36) $$X_{4}\left(t+1\right)=QF\cdot X_{best}\left(t\right)-G_{1}\cdot X\left(t\right)\cdot rand-G_{2}\cdot Levy\left(D\right)+rand\cdot G_{1}$$

(37) $$QF\left(t\right)=t^{\frac{2\cdot rand-1}{{\left(1-T\right)}^{2}}}$$

(38) $$G_{1}=2\cdot rand-1$$

(39) $$G_{2}=2\cdot \left(1-\frac{t}{T}\right)$$

where $X_{4}\left(t+1\right)$ is the solution for the next iteration of $X_{4}\left(t\right)$.

## 4. Behavior-Adaptive Aquila Optimizer (EAO)

Although Aquila Optimizer provides an effective framework for solving complex optimization problems by simulating the hunting behavior of the eagle, it still has inherent limitations when addressing high-dimensional, multimodal, and other complex scenarios. Firstly, its behavior switching relies on fixed iteration progress rather than real-time search states, which prevents the search process from adapting to the dynamic characteristics of the problem. This leads to difficulties in sustaining effective strategies and results in low search efficiency. Secondly, the algorithm lacks effective diversity maintenance and distributed guidance mechanisms. The population tends to rapidly homogenize under the attraction of a single global optimal solution, making it prone to falling into local optima when tackling complex optimization problems. Additionally, the randomness in its local search strategies results in insufficient exploitation precision and stability.

**Figure 2:**
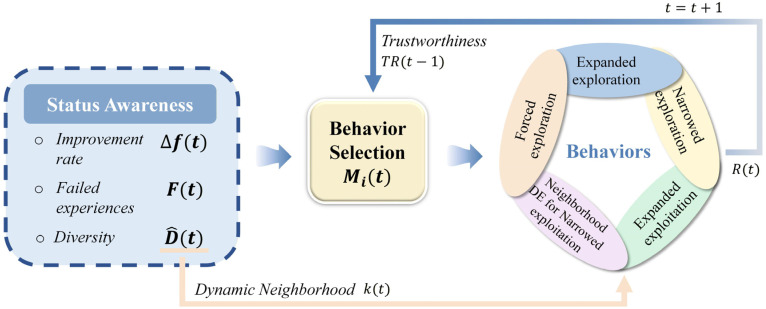
Schematic diagram of the EAO algorithm’s behavior-adaptive collaborative optimization framework.

To address these systematic limitations, this section proposes an enhanced version of Aquila Optimizer—the Adaptive Aquila Optimizer (EAO). Its core improvements lie in introducing a series of adaptive mechanisms based on search-state awareness and deep collaboration among strategies: Firstly, a behavior selection and trust mechanism based on search-state awareness is designed to dynamically determine the timing for behavior switching, ensuring the continuity of effective strategies. Secondly, periodic diversity maintenance and dynamic neighborhood guidance strategies are introduced to avoid premature convergence while fully utilizing local search information. Lastly, a walking predation behavior based on neighborhood differential evolution is adopted to enhance the stability and efficiency of local search. These strategies collectively form an adaptive and collaborative optimization framework to improve the algorithm’s ability to balance global exploration and local exploitation. The schematic diagram illustrating the behavior adaptation principle of EAO, composed of multiple synergistic strategies, is shown in Figure 2. Table 2 lists the symbols and naming conventions used by EAO.

### 4.1. Periodic Diversity Maintenance Mechanism

The original AO lacks an active diversity maintenance strategy, which can lead to rapid loss of population diversity and a tendency to fall into local optima. Relying solely on random perturbations makes it difficult to escape local optima. Therefore, a periodically triggered diversity maintenance mechanism is introduced.

The population diversity index is defined as shown in Equation (40). It reflects the dispersion degree of the population [35]. A high value indicates an exploration phase, while a low value indicates an exploitation phase.

(40) $$D\left(t\right)=\frac{1}{N}\sum_{i=1}^{N}\left\|X_{i}\left(t\right)-\bar{X}\left(t\right)\right\|$$

(41) $$\bar{X}\left(t\right)=\frac{1}{N}\sum_{i=1}^{N}X_{i}\left(t\right)$$

(42) $$\hat{D}\left(t\right)=\frac{D\left(t\right)}{\left\|UB-LB\right\|}$$

where $\left\|\cdot \right\|$ denotes the Euclidean norm.

The mechanism is configured to check $\hat{D}\left(t\right)$ every $R_{P}$ iterations. Based on the results of the $R_{P}$ parameter sensitivity test (see Appendix A.2, Table A3), set $R_{P}=15$. If it falls below a threshold, selective resetting is applied to the worst 15% of individuals in terms of fitness. Specifically, individuals with fitness values greater than 1.1 times the current best fitness are reset. The reset individual position $X_{reset}$ is given by

(43) $$\;X_{reset}=LB+rand\left(Dim\right)⊙\left(UB-LB\right)$$

where rand(Dim) is a uniformly distributed random vector of dimension Dim.

### 4.2. Diversity-Based Dynamic Neighborhood Guidance Mechanism

In the original AO, all individuals are guided solely by the single global best solution $X_{best}\left(t\right)$, which can lead to rapid population clustering and loss of diversity. This increases the risk of premature convergence and fails to fully utilize local information from different spatial regions. To address this issue, EAO replaces the global best with a neighborhood-best approach in both the expanded exploration and expanded exploitation phases of the original AO. Additionally, the neighborhood size is dynamically adjusted based on population diversity, preventing excessive population concentration during these phases.

Let the neighborhood $N_{i}^{k}(t)$ of individual i consist of its k nearest neighbors among the other individuals. The neighborhood best position $X_{lbest}^{i}(t)$ for individual i is then defined as

(44) $$X_{lbest}^{i}\left(t\right)=\begin{array}{cc} \arg minf_{j}(t), & j\in \end{array}N_{i}^{k}\left(t\right)$$

The neighborhood size k(t) dynamically adjusts based on $\hat{D}\left(t\right)$:

(45) $$\;k\left(t\right)=\left\{\begin{array}{cc} k_{max}, & if\;\hat{D}\left(t\right)<\theta_{L}\\ k_{min}, & if\;\hat{D}\left(t\right)>\theta_{H}\\ k_{max}-\left\lfloor \frac{\hat{D}\left(t\right)-\theta_{L}}{\theta_{H}-\theta_{L}}\left(k_{max}-k_{min}\right)\right\rfloor , & otherwise \end{array}\right.$$

(46) $$k_{max}=N\cdot \mu$$

Therefore, replacing the global optimal position with the neighborhood optimal position in Equations (26) and (35) from Phase 1 and Phase 3 of the AO algorithm yields

(47) $${\;\;\;\;\;\;\;\;X}_{1}\left(t+1\right)=X_{lbest}\left(t\right)\cdot \left(1-\frac{t}{T}\right)+\left[X_{M}\left(t\right)-X_{lbest}\left(t\right)\right]\cdot rand$$

(48) $${\;\;\;X}_{3}\left(t+1\right)=\alpha \cdot \left(X_{lbest}\left(t\right)-X_{M}\left(t\right)\right)-rand+\delta \cdot \left(\left(UB-LB\right)\cdot rand+LB\right)$$

### 4.3. Narrowed Exploitation Behavior Based on Neighborhood Differential Evolution

The narrowed exploitation formula in the original AO is complex and relies on Lévy flights. The long-jump property of Lévy flights can lead to instability during the local refinement search phase, resulting in insufficiently clear search directions. To address this, EAO replaces Equation (36) with a neighborhood-based differential mutation operator DE/current−to−lbest/1 [36]:

(49) $$X_{4}\left(t+1\right)=X\left(t\right)+F\cdot \left(X_{lbest}\left(t\right)-X\left(t\right)\right)+F\cdot (X_{r1}(t)-X_{r2}(t))$$

where $X_{r1}\left(t\right)$ and $X_{r2}(t)$ are two distinct individual positions randomly selected from the neighborhood.

### 4.4. Adaptive Behavior Selection Mechanism Based on Search-State Awareness

The original AO adopts a behavior-switching strategy based on fixed iteration progress: exploration behaviors are randomly selected when t/T≤2/3, and exploitation behaviors are randomly selected when t/T>2/3. However, this mechanism has inherent limitations. First, the search state can vary significantly across different problems at the same iteration progress. Fixed switching thresholds fail to adapt to the dynamic characteristics of the problem. Second, the strategy cannot adjust behavioral choices based on search effectiveness, potentially causing effective behaviors to be prematurely abandoned or ineffective behaviors to be over-executed, lacking adaptivity.

To address these issues, EAO introduces an adaptive behavior selection mechanism based on search-state awareness. This mechanism dynamically selects exploration or exploitation behaviors according to the current search state, thereby improving the algorithm’s ability to balance exploitation and exploration.

The adaptive behavior selection mechanism achieves self-adaptive decision-making by

- Introducing three core search-state metrics to monitor the optimization process in real-time;
- Establishing behavior-selection rules and a trust mechanism based on these states;
- Incorporating a forced exploration behavior to ensure adaptability.

#### 4.4.1. Definition of Search-State Quantities

(1) Optimal Solution Improvement Rate

The optimal solution improvement rate Δf(t) is defined to reflect search effectiveness. A high value indicates the current search direction is effective, while a low value suggests potential stagnation.

(50) $$∆f\left(t\right)=\left|f_{best}\left(t\right)-f_{best}\left(t-\tau \right)\right|$$

(2) Failure Experience Metric

Define the failure experience metric F(t) to reflect search stability. A high value indicates that most individuals failed to achieve improvement, suggesting the search may be trapped in local optima.

(51) $$F\left(t\right)=\frac{1}{N}\sum_{i=1}^{N}\mathrm{II}\left\{f_{i}\left(t\right)\geq f_{i}\left(t-1\right)\right\}$$

where II(·) denotes the indicator function, which returns 1 when the condition is true and 0 otherwise.

#### 4.4.2. State-Driven Behavior Selection Rules

Based on the state variable combinations defined in Section 2.1, these are mapped to six behavioral modes. The six behaviors are: expanded exploration, narrowed exploration, expanded exploitation, and narrowed exploitation from AO; and the newly defined forced exploration and random exploitation from EAO.

In EAO, forced exploration refers to the scenario where, if the eagle fails to capture prey in the current hunting area over an extended period, the situation indicates the area is depleted. The eagle then leaves the current hunting ground and re-explores the nearby region. The formula for generating a new position in this behavior is

(52) $$X_{new}\left(t\right)=X_{best}\left(t\right)+\left(UB-LB\right)⊙\left(rand\left(Dim\right)-0.5\right)\cdot \eta \left(t\right)$$

(53) $$\;\;\eta \left(t\right)=\eta_{max}\cdot \left(1-\frac{t}{T}\right)$$

where $\eta_{max}$ is its maximum value, set to $\eta_{max}=1$.

Thus, the forced exploration behavior performs a substantial random jump centered around the current best solution within a limited range. This approach leverages known information while retaining the ability to escape local traps.

Random exploitation refers to randomly selecting either expanded exploitation or narrowed exploitation.

The six behaviors are numbered from 1 to 6, and their selection rules are defined as

(54) $$M_{i}^{s}\left(t\right)=\left\{\begin{array}{cc} 1, & if\;\hat{D}\left(t\right)>\theta_{D}\;and\;∆f\left(t\right)<\theta_{∆f}\\ 2, & if\;\hat{D}\left(t\right)>\theta_{D}\;and\;∆f\left(t\right)\geq \theta_{∆f}\\ 3, & if\;\hat{D}\left(t\right)\leq \theta_{D}\;and\;∆f\left(t\right)\geq \theta_{∆f}\;and\;F\left(t\right)>\theta_{F}\\ 4, & if\;\hat{D}\left(t\right)\leq \theta_{D}\;and\;∆f\left(t\right)\geq \theta_{∆f}\;and\;F\left(t\right)\leq \theta_{F}\\ 5, & if\;\hat{D}\left(t\right)\leq \theta_{D}\;and\;∆f\left(t\right)<\theta_{∆f}\;and\;F\left(t\right)>\theta_{F}\\ rand\left\{3,4\right\}, & \;if\;\hat{D}\left(t\right)\leq \theta_{D}\;and\;∆f\left(t\right)<\theta_{∆f}\;and\;F\left(t\right)\leq \theta_{F} \end{array}\right.$$

where $\theta_{D}$, $\theta_{∆f}$, $\theta_{F}$ represent the critical thresholds for behavior selection based on $\hat{D}\left(t\right)$, $∆f\left(t\right)$ and $F\left(t\right)$, respectively, with values set as $\theta_{D}=0.3$, $\theta_{D}=0.3$, and $\theta_{F}=0.5$.

#### 4.4.3. Trust-Based Behavioral Persistence Mechanism

The original AO algorithm exhibits strong randomness and a lack of continuity in behavior selection. Even if the current search behavior is effective, it may be randomly switched in the next generation, leading to inconsistent search directions and low convergence efficiency. To address this, this mechanism introduces the concept of “trust” to enable the algorithm to adaptively extend the execution time of effective behaviors based on historical performance and promptly discard ineffective ones.

The trust-based behavior decision logic is defined as

(55) $$M_{i}\left(t\right)=\left\{\begin{array}{cc} M_{i}^{s}\left(t\right), & if\;t=1\;or\;{TR}_{i}\left(t-1\right)=0\\ M_{i}\left(t-1\right), & \;if\;{TR}_{i}\left(t-1\right)>0 \end{array}\right.$$

where ${TR}_{i}\left(t-1\right)$ is the behavior trust, representing the remaining number of iterations for which this behavior can continue to be executed.

When the trust ${TR}_{i}\left(t-1\right)>0$, the individual will continue executing the previous generation’s behavior. When the trust is depleted (${TR}_{i}\left(t-1\right)=0$), it switches to a new suggested behavior.

After each generation of search, rewards or penalties are applied based on whether the individual improves. If the fitness value of the new position is better than the original position, a reward is executed. The trust update formulas are as follows:

(56) $$S_{i}\left(t\right)=S_{i}\left(t-1\right)+1$$

(57) $${TR}_{i}\left(t\right)=\min\left\{{TR}_{i}\left(t-1\right)+R\left(S_{i}\left(t\right)\right),{TR}_{max}\right\}$$

(58) $$R\left(S_{i}\left(t\right)\right)=\left\{\begin{array}{c} \begin{array}{cc} 3, & if\;S_{i}\left(t\right)\geq 2 \end{array}\\ \begin{array}{cc} 2, & if\;S_{i}\left(t\right)=1 \end{array} \end{array}\right.$$

where R(⋅) is the reward function.

If the fitness value of the new position is not better than the original position, a penalty is executed. The trust update formulas in this case are

(59) $$S_{i}\left(t\right)=0$$

(60) $${TR}_{i}\left(t\right)=\max\left\{T_{i}\left(t-1\right)-1,{TR}_{min}\right\}$$

### 4.5. Pseudocode and Program Flowchart

**Figure 3:**
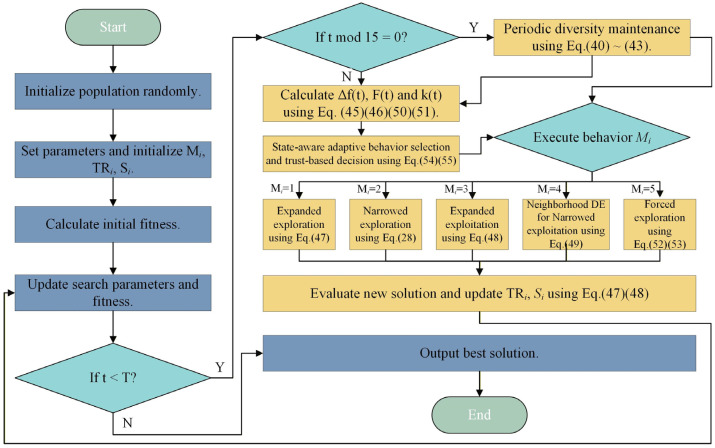
EAO algorithm flowchart.

The EAO algorithm integrates a periodic diversity maintenance mechanism, a diversity-based dynamic neighborhood guidance mechanism, a narrowed exploitation behavior based on neighborhood differential evolution, and an adaptive behavior selection mechanism based on search-state awareness. The flowchart of the EAO algorithm which enables adaptive behavior selection is shown in Figure 3. The time complexity analysis is provided in Appendix A.1, and the pseudocode of EAO (Algorithm 1) is as follows:

**Algorithm 1** Pseudocode for EAO

$\mathrm{Input}\text{:}\;\mathrm{Population}\;\mathrm{size}\;N,\;\mathrm{maximum}\;\mathrm{evaluations}\;{Max}_{FES},\;\mathrm{bounds}\;lb\;\text{\&}\;ub,\;\mathrm{dimension}\;dim,\;\mathrm{objective}\;\mathrm{function}\;objfun$



$\mathrm{Output}\text{:}\;\mathrm{Best}\;\mathrm{solution}\;X_{best},\;\mathrm{best}\;\mathrm{fitness}\;{Best}_{FF}$



$\mathrm{Set}\;\mathrm{parameters}\;\mathrm{of}\;\mathrm{the}\;\mathrm{EAO}\;(\text{i.e.},\;\tau ,\;k_{min},\;\text{etc.}).$

1: Initialize population randomly and evaluate fitness.
$2\text{:}\;\mathrm{Initialize}\;\mathrm{behavior}\;\mathrm{memory}\;M_{i},\;\mathrm{trust}\;{TR}_{i},\;\mathrm{success}\;\mathrm{count}\;S_{i}\;\mathrm{for}\;\mathrm{each}\;\mathrm{individual}.$
$3\text{:}\;\mathrm{Set}\;Best\_FF=\min(\mathrm{Fitness}),\;X_{best}\;\mathrm{correspondingly},\;t=0$
4: while  t≤ T do
5:          t = t + 1


6:           if t mod 15=0 then



7:                if c[a]>0 then

$8\text{:}\;\;\;\;\;\;\;\;\;\;\;\;\;\;\;\;\;\;\;\;\;\;\;\;\mathrm{Calculate}\;D\left(t\right),\;\bar{X}\left(t\right),\;\hat{D}\left(t\right),\;p\left(a\right)$ using Equations (40)–(42).

$9\text{:}\;\;\;\;\;\;\;\;\;\;\;\;\;\;\;\;\;\;\;\;\;\;\;\;\;\;\mathrm{if}\;\hat{D}\left(t\right)<0.1\;\mathrm{then}$

10:                            Reinitialize worst 15% individuals using Equation (43).11:                              Evaluate new individuals and update ${Best}_{FF}$12:                        end if13:                end if14:          Calculate Δf(t) and F(t) using Equations (50) and (51).15:          Determine k(t) using Equations (45) and (46).$16\text{:}\;\;\;\;\;\;\;\;\;\;\mathrm{Calculate}\;\mathrm{AO}\;\mathrm{parameters}\;G_{1}\;\mathrm{and}\;G_{2}$ using Equations (38) and (39).17:          for i = 1 to N do$18\text{:}\;\;\;\;\;\;\;\;\;\;\;\;\;\;\;\;\;\;\;\;\;\;\mathrm{Calculate}\;\mathrm{distances}\;\mathrm{from}\;X_{i}$ to all individuals, sort and select k(t) neighbors.$19\text{:}\;\;\;\;\;\;\;\;\;\;\;\;\;\;\;\;\;\;\;\;\;\;\mathrm{Obtain}\;X_{lbest}^{i}(t)$ using Equation (44).$20\text{:}\;\;\;\;\;\;\;\;\;\;\;\;\;\;\;\;\;\;\;\;\;\;\mathrm{Determine}\;\mathrm{suggested}\;\mathrm{behavior}\;M_{i}^{s}\left(t\right)$ using Equation (54).$21\text{:}\;\;\;\;\;\;\;\;\;\;\;\;\;\;\;\;\;\;\;\;\;\;\mathrm{Select}\;\mathrm{actual}\;\mathrm{behavior}\;M_{i}\left(t\right)$ using Equation (55).$22\text{:}\;\;\;\;\;\;\;\;\;\;\;\;\;\;\;\;\;\;\;\;\;\;\;\;\;\;\mathrm{if}\;M_{i}\left(t\right)=1$ then$23\text{:}\;\;\;\;\;\;\;\;\;\;\;\;\;\;\;\;\;\;\;\;\;\;\;\;\;\;\;\;\;\;\;\;\;\;\;\mathrm{Update}\;X_{i}^{new}$ using Equation (47).$24\text{:}\;\;\;\;\;\;\;\;\;\;\;\;\;\;\;\;\;\;\;\;\;\;\;\;\;\;\mathrm{else}\;\mathrm{if}\;M_{i}\left(t\right)=2$ then$25\text{:}\;\;\;\;\;\;\;\;\;\;\;\;\;\;\;\;\;\;\;\;\;\;\;\;\;\;\;\;\;\;\;\;\;\;\;\mathrm{Update}\;X_{i}^{new}$ using Equation (28).$26\text{:}\;\;\;\;\;\;\;\;\;\;\;\;\;\;\;\;\;\;\;\;\;\;\;\;\;\;\mathrm{else}\;\mathrm{if}\;M_{i}\left(t\right)=3$ then$27\text{:}\;\;\;\;\;\;\;\;\;\;\;\;\;\;\;\;\;\;\;\;\;\;\;\;\;\;\;\;\;\;\;\;\;\;\;\mathrm{Update}\;X_{i}^{new}$ using Equation (48).$28\text{:}\;\;\;\;\;\;\;\;\;\;\;\;\;\;\;\;\;\;\;\;\;\;\;\;\;\;\mathrm{else}\;\mathrm{if}\;M_{i}\left(t\right)=4$ then$29\text{:}\;\;\;\;\;\;\;\;\;\;\;\;\;\;\;\;\;\;\;\;\;\;\;\;\;\;\;\;\;\;\;\;\;\;\;\mathrm{Select}\;X_{r1},\;X_{r2}\;\mathrm{from}\;k(t)$ neighbors.$30\text{:}\;\;\;\;\;\;\;\;\;\;\;\;\;\;\;\;\;\;\;\;\;\;\;\;\;\;\;\;\;\;\;\;\;\;\;\mathrm{Update}\;X_{i}^{new}$ using Equation (49)$31\text{:}\;\;\;\;\;\;\;\;\;\;\;\;\;\;\;\;\;\;\;\;\;\;\;\;\;\;\mathrm{else}\;\mathrm{if}\;M_{i}\left(t\right)=5$ then$32\text{:}\;\;\;\;\;\;\;\;\;\;\;\;\;\;\;\;\;\;\;\;\;\;\;\;\;\;\;\;\;\;\;\;\;\;\;\mathrm{Update}\;X_{i}^{new}$ using Equations (52) and (53).33:                       else$34\text{:}\;\;\;\;\;\;\;\;\;\;\;\;\;\;\;\;\;\;\;\;\;\;\;\;\;\;\;\;\;\;\;\;\;\;\;\mathrm{Randomly}\;\mathrm{choose}\;M_{i}\left(t\right)\;\in \;\{3,4\}$ and apply corresponding update.35:                       end if

$36\text{:}\;\;\;\;\;\;\;\;\;\;\;\;\;\;\;\;\;\;\;\;\;\;\;\;\;\mathrm{Apply}\;\mathrm{boundary}\;\mathrm{handling}\;\mathrm{to}\;X_{i}^{new}\;\mathrm{and}\;\mathrm{evaluate}\;{fitness}_{i}^{new}$

$37\text{:}\;\;\;\;\;\;\;\;\;\;\;\;\;\;\;\;\;\;\;\;\;\;\;\;\;\mathrm{Update}\;S_{i}\left(t\right)\;\mathrm{and}\;{TR}_{i}(t)$ using Equations (59) and (60).
$38\text{:}\;\;\;\;\;\;\;\;\;\;\;\;\;\;\;\;\;\;\;\;\;\;\;\;\;\mathrm{Update}\;X_{best},{Best}_{FF}$ if better solution found.
39:           end for40: end while

$41\text{:}\;\mathrm{Return}\;X_{best},{Best}_{FF}$



## 5. Parameter Sensitivity Analysis of EAO

Parameter selection is crucial for the performance of metaheuristic algorithms. Therefore, this section conducts a parameter sensitivity analysis on six key parameters in EAO using 12 functions from the CEC2017 test suite: F1, F3, F6, F9, F11, F12, F13, F15, F24, F27, F29 and F30. The analyzed parameters include population size N, historical window length τ, minimum neighborhood size $k_{min}$, maximum neighborhood size percentage μ, low diversity threshold $\theta_{L}$, and high diversity threshold $\theta_{L}$.

**Figure 4:**
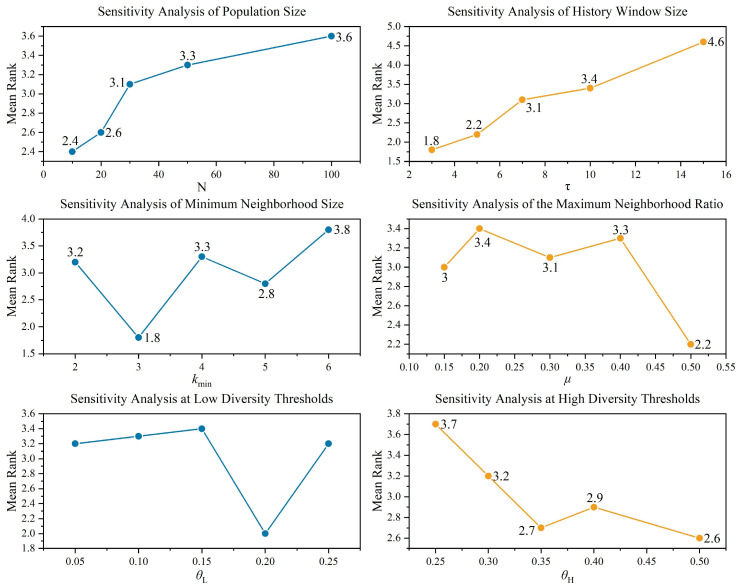
Friedman’s average rank for different values of parameters.

During the experiments, each parameter is varied within its predefined range while the others remain fixed, to explore the impact of different values on EAO’s performance. The maximum number of function evaluations is set to 1000 × Dim. To avoid randomness, each experiment is repeated 30 times. The best value, average value, variance, and Friedman ranking are calculated. The overall Friedman ranking comparison is shown in Figure 4, and detailed data is provided in Table A4, Table A5, Table A6, Table A7, Table A8 and Table A9 of Appendix A.2.

According to the experimental results, the best overall Friedman ranking is achieved when τ=3, $k_{min}=3$, $\mu =0.5,\theta_{L}=0.2,and\;\theta_{H}=0.5$. In the sensitivity analysis of population size, EAO performs better with smaller populations. This is because EAO’s adaptive behavior persistence mechanism and diversity maintenance mechanism function more effectively when the number of iterations is sufficient. However, considering the statistical significance of algorithm comparisons, the standard setting N = 30 is adopted for the experiments in this paper.

## 6. Performance Testing and Analysis of EAO

To systematically and comprehensively evaluate the optimization performance and algorithmic characteristics of the proposed EAO algorithm, this chapter conducts multi-angle and multi-level experimental analyses based on the internationally recognized CEC2017 and CEC2020 benchmark test suites. The CEC2017 function set includes various unimodal, multimodal, hybrid, and composite functions that are suitable for assessing an algorithm’s optimization capability in medium- to high-dimensional complex search spaces. The CEC2020 function set presents greater challenges in terms of function construction and variable coupling, often used to verify an algorithm’s generalization ability under different problem characteristics and lower-dimensional conditions. Detailed descriptions of the functions in both test suites are provided in Table A1 and Table A2 of Appendix A.2.

Regarding the design of experimental content: first, multiple EAO variants are constructed, and ablation experiments are conducted on the 30-dimensional test functions of CEC2017 to analyze the effectiveness of each improvement strategy; second, by introducing a population dimensional diversity metric, the exploration and exploitation capabilities of the algorithm are assessed; finally, under different dimension settings, systematic performance comparisons between EAO and multiple competing algorithms are carried out on the CEC2017 (30/50/100 dimensions) and CEC2020 (10/20 dimensions) benchmark test suites, accompanied by statistical significance analysis using the Friedman ranking test and the Wilcoxon rank-sum test.

To thoroughly validate the optimization performance and competitive advantages of EAO, a total of 14 representative optimization algorithms are selected as comparison methods to ensure the comprehensiveness of the results. These include: (1) classic and highly cited algorithms: PSO, DE, CMA-ES (Covariance Matrix Adaptation Evolution Strategy, 2016) [37], WOA, SCA (Sine Cosine Algorithm, 2016) [38], MFO, and HHO (Harris Hawks Optimization, 2019) [39]; (2) recent high-performance algorithms: MShOA (Mantis Shrimp Optimization Algorithm, 2025) [40], GJO, (Weighted Average Algorithm, 2025) [41], SHO (Sea-Horse Optimizer, 2022) [42], HO (Hippopotamus Optimization, 2024) [43], and PO (Parrot Optimizer, 2024) [44]; (3) the original Aquila Optimizer (AO).

### 6.1. Experimental Configuration

The parameter settings of the algorithms refer to their respective original papers, with specific configurations listed in Table 3. All compared algorithms are run under the same experimental conditions: the population size is set to 30, and the maximum number of function evaluations is set to 1000 × Dim. To ensure the fairness and reproducibility of the experimental results, all algorithms are executed 30 independent runs on the benchmark test suites. The best value, mean value, variance, Friedman ranking, and Wilcoxon rank-sum test results are calculated. All experiments are conducted on the software MATLAB R2021b.

### 6.2. Ablation Experiment

To verify the contribution of each improvement strategy in EAO to the algorithm’s performance enhancement, this section conducts ablation experiments on the 30-dimensional test functions of CEC2017. By introducing different improvement mechanisms into the original AO algorithm, various algorithm variants are constructed to analyze the independent role of each strategy in improving search performance. These algorithm variants include: (1) EAO_D: Introduces a periodic diversity maintenance mechanism into the original AO; (2) EAO_N: AO combined with a dynamic neighborhood guidance mechanism based on population diversity; (3) EAO_E: Integrates a neighborhood differential evolution-based search strategy into the narrowed exploitation phase of AO; (4) EAO_S: Introduces a search-state-aware adaptive behavior selection mechanism into AO. By comparing the best values, mean values, and standard deviations of each variant with the complete EAO across test functions, and combining the results of Friedman rankings, the contribution of different improvement strategies to the overall performance of the algorithm can be intuitively assessed.

Table 4 presents the test results of various algorithm variants, including the best value, mean value, variance, and Friedman ranking. The best values in the table are highlighted in bold and underlined.

Overall, compared to other variants and the original AO algorithm, EAO achieves the highest number of best values across the test functions and ranks first overall. This indicates that the improvement strategies, when combined, can effectively enhance the algorithm’s search capability and stability. In contrast, algorithm variants enhanced with a single strategy show some improvement on certain functions, but their overall performance remains weaker than that of EAO. Through comparison of different variants, it can be observed that both EAO_S and EAO_D outperform the original AO in ranking, suggesting that the search-state-aware adaptive behavior selection mechanism and the periodic diversity maintenance mechanism positively impact algorithm performance. Although EAO_N and EAO_E rank lower than AO, they achieve optimal results in some metrics, indicating that the dynamic neighborhood guidance mechanism and the neighborhood differential evolution-based search strategy must be organically integrated with other strategies to achieve optimal optimization performance.

### 6.3. Exploration and Exploitation Experiment

The balance between exploration and exploitation is a key factor determining the performance of swarm intelligence algorithms. To deeply analyze the search behavior characteristics of EAO, as described in this subsection, F1, F4, F6, F10, F11, F20, F22, F25 and F28 are selected from the four types of functions in CEC2017 to quantitatively evaluate the algorithm’s exploration and exploitation capabilities. The population dimensional diversity metric [45,46] is introduced in the experiments to characterize the changes in population distribution during the search process. By analyzing the evolutionary trend of diversity over iterations in EAO, the behavioral characteristics of its global exploration and local exploitation phases can be visually reflected. The formulas for the population dimension diversity index Div and the exploration–exploitation ratio are as follows:

(61) $$\;\;Div=\frac{1}{D}\sum_{j=1}^{D}\frac{1}{N}\left(\sum_{i=1}^{N}\left|median\left(X_{j}\right)-X_{i,j}\right|\right)$$

(62) $$Exploration\%=\frac{Div}{{Div}_{max}}$$

(63) $$Exploitation\%=\frac{\left|Div-{Div}_{max}\right|}{{Div}_{max}}$$

where ${Div}_{max}$ is the maximum population dimension diversity value; $X_{i,j}$ is the position of the ith individual in the jth dimension; and $median\left(X_{j}\right)$ is the median of the positions of all individuals in the jth dimension.

**Figure 5:**
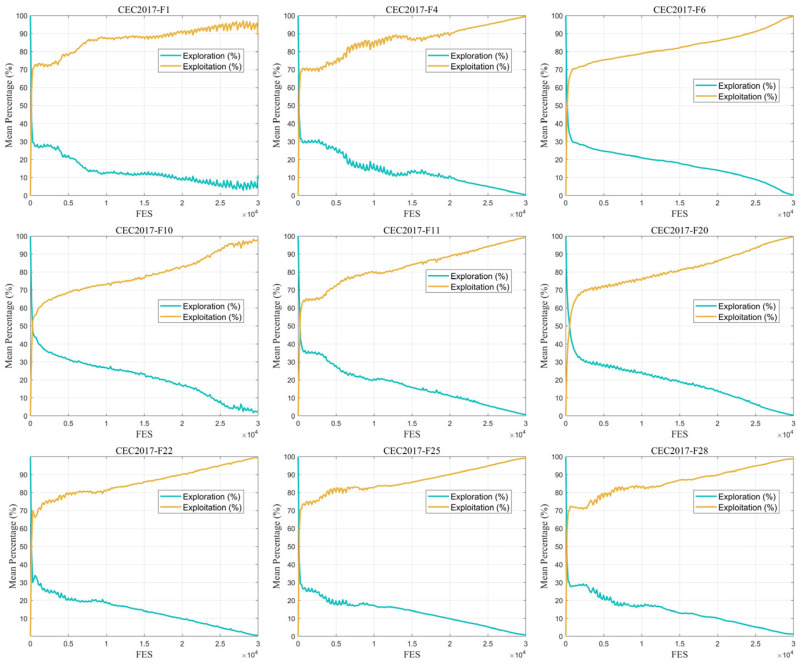
Exploration and exploitation experiment results on selected CEC2017 functions.

Figure 5 shows the exploration and exploitation variation curves of EAO on the CEC2017 30-dimensional test functions, illustrating the dynamic balance between exploration and exploitation capabilities during the search process. From the figure, it can be observed that EAO maintains a relatively high exploration ratio in the early stages of the search, which helps fully explore the search space and avoid premature convergence to local optima. As the iteration process progresses, the exploration ratio gradually decreases while the exploitation ratio increases, indicating that the algorithm can appropriately enhance exploitation capability to accelerate the convergence process.

### 6.4. Convergence Analysis

To further analyze the convergence characteristics of the proposed EAO algorithm, eight functions, including F1, F4, F6, F9, F13, F19, F20, and F25 from the CEC2017 benchmark suite were selected. The convergence behavior of EAO was studied in a 30-dimensional search space. The number of iterations was set to 50 in the experiments. Several results were presented, including the search history distribution, the trajectory changes of five search agents in the first dimension, the average fitness curve of the population, and the convergence curve of the optimal value.

**Figure 6:**
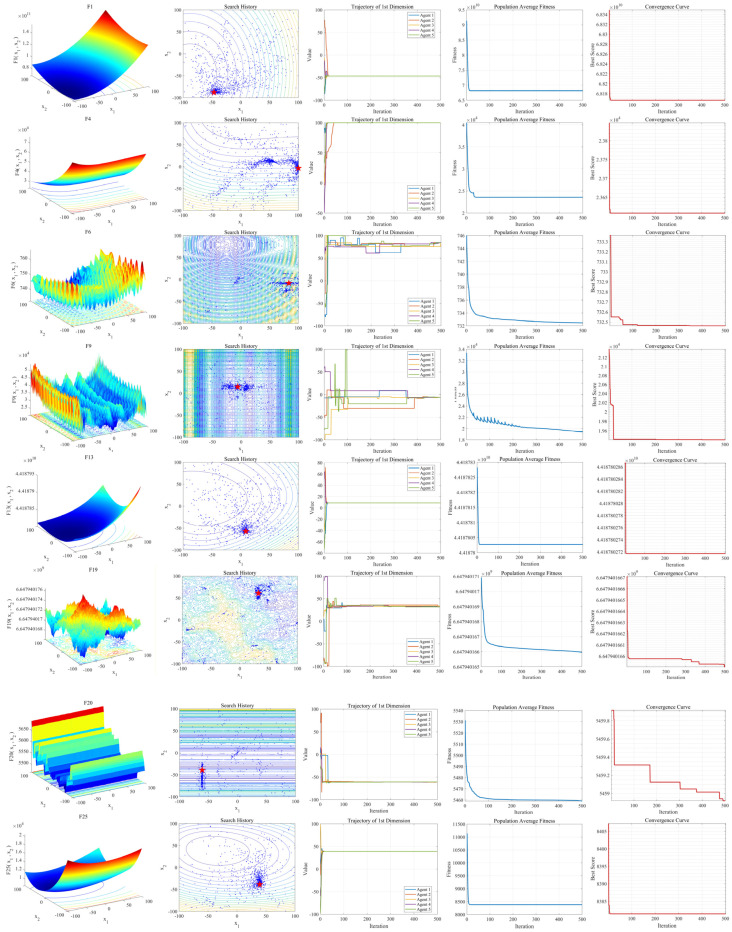
Experimental results of convergence analysis on selected CEC2017 functions.

Figure 6 shows the experimental results in a two-dimensional space. In the search history plots (the second column), the red star represents the global optimum. It can be observed from the distribution of sample points that the points far from the global optimum are sparse and widely scattered. In contrast, the points near the global optimum are densely distributed. This indicates that EAO has strong global exploration capability and can effectively conduct local exploitation in regions containing potential optima.

The trajectories of five search agents in the first dimension are shown in the third column. As the iterations proceed, the variations in the search agents’ values gradually become stable. The average fitness curve of the population (the fourth column) and the convergence curve of the optimal value (the fifth column) both decline steadily throughout the iteration process. This further demonstrates that the EAO algorithm can converge stably to the optimal solution.

### 6.5. Test Results and Analysis on CEC2017

**Figure 7:**
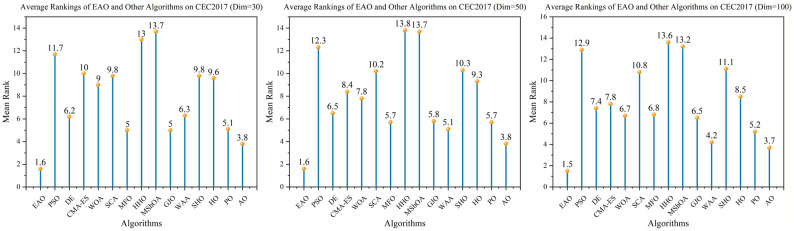
Friedman average rankings of EAO and other algorithms across different dimensions on CEC2017.

**Figure 8:**
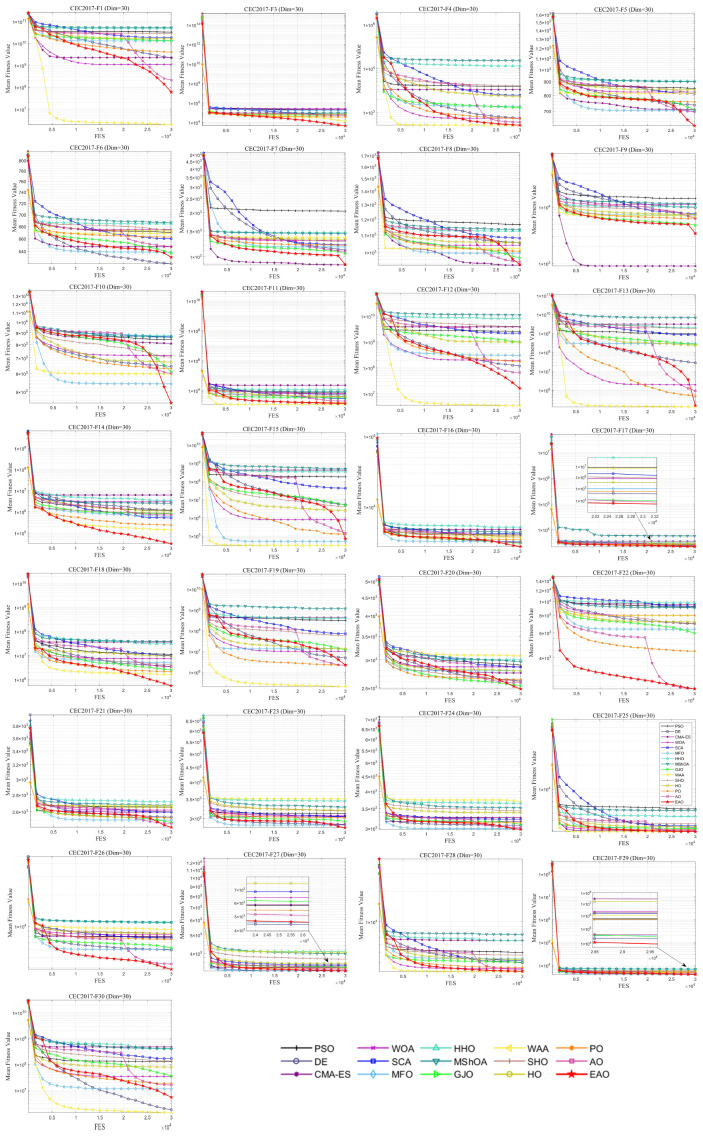
Average iteration curve of EAO and other algorithms on CEC2017 (Dim = 30).

**Figure 9:**
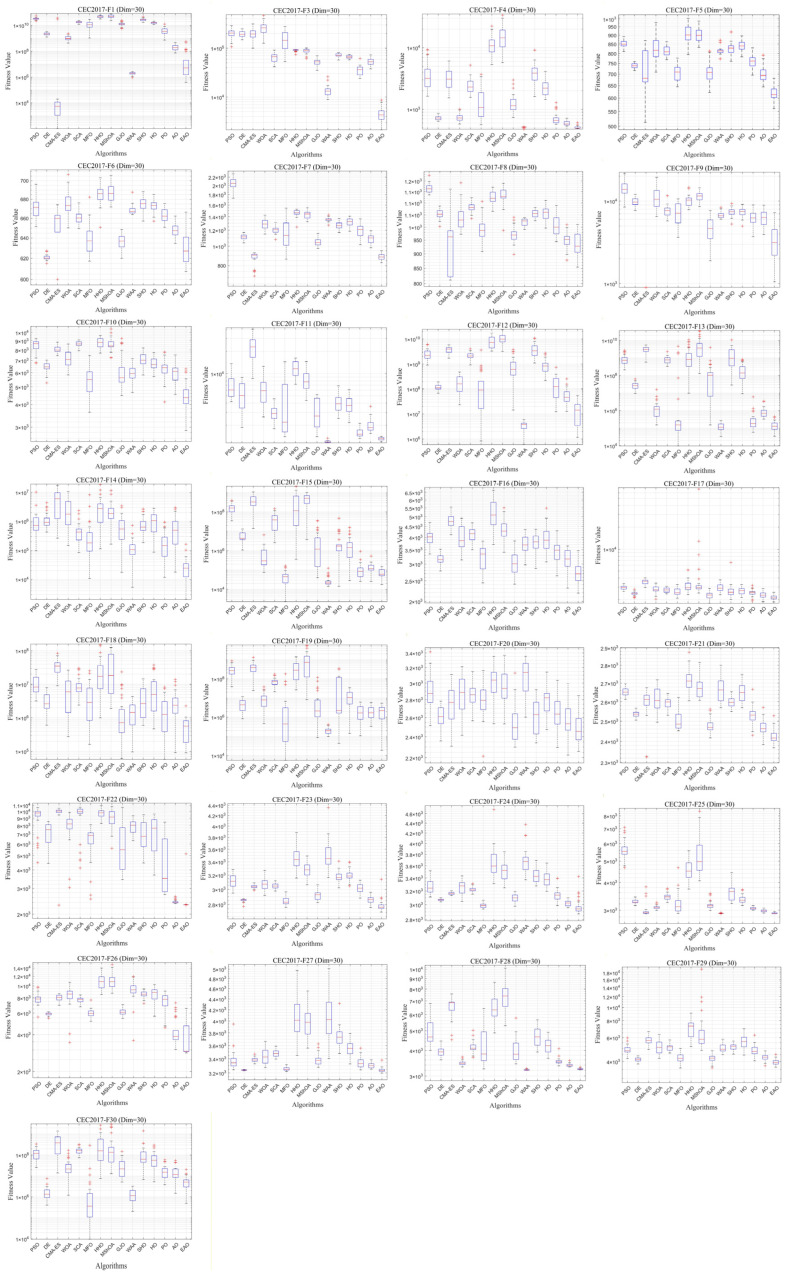
Boxplot of EAO and other algorithms on CEC2017 (Dim = 30).

**Figure 10:**
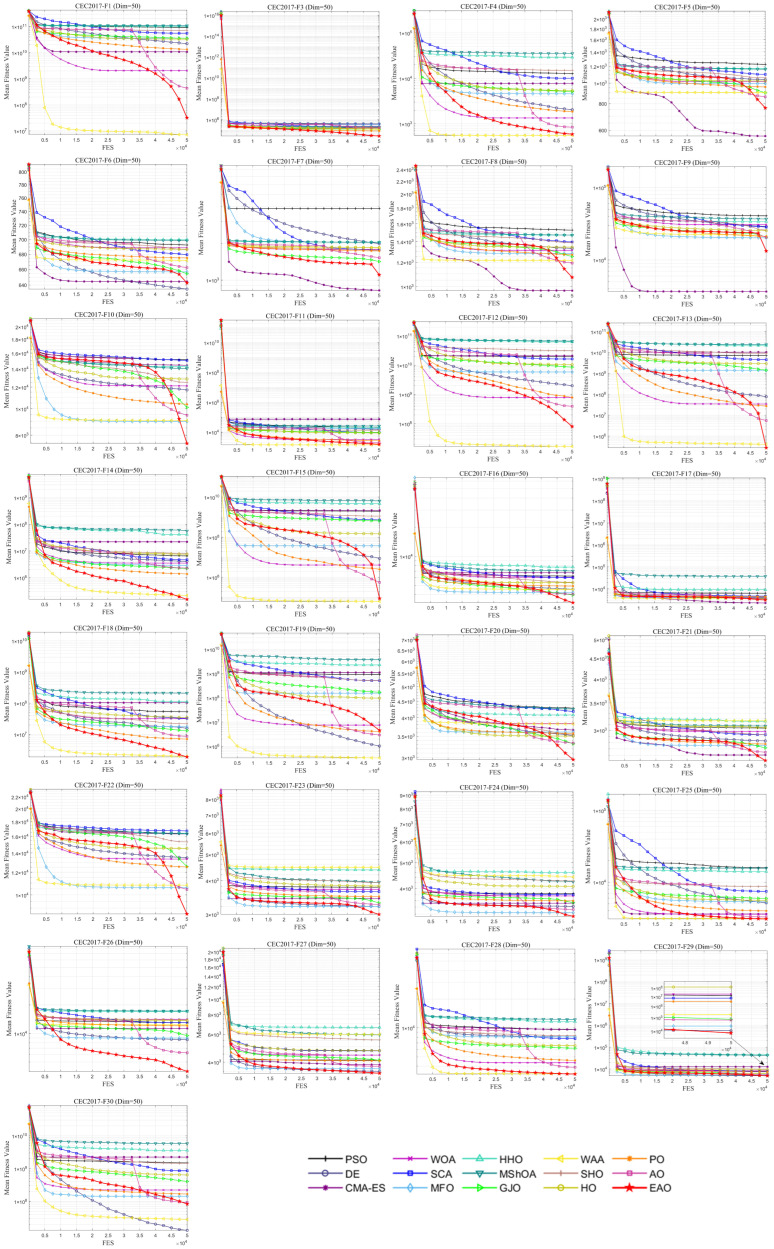
Average iteration curve of EAO and other algorithms on CEC2017 (Dim = 50).

**Figure 11:**
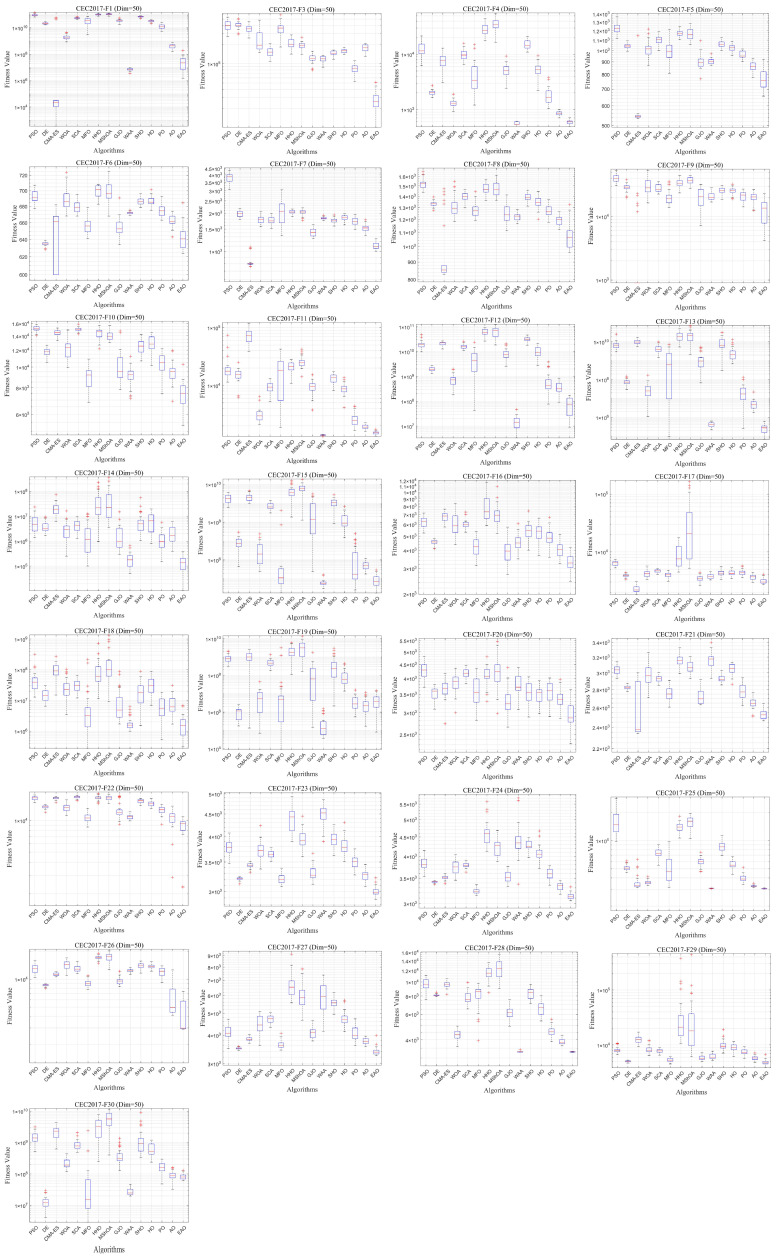
Boxplot of EAO and other algorithms on CEC2017 (Dim = 50).

**Figure 12:**
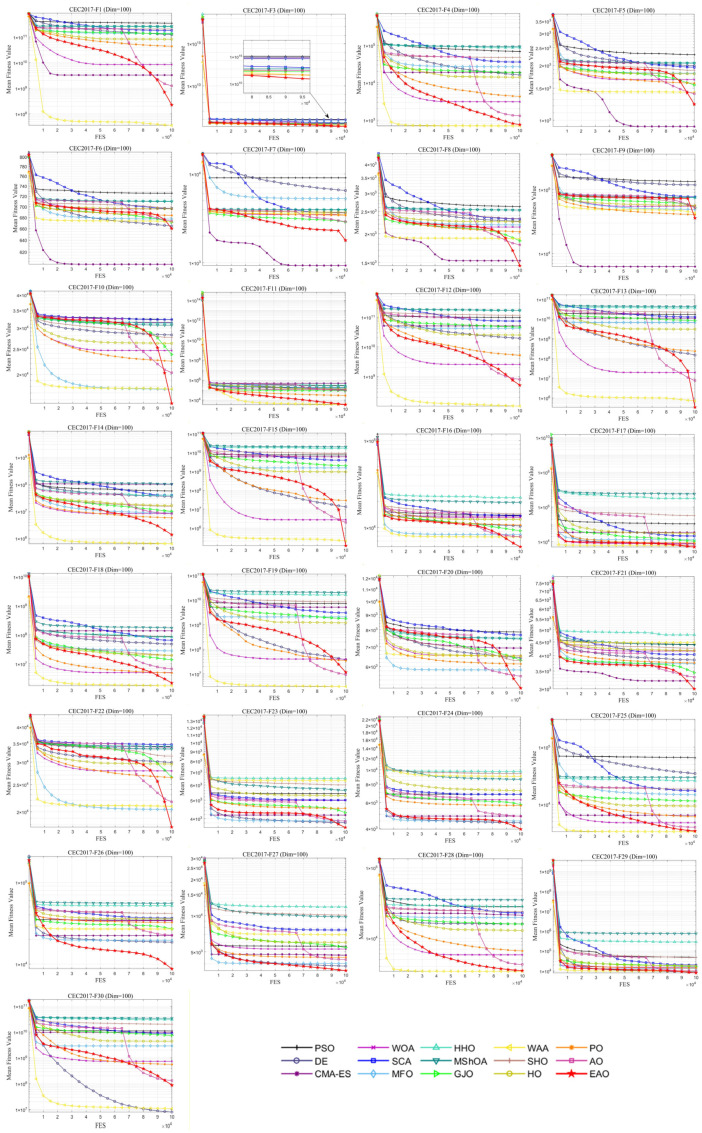
Average iteration curve of EAO and other algorithms on CEC2017 (Dim = 100).

**Figure 13:**
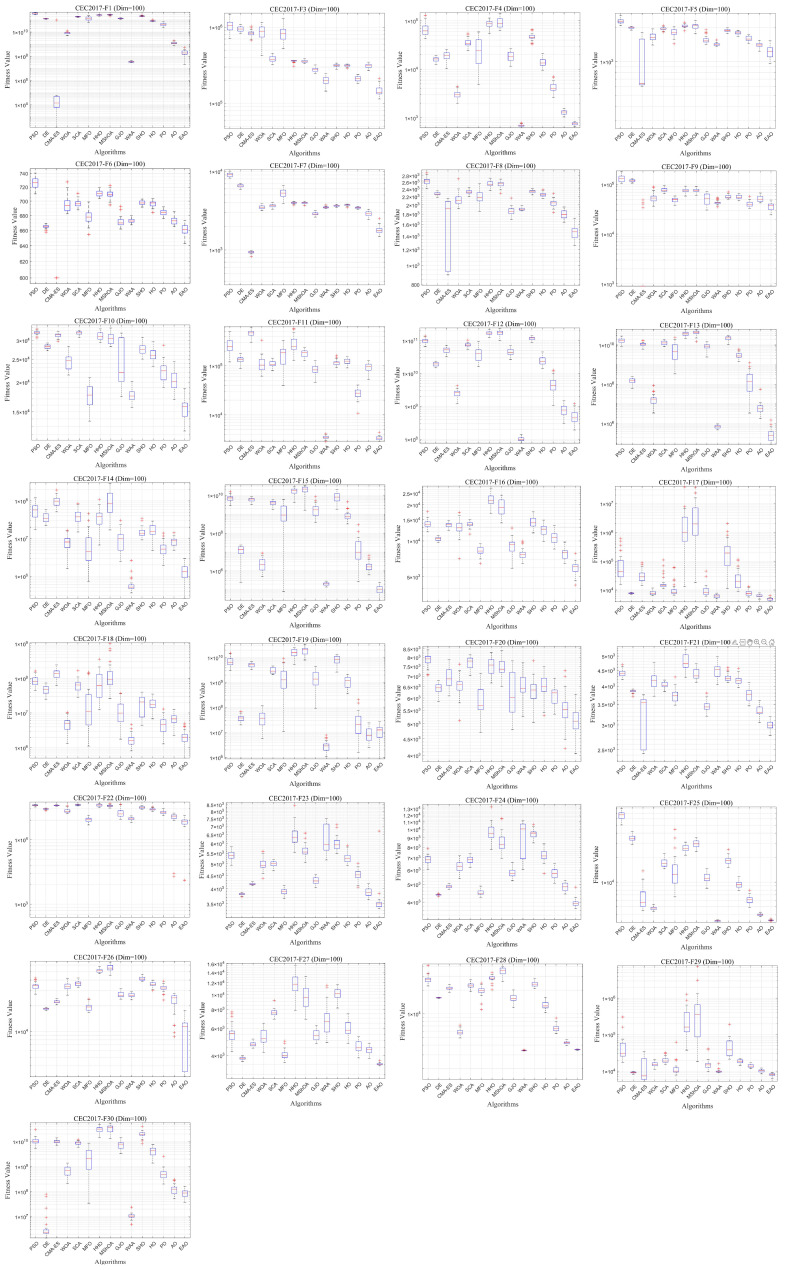
Boxplot of EAO and other algorithms on CEC2017 (Dim = 100).

Figure 7 summarizes the average ranking values of EAO and other algorithms across 30/50/100 dimensions. The convergence curves and boxplots obtained by all algorithms on each function are shown in Figure 8, Figure 9, Figure 10, Figure 11, Figure 12 and Figure 13, respectively. To enhance readability, detailed test results are presented in Table A10, Table A11, Table A12, Table A13, Table A14 and Table A15 of Appendix A.2, including the best value, mean value, variance, *p*-value, and Friedman ranking. The best values in the tables are highlighted in bold and underlined. The symbols “+/=/–” in the tables indicate that EAO performs better than, equal to, or worse than the other algorithms, respectively.

As can be seen from the data in the tables, EAO achieves the highest number of best metric values compared to the other algorithms across all dimensions. For the 30-dimensional case, EAO obtains the best value in 28 out of a total of 87 metrics across all functions, achieves the first rank in 19 functions based on the Friedman test, and has an average rank of 1.6. When the dimension is 50 or 100, EAO achieves the best value in 36 metrics and ranks first in 17 functions, with average ranks of 1.6 and 1.5, respectively. Therefore, EAO ranks first among all algorithms across all dimensions, demonstrating its ability to maintain good search accuracy and stability across different dimensions, especially in medium- and high-dimensional problems. Meanwhile, the results of the Wilcoxon rank-sum test show that EAO has a statistically significant performance advantage over most of the compared algorithms. These results fully validate the scalability and competitive advantage of EAO in medium- and high-dimensional complex optimization problems. Furthermore, the convergence curves show that EAO is able to escape local traps, even in the middle and later stages, on most functions and quickly find better solutions, indicating that the search-state-aware adaptive behavior selection framework, which integrates the four improvement strategies, effectively enhances convergence accuracy.

### 6.6. Test Results and Analysis on CEC2020

**Figure 14:**
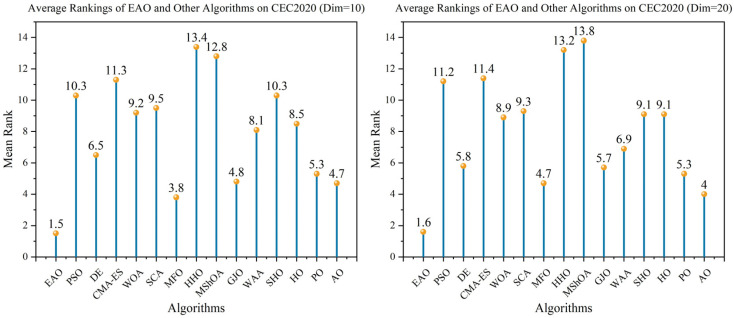
Friedman average rankings of EAO and other algorithms across different dimensions at CEC2017.

**Figure 15:**
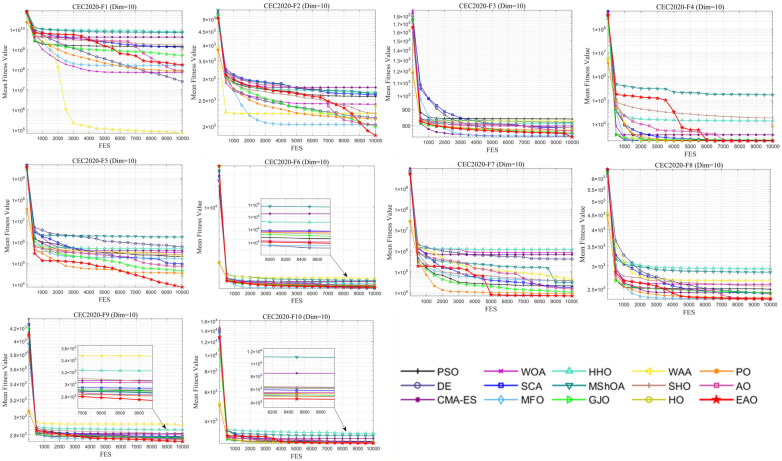
Average iteration curve of EAO and other algorithms on CEC2020 (Dim = 10).

**Figure 16:**
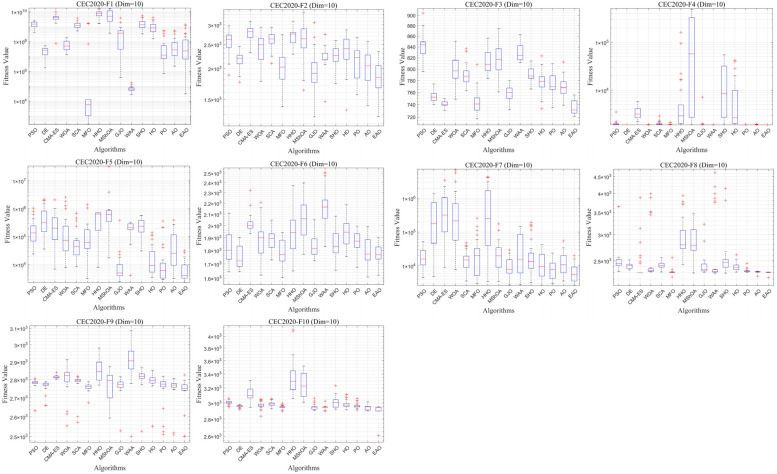
Boxplot of EAO and other algorithms on CEC2020 (Dim = 10).

**Figure 17:**
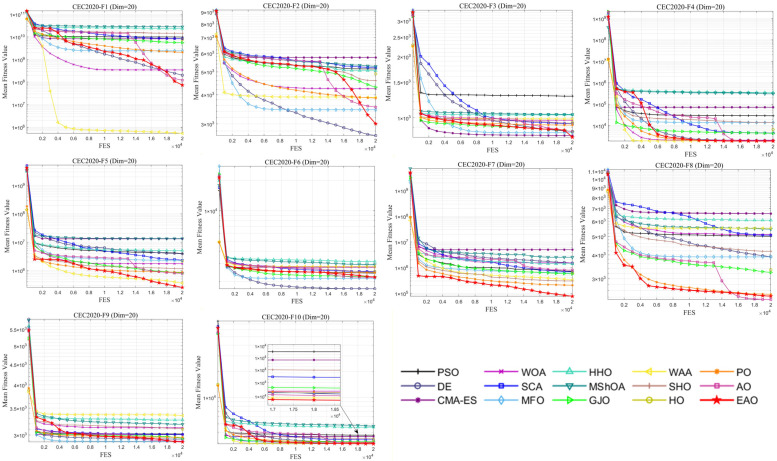
Average iteration curve of EAO and other algorithms on CEC2020 (Dim = 20).

**Figure 18:**
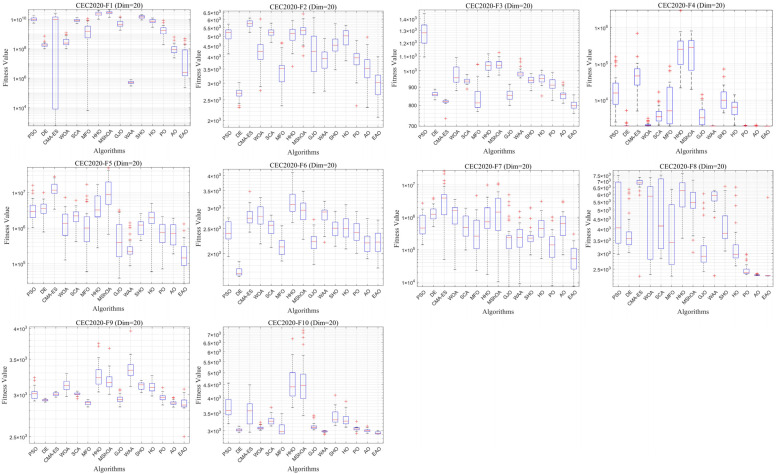
Boxplot of EAO and other algorithms on CEC2020 (Dim = 20).

In addition to medium- and high-dimensional optimization problems, the generalization ability of algorithms under different benchmark definitions and lower-dimensional conditions is also worthy of attention. Therefore, this subsection further evaluates the performance of EAO on 10-dimensional and 20-dimensional problems using the CEC2020 benchmark function set. By comparing the performance of EAO with other competing algorithms in terms of best value, mean value, and standard deviation, and then combining the Friedman ranking and Wilcoxon rank-sum test results, the robustness and generalization capability of EAO under different problem characteristics can be verified. Figure 14 summarizes the average ranking values of EAO and other algorithms across 10/20 dimensions. The convergence curves and boxplots obtained by all algorithms on each function are shown in Figure 15, Figure 16, Figure 17 and Figure 18, respectively. To enhance readability, detailed test results are presented in Table A16, Table A17, Table A18 and Table A19 of Appendix A.2, including the best value, mean value, variance, *p*-value, and Friedman ranking. The best values in the tables are highlighted in bold and underlined. The symbols “+/=/–” in the tables indicate that EAO performs better than, equal to, or worse than the other algorithms, respectively.

From the data in the tables, it can be observed that EAO achieves the highest number of best metric values compared to the other algorithms across all dimensions. For the 10-dimensional case, EAO obtains the best value in 17 out of a total of 30 metrics across all functions, achieves the first rank in 8 functions based on the Friedman test, and has an average rank of 1.5. When the dimension is 20, EAO achieves the best value in 13 metrics and ranks first in 6 functions, with an average rank of 1.6. Therefore, EAO ranks first among all algorithms across all dimensions, indicating that it also maintains good search accuracy and stability in lower-dimensional problems. Meanwhile, the results of the Wilcoxon rank-sum test show that EAO has a statistically significant performance advantage over most of the compared algorithms. Combined with the test results on CEC2017, EAO demonstrates excellent optimization performance across low-, medium-, and high-dimensional problems. This indicates that the proposed improvement strategies are not specifically designed for certain benchmark functions or dimensions but rather exhibit good adaptability and generalization ability under different problem characteristics.

## 7. Multi-UAV Cooperative Path-Planning Simulation Experiment

To validate the applicability and effectiveness of the proposed Enhanced Aquila Optimizer (EAO) in practical engineering problems, this section applies EAO to a multi-UAV cooperative path-planning simulation scenario and conducts a comparative analysis with various representative optimization algorithms. Compared to standard benchmark functions, the multi-UAV path-planning problem not only exhibits high-dimensional and nonlinear characteristics but also requires simultaneous consideration of UAV dynamic constraints, environmental threats, no-fly zone avoidance, and cooperative safety among multiple UAVs, placing higher demands on the comprehensive performance of optimization algorithms.

This section constructs several three-dimensional flight environments with varying levels of complexity, based on elevation maps. Through quantitative metrics and visual path results, a systematic evaluation of EAO’s optimization capability and cooperative performance in multi-UAV cooperative path-planning tasks is conducted.

### 7.1. Experimental Configuration for Path-Planning Simulation

**Figure 19:**
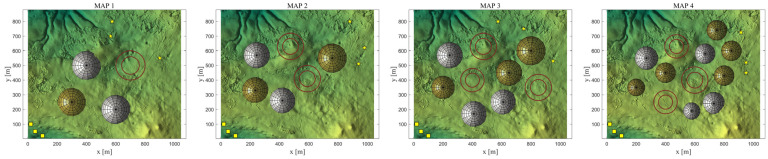
Four simulated map environments for the path-planning experiments.

The experiment constructs four typical flight scenarios of increasing complexity based on elevation maps with dimensions of 1045 × 879 × 300, as shown in Figure 19. In these scenarios, three UAVs perform cooperative path planning. The yellow square on the map indicates the starting point, the yellow star marks the endpoint, the red cylinder denotes the no-fly zone, and the yellow and white spheres represent threats such as artillery and radar, respectively. Detailed configurations of environmental parameters for different scenarios are provided in Table 5. The constraint parameter settings for the UAVs are listed in Table 6.

Regarding algorithm parameter settings, the maximum number of function evaluations is set to 10,000, and the population size is set to 30. To comprehensively validate the performance of EAO, this paper selects five representative optimization algorithms as comparison methods, including the classic algorithms PSO and SCA, the original Aquila Optimizer (AO), and the newly proposed algorithms GJO and WAA, which demonstrated relatively excellent performance in the earlier benchmark tests. All algorithms are run independently 30 times under identical parameter configurations to minimize the influence of random factors on the experimental results.

In terms of performance evaluation, in addition to comparing the overall performance of the algorithms using the best fitness value, mean value, standard deviation, and Friedman ranking of the optimization problem, the number of collision-free trajectories generated by each algorithm during the path-planning process that avoid no-fly zones and threat areas is also recorded. The average running time of each algorithm is further provided to comprehensively assess the performance of EAO in terms of path-planning quality and computational efficiency.

### 7.2. Sensitivity Analysis of Objective Function Weight Coefficients

Since the selection of objective function weights can influence the optimization results in multi-UAV path planning, sensitivity and robustness analyses are necessary. Considering that safety is of primary importance, the threat-related cost and the no-fly zone constraint cost are assigned the largest weights. In addition, the time coordination cost among multiple UAVs and the collision avoidance cost are also emphasized.

Accordingly, four key components ($\omega_{3}$–$\omega_{6}$) are treated as dominant weights and initially set to 0.18, 0.18, 0.18, and 0.14, respectively. The sensitivity experiments are conducted on the most complex multi-UAV path-planning simulation scenario (Map 4). In each experiment, only one dominant weight is varied by ±20%, while the remaining three dominant weights are kept unchanged. The weights of the other four components (path length, altitude, turning angle, and trajectory segments) are adjusted accordingly to ensure that the sum of all weight coefficients remains equal to one. This design guarantees the fairness and independence of the sensitivity evaluation.

The experimental results are reported in Table 7. When ±20% perturbations are applied to the threat cost, no-fly zone constraint cost, time coordination cost, and collision avoidance cost, the average fitness values generated by EAO exhibited variation ranges of 8.7%, 14.5%, 3.65%, and 14.11%, respectively. Although the absolute fitness values change, the convergence behavior and relative performance trends remain stable. These observations indicate that the proposed EAO algorithm does not rely on a specific weight configuration and exhibits satisfactory robustness.

Based on the above analysis, the final weight coefficients $\omega_{1}$ to $\omega_{8}$ are set to 0.10, 0.12, 0.18, 0.18, 0.18, 0.14, 0.08, and 0.02, respectively.

### 7.3. Path-Planning Simulation Results and Analysis

**Figure 20:**
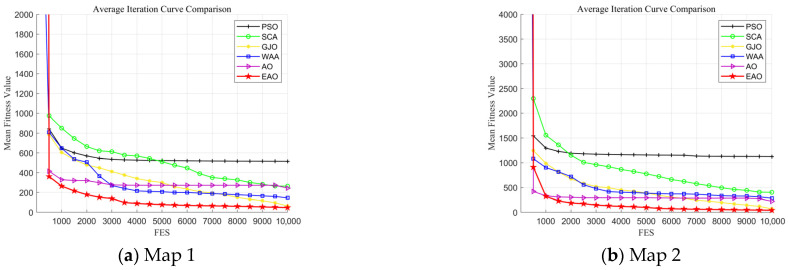

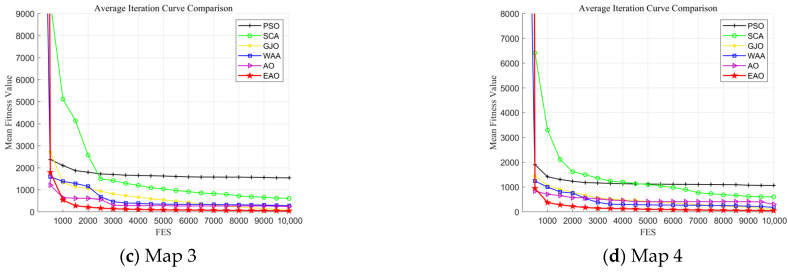
Average iteration curves of EAO and other algorithms on four maps.

**Figure 21:**
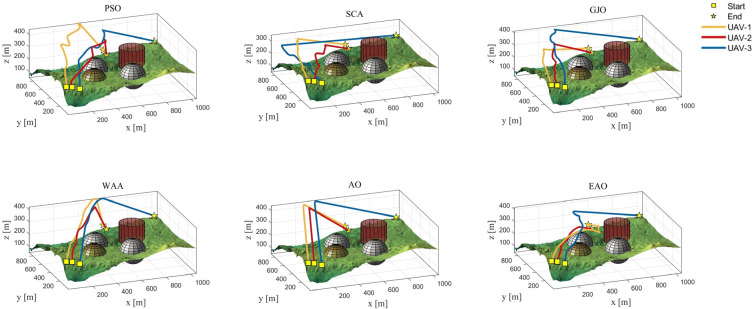
The 3D views of optimal trajectories generated by EAO and other algorithms on Map 1.

**Figure 22:**
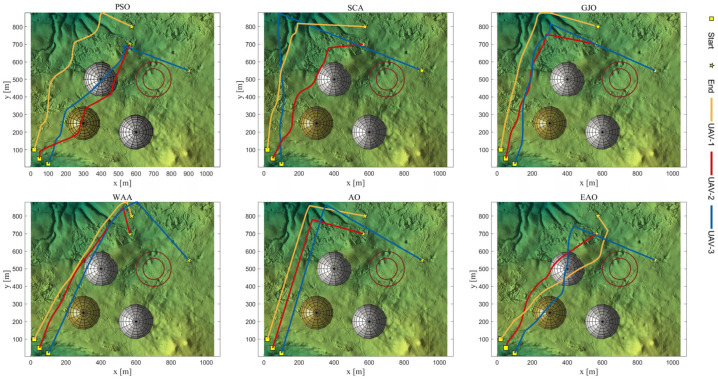
Top viewss of optimal trajectories generated by EAO and other algorithms on Map 1.

**Figure 23:**
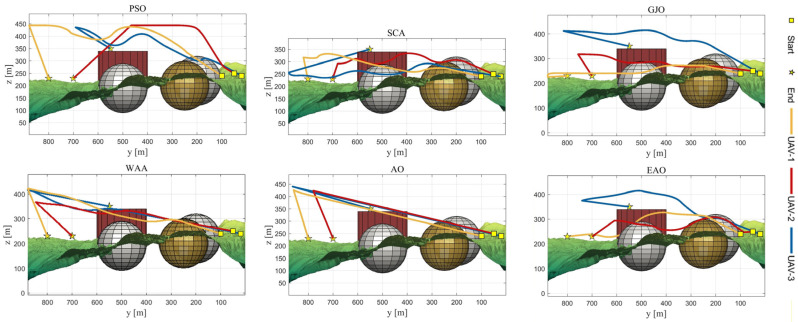
Side views of optimal trajectories generated by EAO and other algorithms on Map 1.

**Figure 24:**
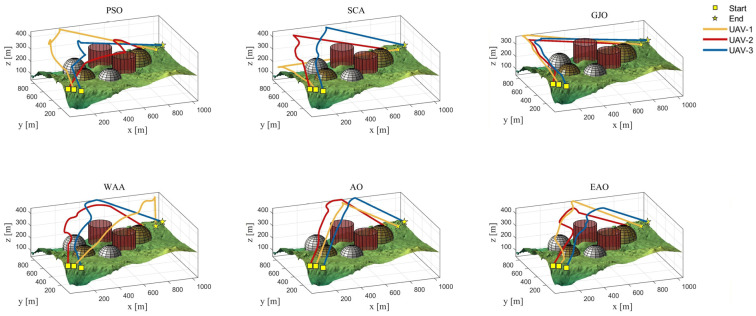
The 3D views of optimal trajectories generated by EAO and other algorithms on Map 2.

**Figure 25:**
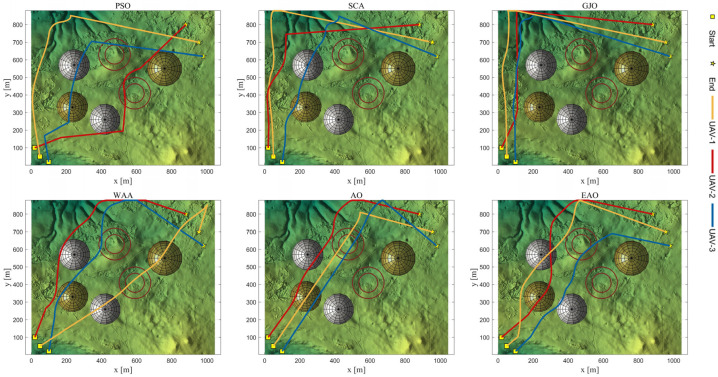
Top views of optimal trajectories generated by EAO and other algorithms on Map 2.

**Figure 26:**
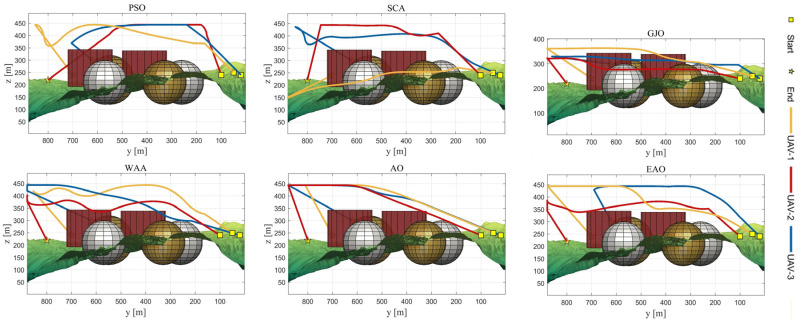
Side views of optimal trajectories generated by EAO and other algorithms on Map 2.

**Figure 27:**
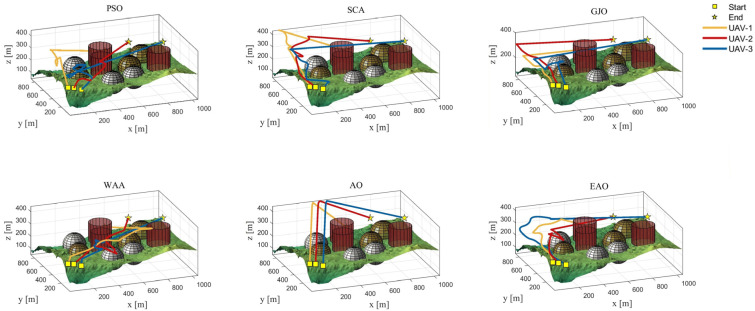
The 3D views of optimal trajectories generated by EAO and other algorithms on Map 3.

**Figure 28:**
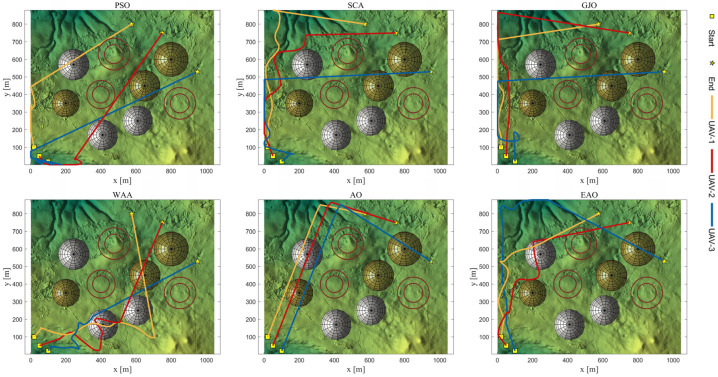
Top views of optimal trajectories generated by EAO and other algorithms on Map 3.

**Figure 29:**
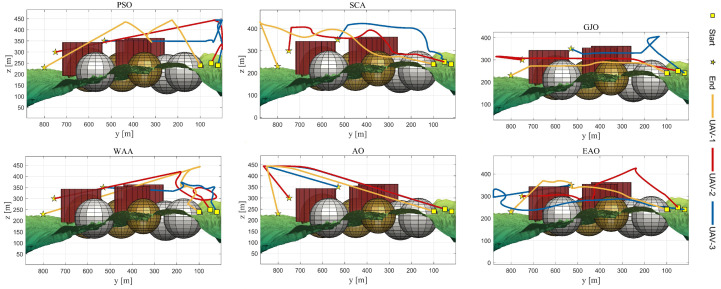
Side views of optimal trajectories generated by EAO and other algorithms on Map 3.

**Figure 30:**
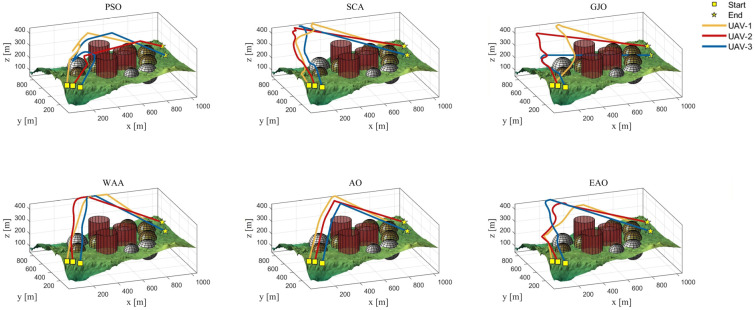
The 3D views of optimal trajectories generated by EAO and other algorithms on Map 4.

**Figure 31:**
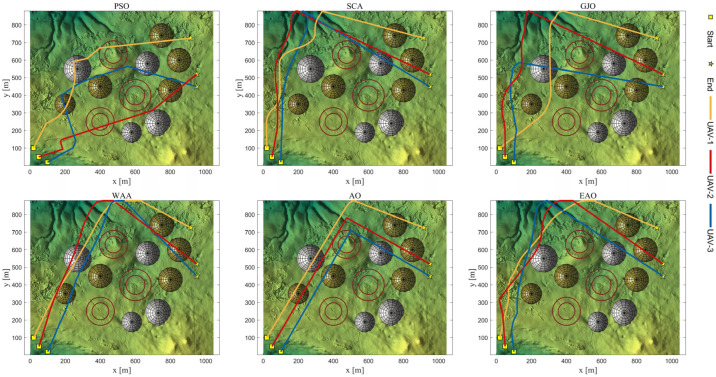
Top views of optimal trajectories generated by EAO and other algorithms on Map 4.

**Figure 32:**
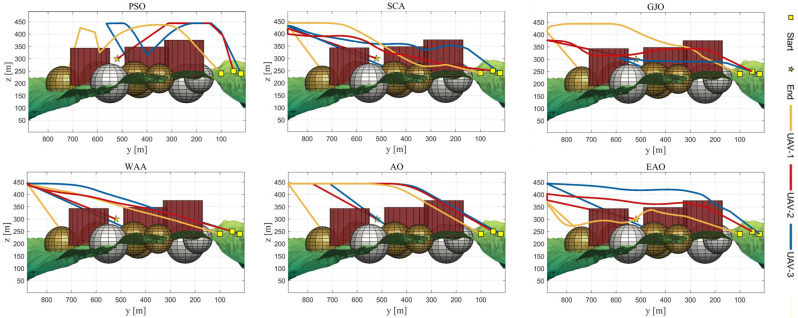
Side views of optimal trajectories generated by EAO and other algorithms on Map 4.

Table 8 presents the results of cooperative path planning for multiple UAVs across four maps using all algorithms. The best values for each metric in the table are highlighted in bold and underlined. Figure 20 shows the average convergence curves of all algorithms for path planning on the four maps, while Figure 21, Figure 22, Figure 23, Figure 24, Figure 25, Figure 26, Figure 27, Figure 28, Figure 29, Figure 30, Figure 31 and Figure 32 display the 3D main view, top view, and side view of the optimal collision-free and threat/no-fly-zone-free paths planned by each algorithm.

From the data in the table, it can be observed that in the four simulation scenarios with progressively increasing complexity, EAO achieves the best results in all fitness-related evaluation metrics, with its Friedman ranking consistently placing first. Compared to the original AO algorithm, EAO improves the average fitness values by 80.42%, 81.25%, 81.34%, and 84.84% in the four map environments, respectively, indicating that the proposed improvement strategies significantly enhance path-planning performance. In terms of the number of valid trajectories, EAO plans the highest number of valid collision-free trajectories that avoid no-fly zones and threat areas in Map 1, Map 2, and Map 4. In Map 3, although the number of valid trajectories generated by EAO is slightly lower than that of WAA, its corresponding fitness value remains superior to WAA, suggesting that EAO demonstrates better overall performance in path quality. Regarding average runtime, the differences among the six compared algorithms are minimal, indicating that EAO improves path-planning performance without significantly increasing computational overhead.

The optimal path-planning results shown in Figure 20, Figure 21, Figure 22, Figure 23, Figure 24, Figure 25, Figure 26, Figure 27, Figure 28, Figure 29, Figure 30, Figure 31 and Figure 32 further demonstrate that EAO can plan smooth flight paths from the start to the endpoint under complex constraints, avoiding collisions and traversing neither threat areas nor no-fly zones. Compared to the second-ranked GJO algorithm, EAO exhibits superior performance in both convergence speed and path quality.

In summary, EAO outperforms the compared algorithms in terms of path quality, result stability, and statistical ranking, demonstrating the best overall performance and validating its effectiveness in multi-UAV cooperative path planning under complex constraints.

## 8. Conclusions

This paper focuses on the application of Aquila Optimizer AO in complex optimization problems and multi-UAV cooperative path planning. To address the issues of insufficient diversity and limited balance between exploration and exploitation during the search process in the original AO, an enhanced AO based on an adaptive behavior selection mechanism (EAO) was designed and proposed. Its performance was validated through a series of benchmark experiments and path-planning simulation experiments.

First, to determine appropriate values for key parameters in the algorithm, parameter sensitivity experiments were conducted. Based on CEC2017 test functions, the optimization performance of EAO under different parameter combinations was compared and analyzed. By comparing statistical results such as the best, mean, and variance of function values, a parameter configuration with relatively better performance was selected, providing a unified parameter basis for subsequent experiments.

Second, ablation experiments and exploration–exploitation experiments were performed on the 30-dimensional test functions of CEC2017. In the ablation experiments, multiple algorithm variants were constructed and compared with the original AO to systematically analyze the effectiveness of each improvement strategy across different test functions. The experimental results indicate that each improvement strategy positively contributed to enhancing the algorithm’s performance, while the complete EAO demonstrated the best overall performance. In the exploration–exploitation experiments, the population dimensional diversity metric was introduced to analyze the changes in exploration and exploitation capabilities during the iterative process. The results show that EAO exhibits reasonable behavioral variations in different phases of the search process.

In terms of comprehensive performance evaluation, this paper further conducted systematic comparative tests between the proposed algorithm and 14 other representative competing algorithms on the CEC2017 and CEC2020 benchmark suites. Specifically, the CEC2017 test set was used under 30-, 50-, and 100-dimensional conditions to evaluate the algorithm’s performance in medium- to high-dimensional optimization problems, while the CEC2020 test set was employed under 10- and 20-dimensional conditions to examine its optimization performance in lower-dimensional problems. The experimental results were analyzed using statistical metrics such as the best, mean, and variance values, along with the Friedman ranking and Wilcoxon rank-sum test. The results, when compared, demonstrate that EAO achieved stable optimization results across most test functions and different dimensional conditions, securing the top overall ranking.

Finally, the proposed EAO and five other competing algorithms were applied to multi-UAV cooperative path-planning simulation experiments. Multiple experimental environments, ranging from simple to complex, were constructed based on elevation maps, and factors such as no-fly zones, threat areas, and UAV dynamic constraints were comprehensively considered to simulate the multi-UAV cooperative path-planning process. Comparative analysis of metrics such as path-planning fitness, number of valid trajectories, and average runtime shows that EAO ranks first among the six algorithms. Compared to the original AO algorithm, EAO improved the average fitness values by 80.42%, 81.25%, 81.34%, and 84.84% across different map environments, confirming its feasibility and effectiveness in multi-UAV path-planning problems.

From an application perspective, the path-planning method based on the adaptive behavior mechanism proposed in this paper is not only suitable for multi-UAV cooperative path planning but can also be extended to other complex optimization and planning tasks characterized by high dimensionality, strong nonlinearity, and multiple constraints. Examples include mobile robot path planning, multi-robot cooperative search and coverage, unmanned vehicle path planning, and parameter optimization in complex engineering systems. Due to its low dependency on problem modeling and good generality and scalability, EAO holds potential for application in related fields of intelligent optimization and autonomous decision-making.

Although the improved AO proposed in this paper has achieved certain success in multi-UAV path planning, the current research is primarily based on the assumption of a static environment and does not fully account for factors such as dynamic threats and real-time task changes. Therefore, future work will explore the following directions to extend this study: First, the proposed behavior-adaptive AO framework will be extended to dynamic environments, where time-varying threats, moving obstacles, and real-time task changes are considered, enabling online path replanning for multi-UAV systems. Second, more complex cooperative scenarios will be investigated, including communication constraints, formation maintenance, and dynamic task allocation among multiple UAVs. Finally, the proposed method will be validated in larger-scale and more realistic environments to further evaluate its scalability and practical applicability.

## Figures and Tables

**Table 1:** Description of symbols used in AO.

Symbols	Description
N	Population size.
T	Maximum number of iterations.
t	Current iteration number.
UB	Upper bounds of the problem.
LB	Lower bounds of the problem.
Dim	Problem dimension.
D	Dimension space.
Levy(D)	Flight distribution function.
rand, σ, ζ	Random numbers between [0, 1].
X(t)	Current solution at the tth iteration.
$X_{best}\left(t\right)$	Best solution before the tth iteration
$X_{M}(t)$	Mean of the current solutions at the tth iteration.
$QF\left(t\right)$	Quality function used to balance the search strategies.
$G_{1}$	Various movements of the eagle used to track the prey during its escape.
$G_{2}$	Flight slope of the eagle tracking the prey from the first to the last position during the prey’s evasion.

**Table 2:** Description of symbols used in EAO.

Symbols	Description
D(t)	Population diversity index.
$\hat{D}\left(t\right)$	Normalized population diversity index.
$X_{i}(t)$	Position vector of the ith individual at generation t.
$\bar{X}(t)$	Center position of the population.
$X_{lbest}\left(t\right)$	Neighborhood best position of the individual.
$k_{min}$	Minimum neighborhood size.
$k_{max}$	Maximum neighborhood size
μ	$k_{max}$ as a percentage of the population size.
$\theta_{L}$	Low diversity threshold.
$\theta_{H}$	High diversity threshold.
F	Scaling factor.
τ	Historical window length.
η	Time-varying amplitude decay coefficient.
$f_{best}(t)$	Global optimal fitness value of the tth generation.
$f_{i}(t)$	Fitness value of the ith individual in generation t.
$M_{i}^{s}\left(t\right)$	Suggested behavior number.
$M_{i}\left(t\right)$	Behavior number currently executed.
${TR}_{i}\left(t\right)$	Behavior trust.
${TR}_{min}$	Lower limit of trust.
${TR}_{max}$	Upper limit of trust.
$S_{i}\left(t\right)$	Consecutive number of times this behavior has produced improvements.

**Table 3:** Algorithm parameter settings.

Algorithms	Parameter Settings
PSO	$C_{1}=1,C_{2}=1,v_{min}=-10,v_{max}=10,\omega =[0.9,0.4]$
DE	F=0.5, CR=0.5
CMA-ES	σ=0.75
WOA	a=[2,0]
SCA	a=2
MFO	Parameter free
HHO	a=5,β=1.5
MShOA	b=1,φ=10
GJO	Parameter free
WAA	Parameter free
SHO	u=0.05,v=0.05;l=0.05
HO	Parameter free
PO	β=0.5
AO	$\alpha =0.1,\delta =0.1,r_{1}=10$

**Table 4:** Ablation experiment results.

Function	Index	AO	EAO_D	EAO_N	EAO_E	EAO_S	EAO
F1	Best	7.519 × 10^7^	4.259 × 10^7^	2.240 × 10^9^	9.716 × 10^9^	**2.751 × 10^5^**	3.647 × 10^5^
	Mean	2.172 × 10^8^	2.133 × 10^8^	4.312 × 10^9^	1.756 × 10^10^	**1.842 × 10^6^**	1.702 × 10^7^
	Std	9.657 × 10^7^	1.412 × 10^8^	1.268 × 10^9^	4.962 × 10^9^	**1.353 × 10^6^**	5.074 × 10^7^
	Rank	4	3	5	6	1	2
F3	Best	3.907 × 10^4^	3.917 × 10^4^	6.006 × 10^4^	3.475 × 10^4^	**1.546 × 10^3^**	1.606 × 10^3^
	Mean	5.219 × 10^4^	5.418 × 10^4^	7.509 × 10^4^	6.189 × 10^4^	4.910 × 10^3^	**4.130 × 10^3^**
	Std	6.975 × 10^3^	6.798 × 10^3^	7.056 × 10^3^	1.125 × 10^4^	2.554 × 10^3^	**1.504 × 10^3^**
	Rank	3	4	6	5	2	1
F4	Best	5.510 × 10^2^	5.363 × 10^2^	6.881 × 10^2^	8.796 × 10^2^	4.728 × 10^2^	**4.493 × 10^2^**
	Mean	6.196 × 10^2^	6.101 × 10^2^	8.880 × 10^2^	1.796 × 10^3^	**4.967 × 10^2^**	5.035 × 10^2^
	Std	5.675 × 10^1^	4.759 × 10^1^	1.447 × 10^2^	6.840 × 10^2^	**1.244 × 10^1^**	2.356 × 10^1^
	Rank	4	3	5	6	1	2
F5	Best	6.263 × 10^2^	6.295 × 10^2^	6.451 × 10^2^	6.882 × 10^2^	5.673 × 10^2^	**5.671 × 10^2^**
	Mean	6.778 × 10^2^	6.892 × 10^2^	6.941 × 10^2^	7.587 × 10^2^	6.316 × 10^2^	**6.315 × 10^2^**
	Std	3.001 × 10^1^	3.362 × 10^1^	**2.265 × 10^1^**	4.106 × 10^1^	4.588 × 10^1^	4.337 × 10^1^
	Rank	3	4	5	6	2	1
F6	Best	6.317 × 10^2^	6.367 × 10^2^	6.288 × 10^2^	6.198 × 10^2^	6.069 × 10^2^	**6.064 × 10^2^**
	Mean	6.469 × 10^2^	6.488 × 10^2^	6.423 × 10^2^	6.412 × 10^2^	**6.276 × 10^2^**	6.331 × 10^2^
	Std	1.038 × 10^1^	7.041 × 10^0^	**5.897 × 10^0^**	1.597 × 10^1^	1.170 × 10^1^	1.495 × 10^1^
	Rank	5	6	4	3	1	2
F7	Best	1.021 × 10^3^	1.005 × 10^3^	1.044 × 10^3^	1.009 × 10^3^	**7.865 × 10^2^**	8.311 × 10^2^
	Mean	1.099 × 10^3^	1.083 × 10^3^	1.099 × 10^3^	1.131 × 10^3^	**8.748 × 10^2^**	8.797 × 10^2^
	Std	5.071 × 10^1^	5.475 × 10^1^	**4.114 × 10^1^**	8.108 × 10^1^	4.521 × 10^1^	4.136 × 10^1^
	Rank	5	3	4	6	1	2
F8	Best	9.122 × 10^2^	9.141 × 10^2^	9.274 × 10^2^	9.641 × 10^2^	**8.491 × 10^2^**	8.599 × 10^2^
	Mean	9.518 × 10^2^	9.596 × 10^2^	9.731 × 10^2^	1.039 × 10^3^	**9.273 × 10^2^**	9.279 × 10^2^
	Std	3.088 × 10^1^	2.557 × 10^1^	**2.107 × 10^1^**	3.883 × 10^1^	3.767 × 10^1^	4.658 × 10^1^
	Rank	3	4	5	6	2	1
F9	Best	4.036 × 10^3^	4.013 × 10^3^	4.509 × 10^3^	2.881 × 10^3^	1.057 × 10^3^	**9.851 × 10^2^**
	Mean	6.662 × 10^3^	6.398 × 10^3^	6.342 × 10^3^	6.335 × 10^3^	**3.193 × 10^3^**	3.472 × 10^3^
	Std	1.308 × 10^3^	1.182 × 10^3^	**1.159 × 10^3^**	2.049 × 10^3^	2.024 × 10^3^	1.736 × 10^3^
	Rank	5	6	3	4	1	2
F10	Best	4.644 × 10^3^	4.211 × 10^3^	4.606 × 10^3^	6.021 × 10^3^	4.041 × 10^3^	**3.071 × 10^3^**
	Mean	5.623 × 10^3^	5.666 × 10^3^	5.694 × 10^3^	8.449 × 10^3^	5.105 × 10^3^	**4.578 × 10^3^**
	Std	6.753 × 10^2^	8.547 × 10^2^	**4.279 × 10^2^**	6.916 × 10^2^	5.856 × 10^2^	6.361 × 10^2^
	Rank	3	4	5	6	2	1
F11	Best	1.585 × 10^3^	1.499 × 10^3^	1.859 × 10^3^	1.491 × 10^3^	1.188 × 10^3^	**1.160 × 10^3^**
	Mean	1.869 × 10^3^	1.870 × 10^3^	4.311 × 10^3^	2.715 × 10^3^	1.313 × 10^3^	**1.301 × 10^3^**
	Std	2.309 × 10^2^	2.778 × 10^2^	1.424 × 10^3^	1.155 × 10^3^	**5.955 × 10^1^**	6.252 × 10^1^
	Rank	4	3	6	5	2	1
F12	Best	6.299 × 10^6^	7.772 × 10^6^	5.127 × 10^7^	2.486 × 10^8^	2.060 × 10^6^	**1.195 × 10^6^**
	Mean	6.142 × 10^7^	4.960 × 10^7^	2.132 × 10^8^	1.529 × 10^9^	2.017 × 10^7^	**1.888 × 10^7^**
	Std	4.329 × 10^7^	3.824 × 10^7^	1.048 × 10^8^	1.317 × 10^9^	**2.033 × 10^7^**	2.220 × 10^7^
	Rank	4	3	5	6	2	1
F13	Best	2.540 × 10^5^	2.216 × 10^5^	8.888 × 10^5^	1.211 × 10^5^	5.788 × 10^4^	**4.509 × 10^4^**
	Mean	1.026 × 10^6^	1.144 × 10^6^	8.252 × 10^6^	2.670 × 10^8^	1.877 × 10^5^	**1.259 × 10^5^**
	Std	1.134 × 10^6^	1.141 × 10^6^	6.855 × 10^6^	5.123 × 10^8^	1.218 × 10^5^	**6.235 × 10^4^**
	Rank	4	3	5	6	2	1
F14	Best	2.150 × 10^4^	4.258 × 10^4^	7.883 × 10^4^	**1.674 × 10^3^**	5.133 × 10^3^	2.645 × 10^3^
	Mean	6.234 × 10^5^	3.648 × 10^5^	5.870 × 10^5^	4.535 × 10^4^	3.759 × 10^4^	**2.785 × 10^4^**
	Std	6.387 × 10^5^	4.998 × 10^5^	6.280 × 10^5^	4.630 × 10^4^	3.503 × 10^4^	**2.527 × 10^4^**
	Rank	6	4	5	3	2	1
F15	Best	4.637 × 10^4^	4.579 × 10^4^	2.378 × 10^4^	**1.056 × 10^4^**	1.165 × 10^4^	1.145 × 10^4^
	Mean	1.499 × 10^5^	1.281 × 10^5^	1.413 × 10^5^	6.593 × 10^6^	6.649 × 10^4^	**4.896 × 10^4^**
	Std	8.379 × 10^4^	6.827 × 10^4^	8.395 × 10^4^	2.026 × 10^7^	5.726 × 10^4^	**2.459 × 10^4^**
	Rank	6	4	5	3	2	1
F16	Best	2.637 × 10^3^	2.262 × 10^3^	2.317 × 10^3^	2.627 × 10^3^	2.160 × 10^3^	**1.989 × 10^3^**
	Mean	3.145 × 10^3^	3.118 × 10^3^	3.048 × 10^3^	3.286 × 10^3^	2.829 × 10^3^	**2.815 × 10^3^**
	Std	3.584 × 10^2^	3.060 × 10^2^	**2.808 × 10^2^**	3.470 × 10^2^	3.237 × 10^2^	3.551 × 10^2^
	Rank	5	4	3	6	1	2
F17	Best	1.899 × 10^3^	2.007 × 10^3^	1.900 × 10^3^	1.893 × 10^3^	**1.860 × 10^3^**	1.886 × 10^3^
	Mean	2.307 × 10^3^	2.315 × 10^3^	2.194 × 10^3^	2.247 × 10^3^	**2.166 × 10^3^**	2.174 × 10^3^
	Std	2.536 × 10^2^	2.024 × 10^2^	2.050 × 10^2^	2.480 × 10^2^	**1.873 × 10^2^**	1.993 × 10^2^
	Rank	5	6	2	4	3	1
F18	Best	1.355 × 10^5^	9.962 × 10^4^	7.056 × 10^5^	3.996 × 10^4^	**1.865 × 10^4^**	6.378 × 10^4^
	Mean	3.424 × 10^6^	2.585 × 10^6^	3.377 × 10^6^	1.804 × 10^6^	4.735 × 10^5^	**3.890 × 10^5^**
	Std	3.489 × 10^6^	2.268 × 10^6^	2.906 × 10^6^	4.582 × 10^6^	3.586 × 10^5^	**2.750 × 10^5^**
	Rank	5	4	6	3	2	1
F19	Best	6.609 × 10^4^	4.219 × 10^4^	2.934 × 10^4^	**1.556 × 10^4^**	2.118 × 10^4^	1.985 × 10^4^
	Mean	2.631 × 10^6^	**2.187 × 10^6^**	2.717 × 10^6^	1.101 × 10^7^	2.552 × 10^6^	2.850 × 10^6^
	Std	2.128 × 10^6^	**1.521 × 10^6^**	2.200 × 10^6^	2.192 × 10^7^	1.926 × 10^6^	1.837 × 10^6^
	Rank	2	1	4	5	3	6
F20	Best	2.225 × 10^3^	2.330 × 10^3^	2.300 × 10^3^	**2.207 × 10^3^**	2.247 × 10^3^	2.238 × 10^3^
	Mean	2.566 × 10^3^	2.559 × 10^3^	**2.516 × 10^3^**	2.650 × 10^3^	2.525 × 10^3^	2.517 × 10^3^
	Std	1.952 × 10^2^	1.527 × 10^2^	**1.318 × 10^2^**	1.944 × 10^2^	1.969 × 10^2^	1.996 × 10^2^
	Rank	5	4	2	6	3	1
F21	Best	2.418 × 10^3^	2.410 × 10^3^	2.423 × 10^3^	2.456 × 10^3^	2.368 × 10^3^	**2.366 × 10^3^**
	Mean	2.465 × 10^3^	2.466 × 10^3^	2.473 × 10^3^	2.502 × 10^3^	2.429 × 10^3^	**2.417 × 10^3^**
	Std	2.627 × 10^1^	3.853 × 10^1^	**2.528 × 10^1^**	3.809 × 10^1^	3.885 × 10^1^	4.634 × 10^1^
	Rank	4	3	5	6	2	1
F22	Best	2.347 × 10^3^	2.360 × 10^3^	2.713 × 10^3^	3.112 × 10^3^	**2.306 × 10^3^**	2.307 × 10^3^
	Mean	2.582 × 10^3^	**2.418 × 10^3^**	3.031 × 10^3^	4.256 × 10^3^	4.632 × 10^3^	2.698 × 10^3^
	Std	8.254 × 10^2^	**4.612 × 10^1^**	2.324 × 10^2^	1.290 × 10^3^	2.046 × 10^3^	1.172 × 10^3^
	Rank	3	2	5	6	4	1
F23	Best	2.798 × 10^3^	2.774 × 10^3^	2.805 × 10^3^	2.792 × 10^3^	2.713 × 10^3^	**2.695 × 10^3^**
	Mean	2.877 × 10^3^	2.878 × 10^3^	2.878 × 10^3^	2.849 × 10^3^	**2.759 × 10^3^**	2.778 × 10^3^
	Std	4.501 × 10^1^	4.319 × 10^1^	3.427 × 10^1^	3.522 × 10^1^	**2.886 × 10^1^**	6.839 × 10^1^
	Rank	4	5	6	3	1	2
F24	Best	2.911 × 10^3^	2.926 × 10^3^	2.981 × 10^3^	2.944 × 10^3^	**2.865 × 10^3^**	2.880 × 10^3^
	Mean	3.029 × 10^3^	3.015 × 10^3^	3.035 × 10^3^	3.010 × 10^3^	2.936 × 10^3^	**2.922 × 10^3^**
	Std	5.794 × 10^1^	4.229 × 10^1^	2.696 × 10^1^	3.649 × 10^1^	7.149 × 10^1^	**2.388 × 10^1^**
	Rank	5	4	6	3	2	1
F25	Best	2.904 × 10^3^	2.917 × 10^3^	2.998 × 10^3^	3.131 × 10^3^	2.887 × 10^3^	**2.885 × 10^3^**
	Mean	2.978 × 10^3^	2.959 × 10^3^	3.107 × 10^3^	3.491 × 10^3^	**2.899 × 10^3^**	2.904 × 10^3^
	Std	2.782 × 10^1^	2.973 × 10^1^	5.936 × 10^1^	2.697 × 10^2^	1.871 × 10^1^	**1.758 × 10^1^**
	Rank	4	3	5	6	1	2
F26	Best	3.623 × 10^3^	3.569 × 10^3^	4.181 × 10^3^	4.078 × 10^3^	2.902 × 10^3^	**2.825 × 10^3^**
	Mean	4.550 × 10^3^	4.553 × 10^3^	4.925 × 10^3^	5.903 × 10^3^	4.488 × 10^3^	**3.903 × 10^3^**
	Std	1.171 × 10^3^	1.147 × 10^3^	**7.657 × 10^2^**	9.647 × 10^2^	8.597 × 10^2^	9.861 × 10^2^
	Rank	3	2	5	6	4	1
F27	Best	3.248 × 10^3^	3.229 × 10^3^	3.265 × 10^3^	3.271 × 10^3^	3.207 × 10^3^	**3.192 × 10^3^**
	Mean	3.308 × 10^3^	3.306 × 10^3^	3.330 × 10^3^	3.316 × 10^3^	**3.235 × 10^3^**	3.247 × 10^3^
	Std	3.397 × 10^1^	3.326 × 10^1^	**2.894 × 10^1^**	3.151 × 10^1^	3.259 × 10^1^	3.908 × 10^1^
	Rank	3	4	6	5	1	2
F28	Best	3.329 × 10^3^	3.302 × 10^3^	3.409 × 10^3^	3.682 × 10^3^	3.199 × 10^3^	**3.197 × 10^3^**
	Mean	3.400 × 10^3^	3.385 × 10^3^	3.777 × 10^3^	4.260 × 10^3^	**3.237 × 10^3^**	3.244 × 10^3^
	Std	5.053 × 10^1^	4.415 × 10^1^	1.873 × 10^2^	3.595 × 10^2^	**2.456 × 10^1^**	2.507 × 10^1^
	Rank	4	3	5	6	1	2
F29	Best	3.890 × 10^3^	3.843 × 10^3^	4.018 × 10^3^	3.734 × 10^3^	**3.514 × 10^3^**	3.579 × 10^3^
	Mean	4.422 × 10^3^	4.392 × 10^3^	4.470 × 10^3^	4.217 × 10^3^	3.954 × 10^3^	**3.916 × 10^3^**
	Std	4.422 × 10^3^	4.392 × 10^3^	4.470 × 10^3^	4.217 × 10^3^	3.954 × 10^3^	**3.916 × 10^3^**
	Rank	5	4	6	3	2	1
F30	Best	1.316 × 10^6^	2.001 × 10^6^	4.746 × 10^6^	3.913 × 10^6^	**5.542 × 10^5^**	1.134 × 10^6^
	Mean	1.639 × 10^7^	1.468 × 10^7^	2.429 × 10^7^	4.059 × 10^7^	**7.612 × 10^6^**	7.732 × 10^6^
	Std	1.285 × 10^7^	9.590 × 10^6^	1.846 × 10^7^	5.063 × 10^7^	6.113 × 10^6^	**5.992 × 10^6^**
	Rank	4	3	5	6	2	1
Mean Rank		4.1	3.7	4.8	5	1.9	**1.5**
Final Ranking		4	3	5	6	2	1

**Table 5:** Simulation environment parameter settings for multi-UAV path planning.

Map	Starting Point	Target Point	Threat				No-Fly Zone	
			Radar		Artillery			
			Center	R	Center	R	Center	R
1	(20, 100, 240) (50, 50, 250) (100, 20, 240)	(545, 800, 230) (565, 700, 230) (900, 550, 350)	(400, 500, 190) (600, 200, 220)	100 100	(300, 250, 200)	100	(700, 500, 200)	100
2	(20, 100, 240) (50, 50, 250) (100, 20, 240)	(880, 800, 220) (940, 510, 240) (980, 620, 300)	(420, 260, 210) (245, 570, 210)	90 90	(760, 550, 220) (235, 330, 210)	100 90	(590, 410, 200) (475, 630, 200)	90 90
3	(20, 100, 240) (50, 50, 250) (100, 20, 240)	(575, 800, 230) (750, 750, 300) (950, 530, 350)	(610, 250,200) (410, 170,210) (245, 570,210)	90 90 90	(800, 600, 220) (650, 450, 210) (200, 350, 220)	100 90 80	(850, 350, 150) (475, 630, 200) (400, 400, 200)	90 90 80
4	(20, 100, 240) (50, 50, 250) (100, 20, 240)	(915, 725, 230) (950, 520, 300) (950, 450, 250)	(670, 580, 210) (580, 190, 220) (270, 550, 200)	70 60 80	(850, 600, 220) (750, 740, 200) (400, 450, 220) (200, 350, 220)	70 70 70 60	(475, 630, 200) (400, 250, 200)	80 80

**Table 6:** Settings of constraint parameters for UAV dynamics.

Constraint Parameter	Symbol	Value
Minimum flight speed	$v_{min}$	102 m/s
Maximum flight speed	$v_{max}$	238 m/s
Range of yaw angle variation	∆ϕ	±60°
Range of pitch angle variation	∆ϑ	±45°
Minimum flight segment length	$L_{min}$	2 km
Minimum distance between UAVs	$d_{s}$	25 km

**Table 7:** Sensitivity analysis results of weight coefficient configuration for multi-UAV path-planning objective function on Map 4.

Configuration	Weight Coefficients Values of Threat Cost, No-Fly Zone Constraint Cost, Time Coordination Cost, Collision Avoidance Cost	Average Fitness Value	Average Fitness Value Changes	Fitness Value Fluctuation Percentage
Benchmark Weight	$\omega_{1}=0.1,\;{\;\omega }_{2}=0.12,\;{\;\omega }_{3}=0.18,\;{\;\omega }_{4}=0.18,$ $\omega_{5}=0.18,\;{\;\omega }_{6}=0.14,\;{\;\omega }_{7}=0.08,\;{\;\omega }_{8}=0.02$	4.627 × 10^1^	~	~
$\omega_{3}+20\%$	$\omega_{1}=0.09,\;{\;\omega }_{2}=0.113,\;{\;\omega }_{3}=0.216,{\;\omega }_{4}=0.18,\;$ $\omega_{5}=0.18,{\;\omega }_{6}=0.14,\;{\;\omega }_{7}=0.061,\;{\;\omega }_{8}=0.02$	4.282 × 10^1^	−7.46%	8.7%
$\omega_{3}-20\%$	$\omega_{1}=0.12,\;{\;\omega }_{2}=0.126,\;{\;\omega }_{3}=0.144,\;{\;\omega }_{4}=0.18,$ $\omega_{5}=0.18,\;{\;\omega }_{6}=0.14,\;{\;\omega }_{7}=0.09,\;{\;\omega }_{8}=0.02$	4.685 × 10^1^	−1.24%	
$\omega_{4}+20\%$	$\omega_{1}=0.09,\;{\;\omega }_{2}=0.113,\;{\;\omega }_{3}=0.18,{\;\omega }_{4}=0.216,\;$ $\omega_{5}=0.18,\;{\;\omega }_{6}=0.14,\;{\;\omega }_{7}=0.061,\;{\;\omega }_{8}=0.02$	4.021 × 10^1^	−13.11%	14.5%
$\omega_{4}-20\%$	$\omega_{1}=0.12,\;{\;\omega }_{2}=0.126,\;{\;\omega }_{3}=0.18,{\;\omega }_{4}=0.144,\;$ $\omega_{5}=0.18,\;{\;\omega }_{6}=0.14,\;{\;\omega }_{7}=0.09,\;{\;\omega }_{8}=0.02$	4.691 × 10^1^	1.39%	
$\omega_{5}+20\%$	$\omega_{1}=0.09,\;{\;\omega }_{2}=0.113,\;{\;\omega }_{3}=0.18,\;{\;\omega }_{4}=0.18$ $\omega_{5}=0.216,\;{\;\omega }_{6}=0.14,\;{\;\omega }_{7}=0.061,\;{\;\omega }_{8}=0.02$	4.553 × 10^1^	−1.61%	3.65%
$\omega_{5}-20\%$	$\omega_{1}=0.12,\;{\;\omega }_{2}=0.126,\;{\;\omega }_{3}=0.18,{\;\omega }_{4}=0.18,\;$ $\omega_{5}=0.144,\;{\;\omega }_{6}=0.14,\;{\;\omega }_{7}=0.09,\;{\;\omega }_{8}=0.02$	4.533 × 10^1^	−2.04%	
$\omega_{6}+20\%$	$\omega_{1}=0.09,\;{\;\omega }_{2}=0.106,\;\omega_{3}=0.18,{\;\omega }_{4}=0.18,\;$ $\omega_{5}=0.18,\;\omega_{6}=0.168,\;\omega_{7}=0.076,\;\omega_{8}=0.02$	4.369 × 10^1^	−5.57%	14.11%
$\omega_{6}-20\%$	$\omega_{1}=0.12,\;{\;\omega }_{2}=0.126,\;{\;\omega }_{3}=0.18,{\;\omega }_{4}=0.18,$ $\omega_{5}=0.18,\;{\;\omega }_{6}=0.112,\;{\;\omega }_{7}=0.082,\;{\;\omega }_{8}=0.02$	5.022 × 10^1^	8.54%	

**Table 8:** Path-planning results for EAO and other algorithms.

Map	Algorithm	Best	Mean	Std	Mean Rank	Final Ranking	Number of Valid Trajectories	Average Runtime
1	PSO	5.397 × 10^1^	5.154 × 10^2^	3.901 × 10^2^	4.8	5	**30**	55.61
	SCA	1.214 × 10^2^	2.642 × 10^2^	8.333 × 10^1^	4.9	6	25	55.76
	GJO	4.387 × 10^1^	6.444 × 10^1^	1.782 × 10^1^	2.1	2	29	**55.44**
	WAA	8.516 × 10^1^	1.457 × 10^2^	6.112 × 10^1^	3.8	3	29	57.17
	AO	4.960 × 10^1^	2.434 × 10^2^	1.804 × 10^2^	4.2	4	24	56.06
	EAO	**3.803 × 10^1^**	**4.765 × 10^1^**	**8.517 × 10^0^**	**1.2**	1	**30**	55.82
2	PSO	1.270 × 10^2^	1.124 × 10^3^	4.759 × 10^2^	5.8	6	**29**	**56.21**
	SCA	1.583 × 10^2^	4.022 × 10^2^	1.396 × 10^2^	4.6	5	25	57.01
	GJO	3.968 × 10^1^	7.118 × 10^1^	5.635 × 10^1^	2.1	2	27	56.61
	WAA	1.085 × 10^2^	2.834 × 10^2^	1.240 × 10^2^	4.1	4	**29**	58.33
	AO	4.062 × 10^1^	2.218 × 10^2^	1.539 × 10^2^	3.3	3	24	57.16
	EAO	**3.784 × 10^1^**	**4.159 × 10^1^**	**1.000 × 10^1^**	**1.1**	1	**29**	57.04
3	PSO	1.612 × 10^2^	1.540 × 10^3^	2.377 × 10^3^	5.8	6	28	64.84
	SCA	1.917 × 10^2^	6.104 × 10^2^	2.522 × 10^2^	4.9	5	27	65.44
	GJO	4.410 × 10^1^	8.388 × 10^1^	1.023 × 10^2^	2.1	2	26	**64.81**
	WAA	1.302 × 10^2^	2.824 × 10^2^	1.344 × 10^2^	3.8	4	**30**	66.57
	AO	3.985 × 10^1^	2.458 × 10^2^	2.275 × 10^2^	3.2	3	29	65.25
	EAO	**3.881 × 10^1^**	**4.586 × 10^1^**	**5.188 × 10^0^**	**1.2**	1	29	65.91
4	PSO	1.038 × 10^2^	1.062 × 10^3^	4.628 × 10^2^	5.7	6	29	**66.16**
	SCA	1.760 × 10^2^	5.994 × 10^2^	2.117 × 10^2^	5	5	26	67.04
	GJO	4.293 × 10^1^	9.959 × 10^1^	3.632 × 10^1^	2.4	2	26	66.75
	WAA	7.752 × 10^1^	1.796 × 10^2^	7.037 × 10^1^	3.2	3	29	68.76
	AO	4.074 × 10^1^	3.072 × 10^2^	2.116 × 10^2^	3.5	4	23	67.54
	EAO	**3.789 × 10^1^**	**4.658 × 10^1^**	**2.103 × 10^1^**	**1.2**	1	**30**	67.65

## Data Availability

The data used to support the findings of this study are included in the article.

## Appendix A

### Appendix A.1

Time Complexity Analysis of EAO:

The time complexity of the original AO algorithm can be expressed as $T_{AO}=O\left(T\cdot N\cdot \left(logN+Dim+f\left(\cdot \right)\right)\right)$, which can be simplified to $T_{AO}=O\left(T\cdot N\cdot \left(Dim+f\left(\cdot \right)\right)\right)$.

The additional computational overhead introduced by EAO includes the following:

Search-state metrics calculation: O(N·Dim). This is executed only once per main loop iteration and can be neglected.

Dynamic neighborhood best calculation: $O(N^{2}\cdot Dim+N^{2}\cdot logN)$.

Adaptive behavior persistence mechanism: O(N). This includes trust-based behavior selection and reward-penalty updates, which involve only constant-time operations and array accesses, and can be neglected.

Periodic diversity maintenance: $O\left(\frac{N\cdot logN+0.15N\cdot f\left(\cdot \right)}{15}\right).$ Due to its low triggering frequency, this term can be neglected.

DE/current−to−lbest mutation strategy: O(Dim). This is comparable to the complexity of the original Lévy flight.

Therefore, the primary additional computational overhead of EAO comes from the dynamic neighborhood best calculation. The time complexity of EAO can be expressed as

$$T_{EAO}=O(T\cdot N\cdot \left(N\cdot logN+N\cdot Dim+f\left(\cdot \right))\right)$$

which can be simplified to

$$T_{EAO}=O(T\cdot N\cdot \left(N\cdot Dim+f\left(\cdot \right))\right)$$

While preserving the same dominant term of function evaluations as AO, EAO introduces controllable neighborhood computation overhead. At the cost of a modest increase in computational expense, EAO achieves significant improvements in optimization performance, striking an optimal balance between computational complexity and solution effectiveness.

### Appendix A.2

**Table A1:** CEC2017 benchmark functions.

	No.	Functions	$F_{i}^{*}\;=\;F_{i}(X^{*})$
Unimodal Functions	1	Shifted and Rotated Bent Cigar Function	100
	3	Shifted and Rotated Zakharov Function	200
Simple Multimodal Functions	4	Shifted and Rotated Rosenbrock’s Function	300
	5	Shifted and Rotated Rastrigin’s Function	400
	6	Shifted and Rotated Expanded Scaffer’s F6 Function	500
	7	Shifted and Rotated Lunacek Bi_Rastrigin Function	600
	8	Shifted and Rotated Non-Continuous Rastrigin’s Function	700
	9	Shifted and Rotated Lévy Function	800
	10	Shifted and Rotated Schwefel’s Function	900
Hybrid Functions	11	Hybrid Function 1 (N = 3)	1000
	12	Hybrid Function 2 (N = 3)	1100
	13	Hybrid Function 3 (N = 3)	1200
	14	Hybrid Function 4 (N = 4)	1300
	15	Hybrid Function 5 (N = 4)	1400
	16	Hybrid Function 6 (N = 4)	1500
	17	Hybrid Function 6 (N = 5)	1600
	18	Hybrid Function 6 (N = 5)	1700
	19	Hybrid Function 6 (N = 5)	1800
	20	Hybrid Function 6 (N = 6)	1900
Composition Functions	21	Composition Function 1 (N = 3)	2000
	22	Composition Function 2 (N = 3)	2100
	23	Composition Function 3 (N = 4)	2200
	24	Composition Function 4 (N = 4)	2300
	25	Composition Function 5 (N = 5)	2400
	26	Composition Function 6 (N = 5)	2500
	27	Composition Function 7 (N = 6)	2600
	28	Composition Function 8 (N = 6)	2700
	29	Composition Function 9 (N = 3)	2800
	30	Composition Function 10 (N = 3)	2900
$\mathrm{Search}\;\mathrm{Range}\text{:}\;{\left[-100,100\right]}^{D}$			

**Table A2:** CEC2020 benchmark functions.

Type	No.	Functions	$F_{i}^{*}\;=\;F_{i}(X^{*})$
Unimodal Functions	1	Shifted and Rotated Bent Cigar Function	100
Basic Functions	2	Shifted and Rotated Schwefel’s Function	1100
	3	Shifted and Rotated Rastrigin’s Function	700
	4	Shifted and Rotated Lunacek bi-Rastrigin Function	1900
Hybrid Functions	5	Hybrid Function 1 (N = 3)	1700
	6	Hybrid Function 2 (N = 3)	1600
	7	Hybrid Function 3 (N = 3)	2100
Composition Functions	8	Composition Function 1 (N = 3)	2200
	9	Composition Function 2 (N = 4)	2400
	10	Composition Function 3 (N = 5)	2500
Search Range: ${\left[-100,100\right]}^{D}$			

**Table A3:** Sensitivity analysis results for parameter $R_{P}$ on selected CEC2017 functions.

Function	Index	$R_{P}$				
		10	15	20	25	30
F1	Mean	4.489 × 10^7^	4.141 × 10^7^	4.976 × 10^7^	4.800 × 10^7^	4.023 × 10^7^
	Std	3.486 × 10^7^	2.261 × 10^7^	3.260 × 10^7^	3.485 × 10^7^	1.939 × 10^7^
	Rank	3	2	5	4	1
F3	Mean	6.191 × 10^3^	5.940 × 10^3^	5.360 × 10^3^	5.750 × 10^3^	7.035 × 10^3^
	Std	2.580 × 10^3^	2.263 × 10^3^	1.863 × 10^3^	2.592 × 10^3^	3.719 × 10^3^
	Rank	4	3	1	2	5
F6	Mean	6.301 × 10^2^	6.290 × 10^2^	6.319 × 10^2^	6.278 × 10^2^	6.270 × 10^2^
	Std	1.504 × 10^1^	1.352 × 10^1^	1.652 × 10^1^	1.424 × 10^1^	1.504 × 10^1^
	Rank	4	3	5	2	1
F9	Mean	3.145 × 10^3^	4.244 × 10^3^	3.241 × 10^3^	3.893 × 10^3^	3.932 × 10^3^
	Std	1.514 × 10^3^	1.616 × 10^3^	1.435 × 10^3^	2.150 × 10^3^	2.014 × 10^3^
	Rank	1	5	2	3	4
F11	Mean	1.364 × 10^3^	1.346 × 10^3^	1.371 × 10^3^	1.357 × 10^3^	1.368 × 10^3^
	Std	6.324 × 10^1^	5.917 × 10^1^	7.398 × 10^1^	6.660 × 10^1^	7.422 × 10^1^
	Rank	2	1	5	3	4
F12	Mean	2.020 × 10^7^	1.647 × 10^7^	2.337 × 10^7^	1.773 × 10^7^	2.308 × 10^7^
	Std	2.531 × 10^7^	1.624 × 10^7^	1.876 × 10^7^	1.970 × 10^7^	1.782 × 10^7^
	Rank	3	1	5	2	4
F13	Mean	1.810 × 10^5^	2.622 × 10^5^	1.531 × 10^5^	2.785 × 10^5^	2.001 × 10^5^
	Std	7.972 × 10^4^	2.530 × 10^5^	7.033 × 10^4^	2.756 × 10^5^	8.758 × 10^4^
	Rank	2	4	1	5	3
F15	Mean	6.307 × 10^4^	5.902 × 10^4^	7.277 × 10^4^	5.750 × 10^4^	5.698 × 10^4^
	Std	5.029 × 10^4^	4.272 × 10^4^	5.242 × 10^4^	3.762 × 10^4^	3.946 × 10^4^
	Rank	4	3	5	1	2
F24	Mean	2.957 × 10^3^	2.942 × 10^3^	2.942 × 10^3^	2.953 × 10^3^	2.944 × 10^3^
	Std	5.739 × 10^1^	3.611 × 10^1^	3.216 × 10^1^	7.284 × 10^1^	4.621 × 10^1^
	Rank	4	2	1	5	3
F27	Mean	3.234 × 10^3^	3.249 × 10^3^	3.237 × 10^3^	3.240 × 10^3^	3.258 × 10^3^
	Std	2.080 × 10^1^	3.443 × 10^1^	2.304 × 10^1^	2.709 × 10^1^	9.187 × 10^1^
	Rank	1	4	2	3	5
F29	Mean	3.926 × 10^3^	3.908 × 10^3^	3.975 × 10^3^	3.963 × 10^3^	3.911 × 10^3^
	Std	2.076 × 10^2^	1.798 × 10^2^	2.508 × 10^2^	2.280 × 10^2^	2.260 × 10^2^
	Rank	3	1	5	4	2
F30	Mean	6.255 × 10^6^	6.110 × 10^6^	6.917 × 10^6^	7.987 × 10^6^	6.746 × 10^6^
	Std	3.363 × 10^6^	3.546 × 10^6^	4.873 × 10^6^	7.998 × 10^6^	4.505 × 10^6^
	Rank	2	1	4	5	3
Mean Rank		2.75	2.5	3.42	3.25	3.08

**Table A4:** Sensitivity analysis results for parameter N on selected CEC2017 functions.

Function	Index	*N*				
		10	20	30	50	100
F1	Mean	1.385 × 10^7^	3.381 × 10^7^	3.384 × 10^8^	6.563 × 10^7^	3.781 × 10^9^
	Std	7.719 × 10^6^	1.603 × 10^7^	4.412 × 10^8^	3.278 × 10^7^	2.462 × 10^9^
	Rank	1	2	4	3	5
F3	Mean	3.522 × 10^3^	4.294 × 10^3^	1.022 × 10^4^	6.664 × 10^3^	1.811 × 10^4^
	Std	1.680 × 10^3^	1.656 × 10^3^	4.019 × 10^3^	2.650 × 10^3^	8.624 × 10^3^
	Rank	1	2	4	3	5
F6	Mean	6.298 × 10^2^	6.288 × 10^2^	6.290 × 10^2^	6.289 × 10^2^	6.304 × 10^2^
	Std	1.586 × 10^1^	1.325 × 10^1^	1.547 × 10^1^	1.081 × 10^1^	9.520 × 10^0^
	Rank	4	1	3	2	5
F9	Mean	3.744 × 10^3^	3.668 × 10^3^	3.419 × 10^3^	4.019 × 10^3^	3.249 × 10^3^
	Std	2.187 × 10^3^	2.476 × 10^3^	1.678 × 10^3^	1.933 × 10^3^	1.724 × 10^3^
	Rank	4	3	2	5	1
F11	Mean	1.363 × 10^3^	1.353 × 10^3^	1.347 × 10^3^	1.355 × 10^3^	1.343 × 10^3^
	Std	7.561 × 10^1^	5.983 × 10^1^	6.173 × 10^1^	6.825 × 10^1^	6.670 × 10^1^
	Rank	5	3	2	4	1
F12	Mean	1.769 × 10^7^	2.252 × 10^7^	2.222 × 10^7^	1.484 × 10^7^	1.881 × 10^8^
	Std	2.001 × 10^7^	2.050 × 10^7^	2.298 × 10^7^	9.198 × 10^6^	1.913 × 10^8^
	Rank	2	4	3	1	5
F13	Mean	1.935 × 10^5^	2.498 × 10^5^	2.051 × 10^5^	3.521 × 10^5^	1.779 × 10^5^
	Std	1.205 × 10^5^	1.510 × 10^5^	1.160 × 10^5^	5.172 × 10^5^	2.432 × 10^5^
	Rank	2	4	3	5	1
F15	Mean	6.392 × 10^4^	6.225 × 10^4^	4.380 × 10^4^	6.663 × 10^4^	2.716 × 10^4^
	Std	4.338 × 10^4^	3.835 × 10^4^	3.058 × 10^4^	4.643 × 10^4^	1.588 × 10^4^
	Rank	4	3	2	5	1
F24	Mean	2.921 × 10^3^	2.924 × 10^3^	2.969 × 10^3^	2.930 × 10^3^	2.961 × 10^3^
	Std	5.092 × 10^1^	3.472 × 10^1^	6.857 × 10^1^	4.394 × 10^1^	4.009 × 10^1^
	Rank	1	2	5	3	4
F27	Mean	3.225 × 10^3^	3.231 × 10^3^	3.265 × 10^3^	3.242 × 10^3^	3.342 × 10^3^
	Std	2.034 × 10^1^	2.187 × 10^1^	6.679 × 10^1^	3.016 × 10^1^	8.006 × 10^1^
	Rank	1	2	4	3	5
F29	Mean	3.924 × 10^3^	3.987 × 10^3^	3.970 × 10^3^	3.949 × 10^3^	4.027 × 10^3^
	Std	1.930 × 10^2^	2.304 × 10^2^	2.057 × 10^2^	2.193 × 10^2^	2.206 × 10^2^
	Rank	1	4	3	2	5
F30	Mean	7.360 × 10^6^	6.904 × 10^6^	7.329 × 10^6^	7.471 × 10^6^	8.810 × 10^6^
	Std	6.738 × 10^6^	7.369 × 10^6^	5.986 × 10^6^	4.522 × 10^6^	9.471 × 10^6^
	Rank	3	1	2	4	5
Mean Rank		2.4	2.6	3.1	3.3	3.6

**Table A5:** Sensitivity analysis results for parameter τ on selected CEC2017 functions.

Function	Index	τ				
		3	5	7	20	15
F1	Mean	2.928 × 10^7^	4.577 × 10^7^	7.303 × 10^7^	1.377 × 10^8^	3.790 × 10^8^
	Std	1.662 × 10^7^	2.375 × 10^7^	3.460 × 10^7^	8.724 × 10^7^	2.256 × 10^8^
	Rank	1	2	3	4	5
F3	Mean	4.165 × 10^3^	5.955 × 10^3^	8.420 × 10^3^	1.147 × 10^4^	1.412 × 10^4^
	Std	1.345 × 10^3^	2.105 × 10^3^	2.588 × 10^3^	3.747 × 10^3^	4.091 × 10^3^
	Rank	1	2	3	4	5
F6	Mean	6.332 × 10^2^	6.274 × 10^2^	6.320 × 10^2^	6.314 × 10^2^	6.368 × 10^2^
	Std	1.706 × 10^1^	1.162 × 10^1^	1.571 × 10^1^	1.418 × 10^1^	1.331 × 10^1^
	Rank	4	1	3	2	5
F9	Mean	3.390 × 10^3^	3.450 × 10^3^	4.744 × 10^3^	5.030 × 10^3^	3.905 × 10^3^
	Std	1.860 × 10^3^	1.647 × 10^3^	2.434 × 10^3^	2.605 × 10^3^	1.988 × 10^3^
	Rank	1	2	4	5	3
F11	Mean	1.334 × 10^3^	1.363 × 10^3^	1.383 × 10^3^	1.366 × 10^3^	1.431 × 10^3^
	Std	6.274 × 10^1^	5.609 × 10^1^	5.325 × 10^1^	7.544 × 10^1^	8.848 × 10^1^
	Rank	1	2	4	3	5
F12	Mean	2.531 × 10^7^	2.721 × 10^7^	2.831 × 10^7^	2.500 × 10^7^	4.186 × 10^7^
	Std	2.538 × 10^7^	2.947 × 10^7^	3.353 × 10^7^	2.068 × 10^7^	3.875 × 10^7^
	Rank	2	3	4	1	5
F13	Mean	1.648 × 10^5^	2.318 × 10^5^	2.472 × 10^5^	3.038 × 10^5^	5.465 × 10^5^
	Std	8.005 × 10^4^	1.580 × 10^5^	1.615 × 10^5^	2.389 × 10^5^	4.102 × 10^5^
	Rank	1	2	3	4	5
F15	Mean	6.890 × 10^4^	5.104 × 10^4^	6.626 × 10^4^	6.320 × 10^4^	7.973 × 10^4^
	Std	4.298 × 10^4^	2.838 × 10^4^	4.857 × 10^4^	3.501 × 10^4^	5.529 × 10^4^
	Rank	4	1	3	2	5
F24	Mean	2.926 × 10^3^	2.951 × 10^3^	2.956 × 10^3^	2.959 × 10^3^	2.986 × 10^3^
	Std	3.466 × 10^1^	3.340 × 10^1^	8.248 × 10^1^	3.566 × 10^1^	5.240 × 10^1^
	Rank	1	2	3	4	5
F27	Mean	3.235 × 10^3^	3.247 × 10^3^	3.240 × 10^3^	3.246 × 10^3^	3.261 × 10^3^
	Std	3.677 × 10^1^	3.330 × 10^1^	2.537 × 10^1^	2.854 × 10^1^	3.596 × 10^1^
	Rank	1	4	2	3	5
F29	Mean	3.959 × 10^3^	3.882 × 10^3^	3.922 × 10^3^	3.991 × 10^3^	4.027 × 10^3^
	Std	2.308 × 10^2^	1.926 × 10^2^	1.744 × 10^2^	1.761 × 10^2^	2.111 × 10^2^
	Rank	3	1	2	4	5
F30	Mean	6.278 × 10^6^	7.122 × 10^6^	6.967 × 10^6^	7.574 × 10^6^	6.875 × 10^6^
	Std	5.774 × 10^6^	5.282 × 10^6^	4.626 × 10^6^	5.989 × 10^6^	4.376 × 10^6^
	Rank	1	4	3	5	2
Mean Rank		1.8	2.2	3.1	3.4	4.6

**Table A6:** Sensitivity analysis results for parameter $k_{min}$ on selected CEC2017 functions.

Function	Index	$k_{min}$				
		2	3	4	5	6
F1	Mean	6.219 × 10^7^	5.388 × 10^7^	5.672 × 10^7^	5.249 × 10^7^	6.623 × 10^7^
	Std	3.019 × 10^7^	2.958 × 10^7^	2.351 × 10^7^	2.519 × 10^7^	3.980 × 10^7^
	Rank	4	2	3	1	5
F3	Mean	7.087 × 10^3^	5.862 × 10^3^	6.506 × 10^3^	6.827 × 10^3^	7.424 × 10^3^
	Std	2.221 × 10^3^	2.357 × 10^3^	2.511 × 10^3^	2.864 × 10^3^	2.902 × 10^3^
	Rank	4	1	2	3	5
F6	Mean	6.323 × 10^2^	6.290 × 10^2^	6.321 × 10^2^	6.262 × 10^2^	6.352 × 10^2^
	Std	1.370 × 10^1^	1.289 × 10^1^	1.566 × 10^1^	1.418 × 10^1^	1.340 × 10^1^
	Rank	4	2	3	1	5
F9	Mean	3.717 × 10^3^	2.977 × 10^3^	4.500 × 10^3^	3.575 × 10^3^	4.056 × 10^3^
	Std	2.215 × 10^3^	2.039 × 10^3^	2.409 × 10^3^	1.739 × 10^3^	2.130 × 10^3^
	Rank	3	1	5	2	4
F11	Mean	1.380 × 10^3^	1.330 × 10^3^	1.335 × 10^3^	1.365 × 10^3^	1.359 × 10^3^
	Std	6.006 × 10^1^	5.926 × 10^1^	7.012 × 10^1^	7.000 × 10^1^	5.078 × 10^1^
	Rank	5	1	2	4	3
F12	Mean	1.827 × 10^7^	2.196 × 10^7^	2.260 × 10^7^	2.539 × 10^7^	2.122 × 10^7^
	Std	1.686 × 10^7^	2.586 × 10^7^	1.536 × 10^7^	1.741 × 10^7^	1.495 × 10^7^
	Rank	1	3	4	5	2
F13	Mean	2.716 × 10^5^	2.064 × 10^5^	2.112 × 10^5^	2.534 × 10^5^	2.767 × 10^5^
	Std	2.721 × 10^5^	8.036 × 10^4^	1.318 × 10^5^	1.318 × 10^5^	2.095 × 10^5^
	Rank	4	1	2	3	5
F15	Mean	5.047 × 10^4^	5.519 × 10^4^	4.282 × 10^4^	6.764 × 10^4^	6.432 × 10^4^
	Std	3.628 × 10^4^	2.762 × 10^4^	2.537 × 10^4^	5.256 × 10^4^	6.122 × 10^4^
	Rank	2	3	1	5	4
F24	Mean	2.942 × 10^3^	2.941 × 10^3^	2.943 × 10^3^	2.931 × 10^3^	2.942 × 10^3^
	Std	3.508 × 10^1^	3.882 × 10^1^	4.283 × 10^1^	3.928 × 10^1^	5.553 × 10^1^
	Rank	4	2	5	1	3
F27	Mean	3.238 × 10^3^	3.236 × 10^3^	3.242 × 10^3^	3.243 × 10^3^	3.247 × 10^3^
	Std	2.210 × 10^1^	2.489 × 10^1^	2.475 × 10^1^	3.176 × 10^1^	2.595 × 10^1^
	Rank	2	1	3	4	5
F29	Mean	4.012 × 10^3^	3.929 × 10^3^	4.042 × 10^3^	3.920 × 10^3^	4.015 × 10^3^
	Std	1.846 × 10^2^	2.135 × 10^2^	2.174 × 10^2^	2.240 × 10^2^	2.530 × 10^2^
	Rank	3	2	5	1	4
F30	Mean	6.048 × 10^6^	7.337 × 10^6^	7.560 × 10^6^	7.366 × 10^6^	5.742 × 10^6^
	Std	3.423 × 10^6^	4.557 × 10^6^	5.252 × 10^6^	6.382 × 10^6^	5.011 × 10^6^
	Rank	2	3	5	4	1
Mean Rank		3.2	1.8	3.3	2.8	3.8

**Table A7:** Sensitivity analysis results for parameter μ on selected CEC2017 functions.

Function	Index	μ				
		0.15	0.2	0.3	0.4	0.5
F1	Mean	4.675 × 10^7^	7.221 × 10^7^	5.882 × 10^7^	6.566 × 10^7^	6.132 × 10^7^
	Std	2.427 × 10^7^	4.930 × 10^7^	3.527 × 10^7^	2.645 × 10^7^	2.554 × 10^7^
	Rank	1	5	2	4	3
F3	Mean	6.858 × 10^3^	6.227 × 10^3^	5.532 × 10^3^	6.059 × 10^3^	6.473 × 10^3^
	Std	2.806 × 10^3^	2.416 × 10^3^	1.875 × 10^3^	1.845 × 10^3^	2.811 × 10^3^
	Rank	5	3	1	2	4
F6	Mean	6.320 × 10^2^	6.332 × 10^2^	6.275 × 10^2^	6.291 × 10^2^	6.298 × 10^2^
	Std	1.680 × 10^1^	1.639 × 10^1^	1.270 × 10^1^	1.246 × 10^1^	1.547 × 10^1^
	Rank	4	5	1	2	3
F9	Mean	3.477 × 10^3^	3.460 × 10^3^	3.361 × 10^3^	3.509 × 10^3^	3.104 × 10^3^
	Std	2.112 × 10^3^	1.994 × 10^3^	1.822 × 10^3^	1.825 × 10^3^	1.657 × 10^3^
	Rank	4	3	2	5	1
F11	Mean	1.366 × 10^3^	1.345 × 10^3^	1.369 × 10^3^	1.366 × 10^3^	1.344 × 10^3^
	Std	5.839 × 10^1^	6.728 × 10^1^	5.927 × 10^1^	6.856 × 10^1^	6.074 × 10^1^
	Rank	4	2	5	3	1
F12	Mean	2.068 × 10^7^	1.587 × 10^7^	2.223 × 10^7^	2.390 × 10^7^	2.309 × 10^7^
	Std	2.703 × 10^7^	1.367 × 10^7^	1.709 × 10^7^	2.006 × 10^7^	2.164 × 10^7^
	Rank	2	1	3	5	4
F13	Mean	3.076 × 10^5^	2.893 × 10^5^	2.337 × 10^5^	2.175 × 10^5^	1.959 × 10^5^
	Std	1.571 × 10^5^	2.532 × 10^5^	1.555 × 10^5^	1.134 × 10^5^	1.291 × 10^5^
	Rank	5	4	3	2	1
F15	Mean	6.747 × 10^4^	5.814 × 10^4^	7.546 × 10^4^	5.050 × 10^4^	4.149 × 10^4^
	Std	3.726 × 10^4^	3.644 × 10^4^	6.018 × 10^4^	3.217 × 10^4^	3.443 × 10^4^
	Rank	4	3	5	2	1
F24	Mean	2.927 × 10^3^	2.952 × 10^3^	2.958 × 10^3^	2.937 × 10^3^	2.950 × 10^3^
	Std	3.271 × 10^1^	3.563 × 10^1^	6.604 × 10^1^	2.980 × 10^1^	4.125 × 10^1^
	Rank	1	4	5	2	3
F27	Mean	3.235 × 10^3^	3.237 × 10^3^	3.244 × 10^3^	3.255 × 10^3^	3.234 × 10^3^
	Std	1.818 × 10^1^	2.745 × 10^1^	4.726 × 10^1^	5.338 × 10^1^	2.446 × 10^1^
	Rank	2	3	4	5	1
F29	Mean	3.936 × 10^3^	4.028 × 10^3^	4.032 × 10^3^	3.965 × 10^3^	3.925 × 10^3^
	Std	2.374 × 10^2^	2.911 × 10^2^	2.766 × 10^2^	2.842 × 10^2^	2.005 × 10^2^
	Rank	2	4	5	3	1
F30	Mean	6.213 × 10^6^	7.459 × 10^6^	6.207 × 10^6^	7.622 × 10^6^	6.886 × 10^6^
	Std	5.104 × 10^6^	7.570 × 10^6^	4.258 × 10^6^	4.877 × 10^6^	5.655 × 10^6^
	Rank	2	4	1	5	3
Mean Rank		3	3.4	3.1	3.3	2.2

**Table A8:** Sensitivity analysis results for parameter $\theta_{L}$ on selected CEC2017 functions.

Function	Index	$\theta_{L}$				
		0.15	0.2	0.3	0.4	0.5
F1	Mean	5.577 × 10^7^	5.791 × 10^7^	4.868 × 10^7^	4.505 × 10^7^	6.109 × 10^7^
	Std	2.021 × 10^7^	2.641 × 10^7^	1.971 × 10^7^	1.935 × 10^7^	3.047 × 10^7^
	Rank	3	4	2	1	5
F3	Mean	6.461 × 10^3^	7.045 × 10^3^	7.339 × 10^3^	6.698 × 10^3^	7.273 × 10^3^
	Std	2.520 × 10^3^	2.693 × 10^3^	3.294 × 10^3^	2.815 × 10^3^	3.668 × 10^3^
	Rank	1	3	5	2	4
F6	Mean	6.320 × 10^2^	6.325 × 10^2^	6.328 × 10^2^	6.292 × 10^2^	6.324 × 10^2^
	Std	1.712 × 10^1^	1.676 × 10^1^	1.768 × 10^1^	1.159 × 10^1^	1.771 × 10^1^
	Rank	2	4	5	1	3
F9	Mean	4.037 × 10^3^	3.422 × 10^3^	4.345 × 10^3^	4.357 × 10^3^	4.306 × 10^3^
	Std	2.529 × 10^3^	1.703 × 10^3^	2.237 × 10^3^	2.141 × 10^3^	2.105 × 10^3^
	Rank	2	1	4	5	3
F11	Mean	1.364 × 10^3^	1.352 × 10^3^	1.366 × 10^3^	1.369 × 10^3^	1.334 × 10^3^
	Std	5.514 × 10^1^	6.149 × 10^1^	6.667 × 10^1^	9.130 × 10^1^	7.804 × 10^1^
	Rank	3	2	4	5	1
F12	Mean	2.083 × 10^7^	3.619 × 10^7^	1.775 × 10^7^	1.775 × 10^7^	1.596 × 10^7^
	Std	2.303 × 10^7^	3.186 × 10^7^	1.874 × 10^7^	1.564 × 10^7^	1.649 × 10^7^
	Rank	4	5	2	3	1
F13	Mean	2.502 × 10^5^	2.431 × 10^5^	2.039 × 10^5^	2.234 × 10^5^	2.256 × 10^5^
	Std	1.510 × 10^5^	1.405 × 10^5^	1.052 × 10^5^	1.523 × 10^5^	1.236 × 10^5^
	Rank	5	4	1	2	3
F15	Mean	6.058 × 10^4^	5.855 × 10^4^	5.697 × 10^4^	5.393 × 10^4^	5.394 × 10^4^
	Std	3.703 × 10^4^	4.918 × 10^4^	4.074 × 10^4^	3.037 × 10^4^	4.461 × 10^4^
	Rank	5	4	3	1	2
F24	Mean	2.938 × 10^3^	2.935 × 10^3^	2.946 × 10^3^	2.923 × 10^3^	2.945 × 10^3^
	Std	2.222 × 10^1^	3.875 × 10^1^	4.608 × 10^1^	2.047 × 10^1^	4.310 × 10^1^
	Rank	3	2	5	1	4
F27	Mean	3.247 × 10^3^	3.241 × 10^3^	3.239 × 10^3^	3.235 × 10^3^	3.237 × 10^3^
	Std	4.719 × 10^1^	2.261 × 10^1^	2.765 × 10^1^	2.859 × 10^1^	2.747 × 10^1^
	Rank	5	4	3	1	2
F29	Mean	3.924 × 10^3^	3.957 × 10^3^	3.949 × 10^3^	3.904 × 10^3^	4.029 × 10^3^
	Std	2.211 × 10^2^	2.400 × 10^2^	2.121 × 10^2^	2.351 × 10^2^	2.331 × 10^2^
	Rank	2	4	3	1	5
F30	Mean	6.923 × 10^6^	6.254 × 10^6^	7.038 × 10^6^	4.548 × 10^6^	8.313 × 10^6^
	Std	5.113 × 10^6^	5.007 × 10^6^	6.066 × 10^6^	4.187 × 10^6^	6.088 × 10^6^
	Rank	3	2	4	1	5
Mean Rank		3.2	3.3	3.4	2	3.2

**Table A9:** Sensitivity analysis results for parameter $\theta_{H}$ on selected CEC2017 functions.

Function	Index	$\theta_{H}$				
		0.25	0.3	0.35	0.4	0.5
F1	Mean	6.132 × 10^7^	6.159 × 10^7^	5.517 × 10^7^	6.550 × 10^7^	6.165 × 10^7^
	Std	2.620 × 10^7^	2.942 × 10^7^	3.086 × 10^7^	3.135 × 10^7^	1.796 × 10^7^
	Rank	2	3	1	5	4
F3	Mean	6.626 × 10^3^	6.282 × 10^3^	6.551 × 10^3^	7.461 × 10^3^	5.395 × 10^3^
	Std	3.044 × 10^3^	3.178 × 10^3^	2.832 × 10^3^	3.464 × 10^3^	2.185 × 10^3^
	Rank	4	2	3	5	1
F6	Mean	6.314 × 10^2^	6.314 × 10^2^	6.357 × 10^2^	6.282 × 10^2^	6.322 × 10^2^
	Std	1.429 × 10^1^	1.436 × 10^1^	1.495 × 10^1^	1.237 × 10^1^	1.484 × 10^1^
	Rank	3	2	5	1	4
F9	Mean	3.776 × 10^3^	4.572 × 10^3^	3.560 × 10^3^	3.856 × 10^3^	3.621 × 10^3^
	Std	1.740 × 10^3^	2.228 × 10^3^	1.841 × 10^3^	2.057 × 10^3^	2.234 × 10^3^
	Rank	3	5	1	4	2
F11	Mean	1.357 × 10^3^	1.346 × 10^3^	1.348 × 10^3^	1.349 × 10^3^	1.356 × 10^3^
	Std	5.868 × 10^1^	6.584 × 10^1^	5.849 × 10^1^	6.833 × 10^1^	5.504 × 10^1^
	Rank	5	1	2	3	4
F12	Mean	2.291 × 10^7^	2.047 × 10^7^	1.734 × 10^7^	2.133 × 10^7^	2.010 × 10^7^
	Std	2.314 × 10^7^	1.557 × 10^7^	1.964 × 10^7^	1.557 × 10^7^	1.775 × 10^7^
	Rank	5	3	1	4	2
F13	Mean	2.426 × 10^5^	2.283 × 10^5^	2.201 × 10^5^	2.095 × 10^5^	2.174 × 10^5^
	Std	1.822 × 10^5^	1.280 × 10^5^	1.140 × 10^5^	1.156 × 10^5^	1.122 × 10^5^
	Rank	5	4	3	1	2
F15	Mean	5.227 × 10^4^	7.405 × 10^4^	6.525 × 10^4^	6.652 × 10^4^	4.693 × 10^4^
	Std	2.670 × 10^4^	8.869 × 10^4^	4.492 × 10^4^	5.087 × 10^4^	2.887 × 10^4^
	Rank	2	5	3	4	1
F24	Mean	2.947 × 10^3^	2.945 × 10^3^	2.953 × 10^3^	2.943 × 10^3^	2.920 × 10^3^
	Std	4.234 × 10^1^	3.417 × 10^1^	3.467 × 10^1^	4.076 × 10^1^	7.998 × 10^1^
	Rank	4	3	5	2	1
F27	Mean	3.243 × 10^3^	3.234 × 10^3^	3.239 × 10^3^	3.233 × 10^3^	3.248 × 10^3^
	Std	2.811 × 10^1^	1.678 × 10^1^	2.068 × 10^1^	2.412 × 10^1^	5.960 × 10^1^
	Rank	4	2	3	1	5
F29	Mean	4.011 × 10^3^	3.945 × 10^3^	3.999 × 10^3^	3.933 × 10^3^	3.931 × 10^3^
	Std	3.433 × 10^2^	2.047 × 10^2^	2.001 × 10^2^	2.101 × 10^2^	2.017 × 10^2^
	Rank	5	3	4	2	1
F30	Mean	6.818 × 10^6^	7.759 × 10^6^	6.478 × 10^6^	7.115 × 10^6^	7.644 × 10^6^
	Std	5.267 × 10^6^	4.023 × 10^6^	4.596 × 10^6^	4.992 × 10^6^	6.326 × 10^6^
	Rank	2	5	1	3	4
Mean Rank		3.7	3.2	2.7	2.9	2.6

**Table A10:** Comparison results with classic highly-cited algorithms on CEC2017 (Dim = 30).

Function	Index	EAO	PSO	DE	CMA-ES	WOA	SCA	MFO	HHO
F1	Best	3.859 × 10^5^	2.218 × 10^10^	1.227 × 10^9^	**1.005 × 10^2^**	4.227 × 10^8^	1.206 × 10^10^	1.027 × 10^9^	3.064 × 10^10^
	Mean	5.958 × 10^7^	3.254 × 10^10^	2.210 × 10^9^	2.245 × 10^9^	1.085 × 10^9^	1.816 × 10^10^	1.275 × 10^10^	4.736 × 10^10^
	Std	1.359 × 10^8^	5.778 × 10^9^	4.534 × 10^8^	8.583 × 10^9^	4.597 × 10^8^	2.992 × 10^9^	7.197 × 10^9^	8.443 × 10^9^
	P	~	1.734 × 10^−6^	1.734 × 10^−6^	3.589 × 10^−4^	1.921 × 10^−6^	1.734 × 10^−6^	1.734 × 10^−6^	1.734 × 10^−6^
	Rank	3	13	6	1	5	11	9	14
F3	Best	**2.121 × 10^3^**	1.068 × 10^5^	1.494 × 10^5^	9.994 × 10^4^	1.264 × 10^5^	4.174 × 10^4^	5.314 × 10^4^	7.348 × 10^4^
	Mean	**4.474 × 10^3^**	2.008 × 10^5^	1.967 × 10^5^	2.020 × 10^5^	2.610 × 10^5^	6.538 × 10^4^	1.516 × 10^5^	9.121 × 10^4^
	Std	**1.568 × 10^3^**	3.986 × 10^4^	2.927 × 10^4^	4.472 × 10^4^	7.614 × 10^4^	1.177 × 10^4^	5.974 × 10^4^	4.315 × 10^3^
	P	~	1.734 × 10^−6^	1.734 × 10^−6^	1.734 × 10^−6^	1.734 × 10^−6^	1.734 × 10^−6^	1.734 × 10^−6^	1.734 × 10^−6^
	Rank	1	14	13	12	15	6	11	10
F4	Best	4.873 × 10^2^	1.636 × 10^3^	6.358 × 10^2^	1.532 × 10^3^	5.779 × 10^2^	1.565 × 10^3^	5.624 × 10^2^	5.308 × 10^3^
	Mean	**5.078 × 10^2^**	3.900 × 10^3^	7.276 × 10^2^	3.304 × 10^3^	7.376 × 10^2^	2.470 × 10^3^	1.374 × 10^3^	1.148 × 10^4^
	Std	2.753 × 10^1^	2.166 × 10^3^	6.044 × 10^1^	1.271 × 10^3^	1.028 × 10^2^	7.510 × 10^2^	8.530 × 10^2^	3.903 × 10^3^
	P	~	1.734 × 10^−6^	1.734 × 10^−6^	1.734 × 10^−6^	1.734 × 10^−6^	1.734 × 10^−6^	1.734 × 10^−6^	1.734 × 10^−6^
	Rank	1	12	6	11	5	10	7	14
F5	Best	5.601 × 10^2^	8.104 × 10^2^	7.162 × 10^2^	**5.129 × 10^2^**	7.090 × 10^2^	7.688 × 10^2^	6.453 × 10^2^	7.939 × 10^2^
	Mean	**6.189 × 10^2^**	8.525 × 10^2^	7.387 × 10^2^	7.113 × 10^2^	8.258 × 10^2^	8.136 × 10^2^	7.059 × 10^2^	9.052 × 10^2^
	Std	2.985 × 10^1^	2.092 × 10^1^	**1.204 × 10^1^**	1.141 × 10^2^	6.326 × 10^1^	2.517 × 10^1^	3.750 × 10^1^	5.190 × 10^1^
	P	~	1.734 × 10^−6^	1.734 × 10^−6^	8.307 × 10^−4^	1.734 × 10^−6^	1.734 × 10^−6^	1.734 × 10^−6^	1.734 × 10^−6^
	Rank	1	13	6	5	10	9	3	14
F6	Best	6.075 × 10^2^	6.516 × 10^2^	6.151 × 10^2^	**6.000 × 10^2^**	6.503 × 10^2^	6.496 × 10^2^	6.174 × 10^2^	6.508 × 10^2^
	Mean	6.310 × 10^2^	6.705 × 10^2^	**6.207 × 10^2^**	6.474 × 10^2^	6.755 × 10^2^	6.603 × 10^2^	6.389 × 10^2^	6.847 × 10^2^
	Std	1.627 × 10^1^	9.930 × 10^0^	**2.424 × 10^0^**	2.736 × 10^1^	1.237 × 10^1^	6.100 × 10^0^	1.475 × 10^1^	1.060 × 10^1^
	P	~	2.879 × 10^−6^	5.320 × 10^−3^	1.566 × 10^−2^	1.734 × 10^−6^	4.729 × 10^−6^	8.221 × 10^−2^	1.734 × 10^−6^
	Rank	2	10	1	6	11	7	4	14
F7	Best	8.292 × 10^2^	1.730 × 10^3^	1.039 × 10^3^	**7.116 × 10^2^**	1.149 × 10^3^	1.078 × 10^3^	8.623 × 10^2^	1.245 × 10^3^
	Mean	8.847 × 10^2^	2.048 × 10^3^	1.111 × 10^3^	**8.779 × 10^2^**	1.293 × 10^3^	1.204 × 10^3^	1.167 × 10^3^	1.462 × 10^3^
	Std	**3.054 × 10^1^**	1.378 × 10^2^	3.178 × 10^1^	6.235 × 10^1^	6.948 × 10^1^	4.585 × 10^1^	1.864 × 10^2^	5.443 × 10^1^
	P	~	1.734 × 10^−6^	1.734 × 10^−6^	8.936 × 10^−1^	1.734 × 10^−6^	1.734 × 10^−6^	2.127 × 10^−6^	1.734 × 10^−6^
	Rank	1	15	5	2	10	7	6	14
F8	Best	8.540 × 10^2^	1.136 × 10^3^	1.003 × 10^3^	**8.119 × 10^2^**	9.727 × 10^2^	1.035 × 10^3^	9.084 × 10^2^	1.064 × 10^3^
	Mean	9.361 × 10^2^	1.165 × 10^3^	1.053 × 10^3^	**9.324 × 10^2^**	1.040 × 10^3^	1.081 × 10^3^	9.961 × 10^2^	1.124 × 10^3^
	Std	3.987 × 10^1^	2.128 × 10^1^	1.864 × 10^1^	1.127 × 10^2^	5.301 × 10^1^	1.886 × 10^1^	4.570 × 10^1^	2.941 × 10^1^
	P	~	1.734 × 10^−6^	1.734 × 10^−6^	7.036 × 10^−1^	2.353 × 10^−6^	1.734 × 10^−6^	1.742 × 10^−4^	1.734 × 10^−6^
	Rank	1	15	11	4	8	12	5	13
F9	Best	1.056 × 10^3^	8.470 × 10^3^	7.668 × 10^3^	**9.000 × 10^2^**	6.505 × 10^3^	5.735 × 10^3^	3.639 × 10^3^	7.861 × 10^3^
	Mean	3.409 × 10^3^	1.429 × 10^4^	9.851 × 10^3^	**9.000 × 10^2^**	1.119 × 10^4^	7.618 × 10^3^	7.205 × 10^3^	1.024 × 10^4^
	Std	1.702 × 10^3^	2.709 × 10^3^	1.147 × 10^3^	**8.295 × 10^−2^**	3.209 × 10^3^	1.169 × 10^3^	2.085 × 10^3^	1.604 × 10^3^
	P	~	1.734 × 10^−6^	1.734 × 10^−6^	1.734 × 10^−6^	1.734 × 10^−6^	2.353 × 10^−6^	2.879 × 10^−6^	1.734 × 10^−6^
	Rank	2	15	11	1	13	10	7	12
F10	Best	**2.873 × 10^3^**	6.777 × 10^3^	5.263 × 10^3^	7.452 × 10^3^	5.827 × 10^3^	7.914 × 10^3^	3.621 × 10^3^	7.701 × 10^3^
	Mean	**4.468 × 10^3^**	8.412 × 10^3^	6.443 × 10^3^	8.077 × 10^3^	7.167 × 10^3^	8.652 × 10^3^	5.409 × 10^3^	8.742 × 10^3^
	Std	8.108 × 10^2^	6.718 × 10^2^	3.731 × 10^2^	3.329 × 10^2^	7.517 × 10^2^	**3.003 × 10^2^**	8.430 × 10^2^	5.303 × 10^2^
	P	~	1.734 × 10^−6^	1.734 × 10^−6^	1.734 × 10^−6^	1.921 × 10^−6^	1.734 × 10^−6^	6.156 × 10^−4^	1.734 × 10^−6^
	Rank	1	12	7	11	10	14	2	15
F11	Best	1.194 × 10^3^	4.185 × 10^3^	1.875 × 10^3^	8.567 × 10^3^	2.596 × 10^3^	1.801 × 10^3^	1.409 × 10^3^	7.196 × 10^3^
	Mean	1.319 × 10^3^	6.931 × 10^3^	5.259 × 10^3^	2.261 × 10^4^	6.442 × 10^3^	3.022 × 10^3^	4.254 × 10^3^	1.169 × 10^4^
	Std	6.630 × 10^1^	2.626 × 10^3^	2.098 × 10^3^	8.174 × 10^3^	2.702 × 10^3^	7.265 × 10^2^	3.657 × 10^3^	2.695 × 10^3^
	P	~	1.734 × 10^−6^	1.734 × 10^−6^	1.734 × 10^−6^	1.734 × 10^−6^	1.734 × 10^−6^	1.734 × 10^−6^	1.734 × 10^−6^
	Rank	2	12	10	15	11	6	7	14
F12	Best	1.175 × 10^6^	9.090 × 10^8^	6.760 × 10^7^	1.704 × 10^9^	2.322 × 10^7^	9.207 × 10^8^	8.247 × 10^5^	3.264 × 10^9^
	Mean	1.638 × 10^7^	2.580 × 10^9^	1.187 × 10^8^	3.848 × 10^9^	1.963 × 10^8^	2.200 × 10^9^	3.085 × 10^8^	8.096 × 10^9^
	Std	1.370 × 10^7^	1.287 × 10^9^	2.940 × 10^7^	1.101 × 10^9^	1.368 × 10^8^	6.670 × 10^8^	6.758 × 10^8^	4.159 × 10^9^
	P	~	1.734 × 10^−6^	1.734 × 10^−6^	1.734 × 10^−6^	2.353 × 10^−6^	1.734 × 10^−6^	2.843 × 10^−5^	1.734 × 10^−6^
	Rank	2	11	6	13	7	10	4	14
F13	Best	3.458 × 10^4^	2.042 × 10^8^	9.482 × 10^6^	5.830 × 10^8^	1.497 × 10^5^	3.333 × 10^8^	**9.623 × 10^3^**	9.715 × 10^6^
	Mean	1.576 × 10^5^	9.436 × 10^8^	2.804 × 10^7^	3.090 × 10^9^	1.948 × 10^6^	8.479 × 10^8^	2.867 × 10^8^	2.042 × 10^9^
	Std	1.006 × 10^5^	6.639 × 10^8^	1.246 × 10^7^	1.179 × 10^9^	2.999 × 10^6^	4.076 × 10^8^	9.588 × 10^8^	2.855 × 10^9^
	P	~	1.734 × 10^−6^	1.734 × 10^−6^	1.734 × 10^−6^	2.603 × 10^−6^	1.734 × 10^−6^	3.286 × 10^−1^	1.734 × 10^−6^
	Rank	2	11	7	15	6	12	4	10
F14	Best	**2.008 × 10^3^**	9.713 × 10^4^	4.232 × 10^5^	2.620 × 10^5^	1.455 × 10^5^	8.382 × 10^4^	1.074 × 10^4^	1.140 × 10^5^
	Mean	**3.139 × 10^4^**	1.172 × 10^6^	1.201 × 10^6^	6.236 × 10^6^	2.973 × 10^6^	5.126 × 10^5^	6.220 × 10^5^	3.532 × 10^6^
	Std	**3.300 × 10^4^**	1.832 × 10^6^	9.059 × 10^5^	4.925 × 10^6^	2.980 × 10^6^	4.906 × 10^5^	1.561 × 10^6^	3.854 × 10^6^
	P	~	1.734 × 10^−6^	1.734 × 10^−6^	1.734 × 10^−6^	1.734 × 10^−6^	1.734 × 10^−6^	7.691 × 10^−6^	1.734 × 10^−6^
	Rank	1	9	11	15	12	5	4	14
F15	Best	1.784 × 10^4^	3.436 × 10^7^	1.080 × 10^6^	1.389 × 10^7^	7.407 × 10^4^	2.578 × 10^6^	**2.281 × 10^3^**	3.583 × 10^5^
	Mean	7.126 × 10^4^	1.768 × 10^8^	5.307 × 10^6^	4.163 × 10^8^	7.920 × 10^5^	4.267 × 10^7^	5.005 × 10^4^	3.366 × 10^8^
	Std	3.855 × 10^4^	9.340 × 10^7^	2.768 × 10^6^	2.842 × 10^8^	1.187 × 10^6^	3.585 × 10^7^	3.931 × 10^4^	4.727 × 10^8^
	P	~	1.734 × 10^−6^	1.734 × 10^−6^	1.734 × 10^−6^	1.921 × 10^−6^	1.734 × 10^−6^	8.217 × 10^−3^	1.734 × 10^−6^
	Rank	3	13	10	14	6	11	2	12
F16	Best	**2.191 × 10^3^**	3.343 × 10^3^	2.781 × 10^3^	4.107 × 10^3^	3.137 × 10^3^	3.504 × 10^3^	2.444 × 10^3^	3.701 × 10^3^
	Mean	**2.730 × 10^3^**	3.990 × 10^3^	3.159 × 10^3^	4.773 × 10^3^	3.986 × 10^3^	4.131 × 10^3^	3.237 × 10^3^	5.166 × 10^3^
	Std	3.136 × 10^2^	3.301 × 10^2^	**1.831 × 10^2^**	3.465 × 10^2^	5.266 × 10^2^	2.918 × 10^2^	4.014 × 10^2^	7.622 × 10^2^
	P	~	1.734 × 10^−6^	1.025 × 10^−5^	1.734 × 10^−6^	1.734 × 10^−6^	1.734 × 10^−6^	1.973 × 10^−5^	1.734 × 10^−6^
	Rank	1	11	3	14	10	12	5	15
F17	Best	1.865 × 10^3^	2.574 × 10^3^	2.124 × 10^3^	2.954 × 10^3^	2.038 × 10^3^	2.416 × 10^3^	2.100 × 10^3^	2.356 × 10^3^
	Mean	**2.146 × 10^3^**	2.959 × 10^3^	2.432 × 10^3^	3.543 × 10^3^	2.776 × 10^3^	2.734 × 10^3^	2.594 × 10^3^	3.180 × 10^3^
	Std	1.641 × 10^2^	1.806 × 10^2^	**1.375 × 10^2^**	2.922 × 10^2^	3.417 × 10^2^	1.956 × 10^2^	2.787 × 10^2^	6.667 × 10^2^
	P	~	1.734 × 10^−6^	5.216 × 10^−6^	1.734 × 10^−6^	2.879 × 10^−6^	1.734 × 10^−6^	1.494 × 10^−5^	1.734 × 10^−6^
	Rank	1	13	4	15	10	9	6	12
F18	Best	9.070 × 10^4^	3.164 × 10^6^	6.033 × 10^5^	9.053 × 10^6^	2.724 × 10^5^	2.410 × 10^6^	1.614 × 10^5^	9.945 × 10^5^
	Mean	**5.553 × 10^5^**	1.123 × 10^7^	3.529 × 10^6^	3.646 × 10^7^	7.456 × 10^6^	1.012 × 10^7^	4.999 × 10^6^	3.069 × 10^7^
	Std	**4.482 × 10^5^**	6.724 × 10^6^	2.277 × 10^6^	1.825 × 10^7^	6.873 × 10^6^	7.267 × 10^6^	6.262 × 10^6^	3.776 × 10^7^
	P	~	1.734 × 10^−6^	1.734 × 10^−6^	1.734 × 10^−6^	2.353 × 10^−6^	1.734 × 10^−6^	8.466 × 10^−6^	2.353 × 10^−6^
	Rank	1	12	8	15	9	11	6	13
F19	Best	1.896 × 10^4^	3.871 × 10^7^	8.198 × 10^5^	2.432 × 10^7^	4.654 × 10^5^	2.093 × 10^7^	**5.644 × 10^3^**	2.643 × 10^7^
	Mean	2.241 × 10^6^	3.136 × 10^8^	4.926 × 10^6^	4.366 × 10^8^	1.016 × 10^7^	7.269 × 10^7^	1.354 × 10^7^	4.015 × 10^8^
	Std	1.500 × 10^6^	1.959 × 10^8^	2.874 × 10^6^	2.769 × 10^8^	8.890 × 10^6^	3.411 × 10^7^	3.720 × 10^7^	3.846 × 10^8^
	P	~	1.734 × 10^−6^	5.706 × 10^−4^	1.734 × 10^−6^	3.405 × 10^−5^	1.734 × 10^−6^	2.536 × 10^−1^	1.734 × 10^−6^
	Rank	5	13	7	14	9	11	2	12
F20	Best	2.259 × 10^3^	2.514 × 10^3^	2.360 × 10^3^	2.307 × 10^3^	2.415 × 10^3^	2.564 × 10^3^	**2.213 × 10^3^**	2.535 × 10^3^
	Mean	**2.482 × 10^3^**	2.898 × 10^3^	2.611 × 10^3^	2.755 × 10^3^	2.866 × 10^3^	2.861 × 10^3^	2.793 × 10^3^	3.003 × 10^3^
	Std	1.418 × 10^2^	1.933 × 10^2^	**1.054 × 10^2^**	2.162 × 10^2^	2.266 × 10^2^	1.479 × 10^2^	1.940 × 10^2^	2.064 × 10^2^
	P	~	3.882 × 10^−6^	3.854 × 10^−3^	4.860 × 10^−5^	5.752 × 10^−6^	2.127 × 10^−6^	4.286 × 10^−6^	1.921 × 10^−6^
	Rank	1	12	3	7	10	11	9	14
F21	Best	2.368 × 10^3^	2.613 × 10^3^	2.505 × 10^3^	**2.325 × 10^3^**	2.495 × 10^3^	2.530 × 10^3^	2.452 × 10^3^	2.605 × 10^3^
	Mean	**2.425 × 10^3^**	2.652 × 10^3^	2.537 × 10^3^	2.594 × 10^3^	2.617 × 10^3^	2.594 × 10^3^	2.505 × 10^3^	2.716 × 10^3^
	Std	3.317 × 10^1^	2.331 × 10^1^	**1.412 × 10^1^**	8.132 × 10^1^	5.980 × 10^1^	2.848 × 10^1^	4.665 × 10^1^	6.276 × 10^1^
	P	~	1.734 × 10^−6^	1.734 × 10^−6^	2.603 × 10^−6^	1.734 × 10^−6^	1.734 × 10^−6^	8.466 × 10^−6^	1.734 × 10^−6^
	Rank	1	13	6	9	10	7	4	15
F22	Best	2.304 × 10^3^	4.487 × 10^3^	4.432 × 10^3^	**2.300 × 10^3^**	3.028 × 10^3^	4.121 × 10^3^	2.537 × 10^3^	8.283 × 10^3^
	Mean	**2.409 × 10^3^**	9.137 × 10^3^	6.964 × 10^3^	9.519 × 10^3^	7.971 × 10^3^	9.252 × 10^3^	6.355 × 10^3^	9.784 × 10^3^
	Std	5.176 × 10^2^	1.621 × 10^3^	1.286 × 10^3^	1.979 × 10^3^	1.486 × 10^3^	2.008 × 10^3^	1.384 × 10^3^	6.401 × 10^2^
	P	~	1.734 × 10^−6^	1.734 × 10^−6^	2.353 × 10^−6^	1.734 × 10^−6^	1.734 × 10^−6^	2.353 × 10^−6^	1.734 × 10^−6^
	Rank	1	12	6	15	10	13	5	14
F23	Best	**2.704 × 10^3^**	2.948 × 10^3^	2.775 × 10^3^	2.985 × 10^3^	2.916 × 10^3^	2.976 × 10^3^	2.764 × 10^3^	3.157 × 10^3^
	Mean	**2.786 × 10^3^**	3.118 × 10^3^	2.855 × 10^3^	3.037 × 10^3^	3.069 × 10^3^	3.046 × 10^3^	2.844 × 10^3^	3.447 × 10^3^
	Std	7.747 × 10^1^	8.444 × 10^1^	**1.891 × 10^1^**	2.261 × 10^1^	8.443 × 10^1^	3.558 × 10^1^	5.132 × 10^1^	1.626 × 10^2^
	P	~	1.734 × 10^−6^	5.307 × 10^−5^	1.921 × 10^−6^	1.734 × 10^−6^	1.921 × 10^−6^	4.196 × 10^−4^	1.734 × 10^−6^
	Rank	1	10	3	7	9	8	2	14
F24	Best	**2.872 × 10^3^**	3.111 × 10^3^	3.039 × 10^3^	3.129 × 10^3^	3.047 × 10^3^	3.151 × 10^3^	2.940 × 10^3^	3.311 × 10^3^
	Mean	**2.975 × 10^3^**	3.260 × 10^3^	3.070 × 10^3^	3.164 × 10^3^	3.257 × 10^3^	3.221 × 10^3^	2.991 × 10^3^	3.649 × 10^3^
	Std	1.150 × 10^2^	1.040 × 10^2^	**1.375 × 10^1^**	2.128 × 10^1^	1.169 × 10^2^	3.766 × 10^1^	3.363 × 10^1^	2.672 × 10^2^
	P	~	3.882 × 10^−6^	1.150 × 10^−4^	8.466 × 10^−6^	4.729 × 10^−6^	3.515 × 10^−6^	3.327 × 10^−2^	1.734 × 10^−6^
	Rank	1	9	4	7	10	8	2	14
F25	Best	**2.887 × 10^3^**	4.666 × 10^3^	3.148 × 10^3^	2.888 × 10^3^	2.995 × 10^3^	3.253 × 10^3^	2.897 × 10^3^	3.740 × 10^3^
	Mean	2.907 × 10^3^	5.650 × 10^3^	3.283 × 10^3^	2.993 × 10^3^	3.092 × 10^3^	3.447 × 10^3^	3.208 × 10^3^	4.552 × 10^3^
	Std	2.278 × 10^1^	5.980 × 10^2^	6.683 × 10^1^	2.208 × 10^2^	5.320 × 10^1^	1.279 × 10^2^	3.631 × 10^2^	4.811 × 10^2^
	P	~	1.734 × 10^−6^	1.734 × 10^−6^	4.779 × 10^−1^	1.734 × 10^−6^	1.734 × 10^−6^	3.515 × 10^−6^	1.734 × 10^−6^
	Rank	1	15	9	3	6	11	7	13
F26	Best	**2.831 × 10^3^**	5.585 × 10^3^	5.462 × 10^3^	6.942 × 10^3^	3.448 × 10^3^	6.742 × 10^3^	5.150 × 10^3^	8.515 × 10^3^
	Mean	**3.867 × 10^3^**	7.873 × 10^3^	5.893 × 10^3^	8.015 × 10^3^	8.336 × 10^3^	7.709 × 10^3^	5.993 × 10^3^	1.082 × 10^4^
	Std	1.146 × 10^3^	8.115 × 10^2^	**1.678 × 10^2^**	4.551 × 10^2^	1.489 × 10^3^	4.471 × 10^2^	5.281 × 10^2^	1.296 × 10^3^
	P	~	1.734 × 10^−6^	3.882 × 10^−6^	1.734 × 10^−6^	1.921 × 10^−6^	1.734 × 10^−6^	5.216 × 10^−6^	1.734 × 10^−6^
	Rank	1	8	3	9	10	7	4	14
F27	Best	**3.198 × 10^3^**	3.242 × 10^3^	3.227 × 10^3^	3.328 × 10^3^	3.276 × 10^3^	3.395 × 10^3^	3.219 × 10^3^	3.450 × 10^3^
	Mean	3.239 × 10^3^	3.377 × 10^3^	**3.238 × 10^3^**	3.385 × 10^3^	3.439 × 10^3^	3.484 × 10^3^	3.255 × 10^3^	4.081 × 10^3^
	Std	3.317 × 10^1^	1.332 × 10^2^	**4.830 × 10^0^**	3.359 × 10^1^	1.091 × 10^2^	5.334 × 10^1^	2.478 × 10^1^	3.845 × 10^2^
	P	~	2.879 × 10^−6^	3.493 × 10^−1^	1.734 × 10^−6^	1.734 × 10^−6^	1.734 × 10^−6^	4.992 × 10^−3^	1.734 × 10^−6^
	Rank	1	6	2	8	9	10	3	15
F28	Best	3.216 × 10^3^	4.005 × 10^3^	3.599 × 10^3^	4.520 × 10^3^	3.326 × 10^3^	3.701 × 10^3^	3.250 × 10^3^	4.875 × 10^3^
	Mean	3.247 × 10^3^	4.971 × 10^3^	3.947 × 10^3^	6.530 × 10^3^	3.478 × 10^3^	4.172 × 10^3^	4.330 × 10^3^	6.552 × 10^3^
	Std	2.633 × 10^1^	7.610 × 10^2^	2.161 × 10^2^	7.088 × 10^2^	9.188 × 10^1^	2.722 × 10^2^	9.926 × 10^2^	9.030 × 10^2^
	P	~	1.734 × 10^−6^	1.734 × 10^−6^	1.734 × 10^−6^	1.734 × 10^−6^	1.734 × 10^−6^	1.734 × 10^−6^	1.734 × 10^−6^
	Rank	2	12	7	14	4	10	8	13
F29	Best	3.639 × 10^3^	4.207 × 10^3^	3.842 × 10^3^	4.908 × 10^3^	4.222 × 10^3^	4.618 × 10^3^	**3.588 × 10^3^**	5.163 × 10^3^
	Mean	**3.964 × 10^3^**	4.869 × 10^3^	4.154 × 10^3^	5.713 × 10^3^	5.160 × 10^3^	5.089 × 10^3^	4.260 × 10^3^	6.940 × 10^3^
	Std	1.973 × 10^2^	4.020 × 10^2^	**1.416 × 10^2^**	3.688 × 10^2^	6.096 × 10^2^	2.774 × 10^2^	3.150 × 10^2^	1.045 × 10^3^
	P	~	1.734 × 10^−6^	8.307 × 10^−4^	1.734 × 10^−6^	1.921 × 10^−6^	1.734 × 10^−6^	2.613 × 10^−4^	1.734 × 10^−6^
	Rank	1	7	2	13	9	11	4	15
F30	Best	4.997 × 10^5^	2.547 × 10^7^	4.077 × 10^5^	1.388 × 10^7^	1.213 × 10^6^	7.451 × 10^7^	**9.665 × 10^3^**	7.595 × 10^6^
	Mean	5.431 × 10^6^	1.279 × 10^8^	1.760 × 10^6^	4.782 × 10^8^	3.445 × 10^7^	1.692 × 10^8^	1.143 × 10^7^	4.151 × 10^8^
	Std	3.953 × 10^6^	7.272 × 10^7^	1.426 × 10^6^	3.918 × 10^8^	3.754 × 10^7^	5.537 × 10^7^	5.225 × 10^7^	5.875 × 10^8^
	P	~	1.734 × 10^−6^	5.792 × 10^−5^	1.734 × 10^−6^	1.127 × 10^−5^	1.734 × 10^−6^	3.162 × 10^−3^	1.734 × 10^−6^
	Rank	4	11	3	15	8	14	2	13
Mean Rank		**1.6**	11.7	6.2	10	9	9.8	5	13.5
+/=/−		~	29/0/0	26/1/2	26/2/1	29/0/0	29/0/0	28/0/1	29/0/0

**Table A11:** Comparison results with newly proposed algorithms on CEC2017 (Dim = 30).

Function	Index	MShOA	GJO	WAA	SHO	HO	PO	AO
F1	Best	2.338 × 10^10^	5.648 × 10^9^	8.972 × 10^5^	1.728 × 10^10^	1.152 × 10^10^	7.342 × 10^8^	7.351 × 10^7^
	Mean	5.225 × 10^10^	1.313 × 10^10^	**1.944 × 10^6^**	2.752 × 10^10^	1.601 × 10^10^	3.951 × 10^9^	2.115 × 10^8^
	Std	1.611 × 10^10^	4.324 × 10^9^	**4.066 × 10^5^**	6.121 × 10^9^	2.743 × 10^9^	2.513 × 10^9^	1.021 × 10^8^
	P	1.734 × 10^−6^	1.734 × 10^−6^	5.287 × 10^−4^	1.734 × 10^−6^	1.734 × 10^−6^	1.734 × 10^−6^	5.307 × 10^−5^
	Rank	15	8	2	12	10	7	4
F3	Best	6.173 × 10^4^	3.525 × 10^4^	8.833 × 10^3^	5.739 × 10^4^	5.790 × 10^4^	2.398 × 10^4^	3.775 × 10^4^
	Mean	8.865 × 10^4^	5.245 × 10^4^	1.369 × 10^4^	7.157 × 10^4^	6.668 × 10^4^	3.571 × 10^4^	5.323 × 10^4^
	Std	9.495 × 10^3^	7.615 × 10^3^	3.742 × 10^3^	6.389 × 10^3^	4.921 × 10^3^	8.258 × 10^3^	8.265 × 10^3^
	P	1.734 × 10^−6^	1.734 × 10^−6^	1.734 × 10^−6^	1.734 × 10^−6^	1.734 × 10^−6^	1.734 × 10^−6^	1.734 × 10^−6^
	Rank	9	4	2	8	7	3	5
F4	Best	5.698 × 10^3^	7.360 × 10^2^	**4.744 × 10^2^**	1.612 × 10^3^	1.439 × 10^3^	5.710 × 10^2^	5.321 × 10^2^
	Mean	1.522 × 10^4^	1.285 × 10^3^	5.117 × 10^2^	4.096 × 10^3^	2.327 × 10^3^	7.233 × 10^2^	5.985 × 10^2^
	Std	6.862 × 10^3^	5.185 × 10^2^	**8.830 × 10^0^**	1.812 × 10^3^	7.107 × 10^2^	1.829 × 10^2^	5.157 × 10^1^
	P	1.734 × 10^−6^	1.734 × 10^−6^	5.984 × 10^−2^	1.734 × 10^−6^	1.734 × 10^−6^	1.734 × 10^−6^	5.216 × 10^−6^
	Rank	15	8	2	13	9	4	3
F5	Best	8.312 × 10^2^	6.215 × 10^2^	7.729 × 10^2^	7.607 × 10^2^	7.830 × 10^2^	6.955 × 10^2^	6.448 × 10^2^
	Mean	9.002 × 10^2^	7.092 × 10^2^	8.162 × 10^2^	8.250 × 10^2^	8.420 × 10^2^	7.586 × 10^2^	6.969 × 10^2^
	Std	4.098 × 10^1^	4.877 × 10^1^	2.033 × 10^1^	3.401 × 10^1^	2.678 × 10^1^	2.960 × 10^1^	3.457 × 10^1^
	P	1.734 × 10^−6^	1.921 × 10^−6^	1.734 × 10^−6^	1.734 × 10^−6^	1.734 × 10^−6^	1.734 × 10^−6^	2.127 × 10^−6^
	Rank	15	4	8	11	12	7	2
F6	Best	6.719 × 10^2^	6.200 × 10^2^	6.640 × 10^2^	6.572 × 10^2^	6.571 × 10^2^	6.509 × 10^2^	6.349 × 10^2^
	Mean	6.871 × 10^2^	6.370 × 10^2^	6.686 × 10^2^	6.745 × 10^2^	6.730 × 10^2^	6.630 × 10^2^	6.476 × 10^2^
	Std	9.805 × 10^0^	6.951 × 10^0^	4.794 × 10^0^	7.321 × 10^0^	6.815 × 10^0^	7.307 × 10^0^	7.041 × 10^0^
	P	1.734 × 10^−6^	5.984 × 10^−2^	1.734 × 10^−6^	1.734 × 10^−6^	1.734 × 10^−6^	1.734 × 10^−6^	1.251 × 10^−4^
	Rank	15	3	9	13	12	8	5
F7	Best	1.321 × 10^3^	9.741 × 10^2^	1.274 × 10^3^	1.169 × 10^3^	1.184 × 10^3^	1.018 × 10^3^	9.816 × 10^2^
	Mean	1.436 × 10^3^	1.051 × 10^3^	1.354 × 10^3^	1.265 × 10^3^	1.316 × 10^3^	1.199 × 10^3^	1.085 × 10^3^
	Std	6.372 × 10^1^	4.974 × 10^1^	3.080 × 10^1^	5.456 × 10^1^	5.682 × 10^1^	8.625 × 10^1^	5.861 × 10^1^
	P	1.734 × 10^−6^	1.734 × 10^−6^	1.734 × 10^−6^	1.734 × 10^−6^	1.734 × 10^−6^	1.734 × 10^−6^	1.734 × 10^−6^
	Rank	13	3	12	9	11	8	4
F8	Best	9.871 × 10^2^	8.984 × 10^2^	9.907 × 10^2^	1.026 × 10^3^	9.964 × 10^2^	9.448 × 10^2^	8.782 × 10^2^
	Mean	1.133 × 10^3^	9.704 × 10^2^	1.020 × 10^3^	1.054 × 10^3^	1.055 × 10^3^	1.006 × 10^3^	9.498 × 10^2^
	Std	3.817 × 10^1^	2.936 × 10^1^	**1.496 × 10^1^**	1.610 × 10^1^	2.596 × 10^1^	3.963 × 10^1^	2.704 × 10^1^
	P	1.734 × 10^−6^	1.057 × 10^−4^	1.921 × 10^−6^	1.734 × 10^−6^	1.734 × 10^−6^	1.025 × 10^−5^	1.306 × 10^−1^
	Rank	14	3	7	9	10	6	2
F9	Best	8.597 × 10^3^	1.896 × 10^3^	6.032 × 10^3^	5.282 × 10^3^	4.979 × 10^3^	3.701 × 10^3^	3.946 × 10^3^
	Mean	1.145 × 10^4^	4.766 × 10^3^	6.755 × 10^3^	7.406 × 10^3^	7.378 × 10^3^	6.248 × 10^3^	6.317 × 10^3^
	Std	1.603 × 10^3^	1.496 × 10^3^	5.576 × 10^2^	8.227 × 10^2^	8.956 × 10^2^	1.258 × 10^3^	1.324 × 10^3^
	P	1.734 × 10^−6^	4.390 × 10^−3^	2.603 × 10^−6^	1.734 × 10^−6^	1.921 × 10^−6^	1.799 × 10^−5^	6.984 × 10^−6^
	Rank	14	3	6	9	8	4	5
F10	Best	7.245 × 10^3^	4.481 × 10^3^	4.662 × 10^3^	5.822 × 10^3^	5.709 × 10^3^	4.132 × 10^3^	4.546 × 10^3^
	Mean	8.574 × 10^3^	6.082 × 10^3^	5.965 × 10^3^	7.062 × 10^3^	6.718 × 10^3^	6.289 × 10^3^	6.011 × 10^3^
	Std	7.066 × 10^2^	1.304 × 10^3^	6.056 × 10^2^	6.680 × 10^2^	5.336 × 10^2^	7.494 × 10^2^	7.280 × 10^2^
	P	1.734 × 10^−6^	2.163 × 10^−5^	3.515 × 10^−6^	1.734 × 10^−6^	1.734 × 10^−6^	2.353 × 10^−6^	4.286 × 10^−6^
	Rank	13	5	3	9	8	6	4
F11	Best	4.347 × 10^3^	1.406 × 10^3^	**1.153 × 10^3^**	2.480 × 10^3^	2.174 × 10^3^	1.323 × 10^3^	1.550 × 10^3^
	Mean	8.118 × 10^3^	3.216 × 10^3^	**1.196 × 10^3^**	4.177 × 10^3^	3.825 × 10^3^	1.583 × 10^3^	1.994 × 10^3^
	Std	2.409 × 10^3^	1.363 × 10^3^	**4.115 × 10^1^**	1.240 × 10^3^	1.101 × 10^3^	2.195 × 10^2^	4.446 × 10^2^
	P	1.734 × 10^−6^	1.734 × 10^−6^	3.515 × 10^−6^	1.734 × 10^−6^	1.734 × 10^−6^	1.921 × 10^−6^	1.734 × 10^−6^
	Rank	13	5	1	9	8	3	4
F12	Best	3.194 × 10^9^	1.445 × 10^7^	**5.978 × 10^5^**	1.104 × 10^9^	2.193 × 10^8^	1.230 × 10^7^	1.266 × 10^7^
	Mean	1.108 × 10^10^	1.010 × 10^9^	**3.464 × 10^6^**	4.046 × 10^9^	9.799 × 10^8^	1.764 × 10^8^	6.576 × 10^7^
	Std	5.611 × 10^9^	9.926 × 10^8^	**1.358 × 10^6^**	2.467 × 10^9^	6.731 × 10^8^	1.641 × 10^8^	5.225 × 10^7^
	P	1.734 × 10^−6^	1.921 × 10^−6^	4.449 × 10^−5^	1.734 × 10^−6^	1.734 × 10^−6^	1.734 × 10^−6^	1.494 × 10^−5^
	Rank	15	8	1	12	9	5	3
F13	Best	1.297 × 10^8^	1.498 × 10^5^	3.340 × 10^4^	2.863 × 10^7^	9.169 × 10^6^	5.747 × 10^4^	3.276 × 10^5^
	Mean	6.769 × 10^9^	2.663 × 10^8^	**1.306 × 10^5^**	1.834 × 10^9^	2.379 × 10^8^	4.796 × 10^5^	9.350 × 10^5^
	Std	8.962 × 10^9^	7.270 × 10^8^	**6.214 × 10^4^**	2.183 × 10^9^	2.332 × 10^8^	1.091 × 10^6^	7.500 × 10^5^
	P	1.734 × 10^−6^	1.734 × 10^−6^	1.714 × 10^−1^	1.734 × 10^−6^	1.734 × 10^−6^	2.703 × 10^−2^	1.734 × 10^−6^
	Rank	14	8	1	13	9	3	5
F14	Best	1.637 × 10^5^	1.743 × 10^4^	5.331 × 10^3^	1.850 × 10^5^	8.676 × 10^4^	1.184 × 10^4^	1.769 × 10^4^
	Mean	2.473 × 10^6^	8.012 × 10^5^	1.375 × 10^5^	8.705 × 10^5^	1.099 × 10^6^	2.380 × 10^5^	7.041 × 10^5^
	Std	2.397 × 10^6^	8.231 × 10^5^	1.344 × 10^5^	6.351 × 10^5^	7.565 × 10^5^	2.513 × 10^5^	7.533 × 10^5^
	P	1.734 × 10^−6^	1.921 × 10^−6^	1.639 × 10^−5^	1.734 × 10^−6^	1.734 × 10^−6^	1.639 × 10^−5^	2.353 × 10^−6^
	Rank	13	7	2	8	10	3	6
F15	Best	3.754 × 10^6^	3.965 × 10^4^	1.426 × 10^4^	1.428 × 10^4^	2.775 × 10^4^	2.557 × 10^4^	2.557 × 10^4^
	Mean	4.842 × 10^8^	4.952 × 10^6^	**2.992 × 10^4^**	4.261 × 10^6^	2.527 × 10^6^	1.270 × 10^5^	1.406 × 10^5^
	Std	2.681 × 10^8^	8.888 × 10^6^	**2.277 × 10^4^**	8.667 × 10^6^	3.406 × 10^6^	1.664 × 10^5^	9.196 × 10^4^
	P	1.734 × 10^−6^	1.639 × 10^−5^	9.711 × 10^−5^	3.882 × 10^−6^	1.025 × 10^−5^	3.160 × 10^−2^	2.831 × 10^−4^
	Rank	15	8	1	9	7	4	5
F16	Best	3.409 × 10^3^	2.414 × 10^3^	2.973 × 10^3^	2.858 × 10^3^	3.066 × 10^3^	2.641 × 10^3^	2.311 × 10^3^
	Mean	4.341 × 10^3^	3.047 × 10^3^	3.706 × 10^3^	3.743 × 10^3^	3.950 × 10^3^	3.441 × 10^3^	3.134 × 10^3^
	Std	4.840 × 10^2^	3.768 × 10^2^	4.259 × 10^2^	3.617 × 10^2^	5.376 × 10^2^	4.302 × 10^2^	3.458 × 10^2^
	P	1.921 × 10^−6^	1.114 × 10^−3^	1.734 × 10^−6^	1.734 × 10^−6^	1.921 × 10^−6^	1.734 × 10^−6^	1.891 × 10^−4^
	Rank	13	2	8	7	9	6	4
F17	Best	2.484 × 10^3^	**1.790 × 10^3^**	2.345 × 10^3^	2.006 × 10^3^	2.149 × 10^3^	1.926 × 10^3^	1.877 × 10^3^
	Mean	5.692 × 10^3^	2.275 × 10^3^	2.960 × 10^3^	2.706 × 10^3^	2.690 × 10^3^	2.510 × 10^3^	2.322 × 10^3^
	Std	1.184 × 10^4^	2.646 × 10^2^	3.428 × 10^2^	7.824 × 10^2^	2.989 × 10^2^	2.606 × 10^2^	2.451 × 10^2^
	P	1.734 × 10^−6^	4.716 × 10^−2^	1.734 × 10^−6^	1.799 × 10^−5^	2.127 × 10^−6^	1.127 × 10^−5^	3.589 × 10^−4^
	Rank	14	2	11	7	8	5	3
F18	Best	1.882 × 10^6^	6.210 × 10^4^	9.653 × 10^4^	5.974 × 10^5^	4.641 × 10^5^	**5.772 × 10^4^**	9.201 × 10^4^
	Mean	3.894 × 10^7^	2.498 × 10^6^	1.613 × 10^6^	4.278 × 10^6^	9.665 × 10^6^	2.124 × 10^6^	3.457 × 10^6^
	Std	4.407 × 10^7^	4.731 × 10^6^	1.168 × 10^6^	3.754 × 10^6^	1.066 × 10^7^	2.199 × 10^6^	3.313 × 10^6^
	P	1.921 × 10^−6^	1.957 × 10^−2^	6.639 × 10^−4^	8.466 × 10^−6^	3.515 × 10^−6^	1.251 × 10^−4^	3.882 × 10^−6^
	Rank	2	3	7	10	4	5	2
F19	Best	8.121 × 10^6^	8.571 × 10^4^	9.760 × 10^4^	4.327 × 10^4^	1.428 × 10^5^	1.061 × 10^5^	3.262 × 10^5^
	Mean	1.175 × 10^9^	1.192 × 10^7^	**2.036 × 10^5^**	5.932 × 10^7^	1.361 × 10^7^	2.199 × 10^6^	2.160 × 10^6^
	Std	1.466 × 10^9^	2.570 × 10^7^	**7.376 × 10^4^**	9.232 × 10^7^	1.064 × 10^7^	1.780 × 10^6^	1.590 × 10^6^
	P	1.734 × 10^−6^	4.950 × 10^−2^	2.879 × 10^−6^	8.730 × 10^−3^	4.286 × 10^−6^	7.655 × 10^−1^	8.612 × 10^−1^
	Rank	6	1	8	10	3	4	6
F20	Best	2.533 × 10^3^	2.299 × 10^3^	2.604 × 10^3^	2.340 × 10^3^	2.471 × 10^3^	2.295 × 10^3^	2.224 × 10^3^
	Mean	2.960 × 10^3^	2.533 × 10^3^	3.078 × 10^3^	2.612 × 10^3^	2.793 × 10^3^	2.642 × 10^3^	2.584 × 10^3^
	Std	2.021 × 10^2^	2.059 × 10^2^	2.163 × 10^2^	1.780 × 10^2^	1.727 × 10^2^	1.882 × 10^2^	1.795 × 10^2^
	P	2.603 × 10^−6^	3.709 × 10^−1^	1.921 × 10^−6^	9.842 × 10^−3^	2.879 × 10^−6^	4.897 × 10^−4^	1.957 × 10^−2^
	Rank	2	15	5	8	6	4	2
F21	Best	2.608 × 10^3^	2.417 × 10^3^	2.569 × 10^3^	2.548 × 10^3^	2.569 × 10^3^	2.430 × 10^3^	2.384 × 10^3^
	Mean	2.674 × 10^3^	2.477 × 10^3^	2.667 × 10^3^	2.598 × 10^3^	2.649 × 10^3^	2.528 × 10^3^	2.468 × 10^3^
	Std	5.280 × 10^1^	3.626 × 10^1^	6.368 × 10^1^	3.034 × 10^1^	5.169 × 10^1^	4.906 × 10^1^	3.717 × 10^1^
	P	1.734 × 10^−6^	4.072 × 10^−5^	1.734 × 10^−6^	1.734 × 10^−6^	1.734 × 10^−6^	4.286 × 10^−6^	2.225 × 10^−4^
	Rank	3	12	8	11	5	2	3
F22	Best	5.601 × 10^3^	3.442 × 10^3^	6.306 × 10^3^	4.458 × 10^3^	3.686 × 10^3^	2.716 × 10^3^	2.355 × 10^3^
	Mean	8.983 × 10^3^	5.972 × 10^3^	7.944 × 10^3^	6.952 × 10^3^	7.156 × 10^3^	4.447 × 10^3^	2.424 × 10^3^
	Std	1.288 × 10^3^	2.103 × 10^3^	8.753 × 10^2^	1.538 × 10^3^	1.839 × 10^3^	2.024 × 10^3^	**6.696 × 10^1^**
	P	1.734 × 10^−6^	2.879 × 10^−6^	1.734 × 10^−6^	1.734 × 10^−6^	1.734 × 10^−6^	1.734 × 10^−6^	3.112 × 10^−5^
	Rank	4	9	7	8	3	2	4
F23	Best	3.062 × 10^3^	2.824 × 10^3^	3.162 × 10^3^	3.022 × 10^3^	3.046 × 10^3^	2.883 × 10^3^	2.762 × 10^3^
	Mean	3.276 × 10^3^	2.924 × 10^3^	3.501 × 10^3^	3.181 × 10^3^	3.200 × 10^3^	3.015 × 10^3^	2.862 × 10^3^
	Std	1.161 × 10^2^	6.533 × 10^1^	2.311 × 10^2^	7.319 × 10^1^	7.797 × 10^1^	6.007 × 10^1^	4.739 × 10^1^
	P	1.734 × 10^−6^	3.882 × 10^−6^	1.734 × 10^−6^	1.734 × 10^−6^	1.921 × 10^−6^	1.921 × 10^−6^	1.057 × 10^−4^
	Rank	5	15	11	12	6	4	5
F24	Best	3.248 × 10^3^	2.979 × 10^3^	3.373 × 10^3^	3.274 × 10^3^	3.195 × 10^3^	2.989 × 10^3^	2.958 × 10^3^
	Mean	3.516 × 10^3^	3.093 × 10^3^	3.696 × 10^3^	3.441 × 10^3^	3.393 × 10^3^	3.133 × 10^3^	3.021 × 10^3^
	Std	1.607 × 10^2^	5.599 × 10^1^	2.422 × 10^2^	1.097 × 10^2^	1.146 × 10^2^	8.140 × 10^1^	4.027 × 10^1^
	P	1.921 × 10^−6^	1.150 × 10^−4^	1.734 × 10^−6^	1.734 × 10^−6^	1.734 × 10^−6^	2.163 × 10^−5^	8.730 × 10^−3^
	Rank	5	15	12	11	6	3	5
F25	Best	4.073 × 10^3^	2.988 × 10^3^	2.889 × 10^3^	3.213 × 10^3^	3.150 × 10^3^	2.997 × 10^3^	2.908 × 10^3^
	Mean	5.393 × 10^3^	3.182 × 10^3^	**2.900 × 10^3^**	3.660 × 10^3^	3.359 × 10^3^	3.059 × 10^3^	2.980 × 10^3^
	Std	1.177 × 10^3^	1.437 × 10^2^	**5.383 × 10^0^**	3.317 × 10^2^	1.412 × 10^2^	3.825 × 10^1^	3.693 × 10^1^
	P	1.734 × 10^−6^	1.734 × 10^−6^	2.304 × 10^−2^	1.734 × 10^−6^	1.734 × 10^−6^	1.734 × 10^−6^	1.734 × 10^−6^
	Rank	8	2	12	10	5	4	8
F26	Best	8.733 × 10^3^	5.445 × 10^3^	3.581 × 10^3^	7.214 × 10^3^	5.660 × 10^3^	4.565 × 10^3^	3.026 × 10^3^
	Mean	1.095 × 10^4^	6.155 × 10^3^	9.315 × 10^3^	8.558 × 10^3^	8.555 × 10^3^	7.488 × 10^3^	4.348 × 10^3^
	Std	1.537 × 10^3^	4.450 × 10^2^	1.586 × 10^3^	5.424 × 10^2^	1.141 × 10^3^	1.124 × 10^3^	1.112 × 10^3^
	P	1.734 × 10^−6^	2.127 × 10^−6^	1.734 × 10^−6^	1.734 × 10^−6^	1.921 × 10^−6^	2.127 × 10^−6^	2.210 × 10^−1^
	Rank	5	13	12	11	6	2	5
F27	Best	3.278 × 10^3^	3.402 × 10^3^	3.481 × 10^3^	3.326 × 10^3^	3.241 × 10^3^	3.251 × 10^3^	3.278 × 10^3^
	Mean	3.983 × 10^3^	3.384 × 10^3^	4.055 × 10^3^	3.737 × 10^3^	3.547 × 10^3^	3.342 × 10^3^	3.303 × 10^3^
	Std	2.616 × 10^2^	8.456 × 10^1^	4.120 × 10^2^	1.586 × 10^2^	1.207 × 10^2^	7.456 × 10^1^	3.736 × 10^1^
	P	1.734 × 10^−6^	2.353 × 10^−6^	1.734 × 10^−6^	1.734 × 10^−6^	1.734 × 10^−6^	2.127 × 10^−6^	2.597 × 10^−5^
	Rank	7	13	12	11	5	4	7
F28	Best	3.443 × 10^3^	**3.198 × 10^3^**	3.931 × 10^3^	3.666 × 10^3^	3.367 × 10^3^	3.322 × 10^3^	3.443 × 10^3^
	Mean	7.471 × 10^3^	3.999 × 10^3^	**3.216 × 10^3^**	4.710 × 10^3^	4.231 × 10^3^	3.544 × 10^3^	3.392 × 10^3^
	Std	1.074 × 10^3^	5.262 × 10^2^	**1.936 × 10^1^**	4.937 × 10^2^	3.433 × 10^2^	1.272 × 10^2^	5.752 × 10^1^
	P	1.734 × 10^−6^	1.734 × 10^−6^	8.919 × 10^−5^	1.734 × 10^−6^	1.734 × 10^−6^	1.734 × 10^−6^	1.734 × 10^−6^
	Rank	6	1	11	9	5	3	6
F29	Best	3.594 × 10^3^	4.499 × 10^3^	4.546 × 10^3^	4.470 × 10^3^	4.046 × 10^3^	3.728 × 10^3^	3.594 × 10^3^
	Mean	6.837 × 10^3^	4.214 × 10^3^	5.014 × 10^3^	5.116 × 10^3^	5.592 × 10^3^	4.838 × 10^3^	4.300 × 10^3^
	Std	2.860 × 10^3^	2.625 × 10^2^	3.402 × 10^2^	2.954 × 10^2^	6.130 × 10^2^	4.372 × 10^2^	2.506 × 10^2^
	P	1.734 × 10^−6^	2.613 × 10^−4^	1.734 × 10^−6^	1.734 × 10^−6^	1.734 × 10^−6^	2.603 × 10^−6^	5.792 × 10^−5^
	Rank	3	8	10	12	6	5	3
F30	Best	5.061 × 10^6^	2.091 × 10^5^	6.767 × 10^6^	5.194 × 10^6^	3.833 × 10^6^	1.514 × 10^6^	5.061 × 10^6^
	Mean	3.969 × 10^8^	3.614 × 10^7^	**1.379 × 10^6^**	1.381 × 10^8^	8.020 × 10^7^	1.840 × 10^7^	1.600 × 10^7^
	Std	7.391 × 10^8^	3.454 × 10^7^	**8.652 × 10^5^**	2.551 × 10^8^	7.189 × 10^7^	1.350 × 10^7^	1.260 × 10^7^
	P	1.734 × 10^−6^	3.882 × 10^−6^	8.466 × 10^−6^	1.734 × 10^−6^	1.921 × 10^−6^	3.112 × 10^−5^	6.320 × 10^−5^
	Rank	7	1	10	9	6	5	7
Mean Rank		13.7	5	6.3	9.8	9.6	5.1	3.8
+/=/−		29/0/0	27/2/0	20/1/8	29/0/0	29/0/0	28/1/0	28/1/0

**Table A12:** Comparison results with classic highly-cited algorithms on CEC2017 (Dim = 50).

Function	Index	EAO	PSO	DE	CMA-ES	WOA	SCA	MFO	HHO
F1	Best	1.527 × 10^6^	6.705 × 10^10^	1.696 × 10^10^	**3.542 × 10^2^**	8.694 × 10^8^	4.350 × 10^10^	3.011 × 10^9^	7.825 × 10^10^
	Mean	3.204 × 10^7^	9.310 × 10^10^	2.219 × 10^10^	1.083 × 10^10^	2.058 × 10^9^	5.583 × 10^10^	3.605 × 10^10^	1.037 × 10^11^
	Std	3.821 × 10^7^	1.547 × 10^10^	3.109 × 10^9^	2.215 × 10^10^	8.962 × 10^8^	6.887 × 10^9^	1.869 × 10^10^	1.185 × 10^10^
	P	~	1.734 × 10^−6^	1.734 × 10^−6^	1.650 × 10^−1^	1.734 × 10^−6^	1.734 × 10^−6^	1.734 × 10^−6^	1.734 × 10^−6^
	Rank	2	13	7	3	5	11	8	14
F3	Best	**1.014 × 10^4^**	2.676 × 10^5^	2.908 × 10^5^	2.530 × 10^5^	1.498 × 10^5^	1.087 × 10^5^	1.826 × 10^5^	1.415 × 10^5^
	Mean	**2.725 × 10^4^**	3.983 × 10^5^	3.998 × 10^5^	3.524 × 10^5^	2.368 × 10^5^	1.544 × 10^5^	3.583 × 10^5^	2.120 × 10^5^
	Std	**9.339 × 10^3^**	7.708 × 10^4^	4.655 × 10^4^	5.097 × 10^4^	8.873 × 10^4^	2.730 × 10^4^	8.362 × 10^4^	3.864 × 10^4^
	P	~	1.734 ×10^−6^	1.734 ×10^−6^	1.734 ×10^−6^	1.734 ×10^−6^	1.734 ×10^−6^	1.734 ×10^−6^	1.734 × 10^−6^
	Rank	1	14	15	13	11	6	12	10
F4	Best	**4.880 × 10^2^**	6.240 × 10^3^	1.639 × 10^3^	3.064 × 10^3^	9.203 × 10^2^	6.563 × 10^3^	1.205 × 10^3^	1.761 × 10^4^
	Mean	5.911 × 10^2^	1.266 × 10^4^	2.041 × 10^3^	7.715 × 10^3^	1.344 × 10^3^	1.006 × 10^4^	4.586 × 10^3^	2.908 × 10^4^
	Std	4.194 × 10^1^	3.814 × 10^3^	2.303 × 10^2^	2.240 × 10^3^	2.153 × 10^2^	2.154 × 10^3^	3.269 × 10^3^	7.041 × 10^3^
	P	~	1.734 ×10 ^−6^	1.734 ×10 ^−6^	1.734 ×10 ^−6^	1.734 ×10 ^−6^	1.734 ×10 ^−6^	1.734 ×10 ^−6^	1.734 × 10^−6^
	Rank	2	12	6	10	4	11	7	14
F5	Best	6.567 × 10^2^	1.115 × 10^3^	9.938 × 10^2^	**5.338 × 10^2^**	8.712 × 10^2^	9.975 × 10^2^	8.113 × 10^2^	1.103 × 10^3^
	Mean	7.654 × 10^2^	1.224 × 10^3^	1.039 × 10^3^	**5.650 × 10^2^**	1.016 × 10^3^	1.100 × 10^3^	9.949 × 10^2^	1.172 × 10^3^
	Std	7.451 × 10^1^	5.787 × 10^1^	2.300 × 10^1^	1.097 × 10^2^	7.595 × 10^1^	4.369 × 10^1^	9.100 × 10^1^	3.278 × 10^1^
	P	~	1.734 ×10^−6^	1.734 ×10^−6^	1.734 ×10^−6^	1.734 ×10^−6^	1.734 ×10^−6^	1.734 ×10^−6^	1.734 × 10^−6^
	Rank	2	15	10	1	8	12	7	14
F6	Best	6.237 × 10^2^	6.774 × 10^2^	6.284 × 10^2^	**6.000 × 10^2^**	6.683 × 10^2^	6.683 × 10^2^	6.412 × 10^2^	6.830 × 10^2^
	Mean	6.428 × 10^2^	6.927 × 10^2^	**6.350 × 10^2^**	6.445 × 10^2^	6.889 × 10^2^	6.796 × 10^2^	6.570 × 10^2^	7.002 × 10^2^
	Std	1.376 × 10^1^	7.499 × 10^0^	2.205 × 10^0^	3.250 × 10^1^	1.329 × 10^1^	7.317 × 10^0^	9.545 × 10^0^	7.265 × 10^0^
	P	~	1.734 × 10^−6^	9.271 × 10^−3^	8.774 × 10^−1^	1.734 × 10^−6^	1.734 × 10^−6^	1.891 × 10^−4^	1.734 × 10^−6^
	Rank	2	13	1	4	11	9	5	15
F7	Best	1.006 × 10^3^	3.067 × 10^3^	1.778 × 10^3^	**7.575 × 10^2^**	1.562 × 10^3^	1.521 × 10^3^	1.331 × 10^3^	1.876 × 10^3^
	Mean	1.105 × 10^3^	3.831 × 10^3^	1.982 × 10^3^	**8.252 × 10^2^**	1.783 × 10^3^	1.758 × 10^3^	2.042 × 10^3^	2.043 × 10^3^
	Std	6.703 × 10^1^	3.337 × 10^2^	1.064 × 10^2^	8.547 × 10^1^	1.192 × 10^2^	1.149 × 10^2^	4.587 × 10^2^	6.904 × 10^1^
	P	~	1.734 × 10^−6^	1.734 × 10^−6^	1.921 × 10^−6^	1.734 × 10^−6^	1.734 × 10^−6^	1.734 × 10^−6^	1.734 × 10^−6^
	Rank	2	15	12	1	8	7	11	14
F8	Best	9.602 × 10^2^	1.437 × 10^3^	1.274 × 10^3^	**8.289 × 10^2^**	1.183 × 10^3^	1.295 × 10^3^	1.190 × 10^3^	1.360 × 10^3^
	Mean	1.072 × 10^3^	1.526 × 10^3^	1.330 × 10^3^	**9.711 × 10^2^**	1.312 × 10^3^	1.395 × 10^3^	1.281 × 10^3^	1.471 × 10^3^
	Std	8.800 × 10^1^	5.481 × 10^1^	**2.441 × 10^1^**	2.310 × 10^2^	8.855 × 10^1^	3.750 × 10^1^	6.077 × 10^1^	5.009 × 10^1^
	P	~	1.734 × 10^−6^	1.734 × 10^−6^	4.716 × 10^−2^	2.353 × 10^−6^	1.734 × 10^−6^	2.603 × 10^−6^	1.734 × 10^−6^
	Rank	1	15	9	2	8	11	7	14
F9	Best	4.173 × 10^3^	3.058 × 10^4^	1.976 × 10^4^	**9.000 × 10^2^**	1.637 × 10^4^	2.269 × 10^4^	1.395 × 10^4^	2.355 × 10^4^
	Mean	1.330 × 10^4^	4.071 × 10^4^	2.890 × 10^4^	**3.669 × 10^3^**	3.075 × 10^4^	2.837 × 10^4^	2.026 × 10^4^	3.388 × 10^4^
	Std	5.241 × 10^3^	5.905 × 10^3^	3.713 × 10^3^	6.533 × 10^3^	9.405 × 10^3^	4.002 × 10^3^	5.560 × 10^3^	4.501 × 10^3^
	P	~	1.734 × 10^−6^	1.734 × 10^−6^	4.072 × 10^−5^	2.879 × 10^−6^	1.734 × 10^−6^	1.742 × 10^−4^	1.734 × 10^−6^
	Rank	2	15	12	1	10	11	3	13
F10	Best	**5.331 × 10^3^**	1.403 × 10^4^	1.051 × 10^4^	1.332 × 10^4^	9.941 × 10^3^	1.428 × 10^4^	6.823 × 10^3^	1.217 × 10^4^
	Mean	**7.455 × 10^3^**	1.513 × 10^4^	1.172 × 10^4^	1.442 × 10^4^	1.213 × 10^4^	1.503 × 10^4^	8.966 × 10^3^	1.427 × 10^4^
	Std	1.032 × 10^3^	4.793 × 10^2^	5.192 × 10^2^	5.358 × 10^2^	1.265 × 10^3^	**3.308 × 10^2^**	1.030 × 10^3^	8.864 × 10^2^
	P	~	1.734 × 10^−6^	1.734 × 10^−6^	1.734 × 10^−6^	1.734 × 10^−6^	1.734 × 10^−6^	3.882 × 10^−6^	1.734 × 10^−6^
	Rank	1	15	7	13	8	14	3	12
F11	Best	**1.315 × 10^3^**	1.143 × 10^4^	6.159 × 10^3^	3.858 × 10^4^	2.123 × 10^3^	5.233 × 10^3^	1.883 × 10^3^	1.095 × 10^4^
	Mean	1.510 × 10^3^	2.043 × 10^4^	1.526 × 10^4^	7.339 × 10^4^	3.171 × 10^3^	9.500 × 10^3^	1.736 × 10^4^	2.104 × 10^4^
	Std	9.670 × 10^1^	1.203 × 10^4^	3.671 × 10^3^	1.792 × 10^4^	9.160 × 10^2^	1.901 × 10^3^	1.249 × 10^4^	4.276 × 10^3^
	P	~	1.734 ×10^−6^	1.734 ×10^−6^	1.734 ×10^−6^	1.734 ×10^−6^	1.734 ×10^−6^	1.734 ×10^−6^	1.734 × 10^−6^
	Rank	2	12	11	15	5	8	10	13
F12	Best	9.151 × 10^6^	9.705 × 10^9^	1.302 × 10^9^	1.273 × 10^10^	1.814 × 10^8^	1.110 × 10^10^	4.279 × 10^7^	2.640 × 10^10^
	Mean	8.036 × 10^7^	1.966 × 10^10^	1.984 × 10^9^	2.112 × 10^10^	7.837 × 10^8^	1.634 × 10^10^	5.849 × 10^9^	6.262 × 10^10^
	Std	5.198 × 10^7^	7.818 × 10^9^	3.428 × 10^8^	3.878 × 10^9^	4.330 × 10^8^	3.352 × 10^9^	5.526 × 10^9^	1.873 × 10^10^
	P	~	1.734 × 10^−6^	1.734 × 10^−6^	1.734 × 10^−6^	1.734 × 10^−6^	1.734 × 10^−6^	1.921 × 10^−6^	1.734 × 10^−6^
	Rank	2	11	6	12	5	10	7	14
F13	Best	**6.911 × 10^4^**	2.926 × 10^9^	3.063 × 10^7^	5.651 × 10^9^	1.126 × 10^6^	1.483 × 10^9^	9.387 × 10^4^	5.348 × 10^9^
	Mean	**2.760 × 10^5^**	7.130 × 10^9^	7.706 × 10^7^	1.045 × 10^10^	3.440 × 10^7^	4.569 × 10^9^	1.407 × 10^9^	2.183 × 10^10^
	Std	1.413 × 10^5^	4.092 × 10^9^	2.636 × 10^7^	3.154 × 10^9^	3.330 × 10^7^	2.170 × 10^9^	1.782 × 10^9^	1.118 × 10^10^
	P	~	1.734 × 10^−6^	1.734 × 10^−6^	1.734 × 10^−6^	1.734 × 10^−6^	1.734 × 10^−6^	1.921 × 10^−6^	1.734 × 10^−6^
	Rank	1	11	6	13	5	10	7	15
F14	Best	**1.005 × 10^4^**	1.397 × 10^6^	1.661 × 10^6^	6.194 × 10^6^	2.529 × 10^5^	1.305 × 10^6^	1.010 × 10^5^	9.636 × 10^5^
	Mean	**1.527 × 10^5^**	6.551 × 10^6^	4.103 × 10^6^	2.208 × 10^7^	3.508 × 10^6^	4.671 × 10^6^	2.841 × 10^6^	4.175 × 10^7^
	Std	**9.436 × 10^4^**	5.301 × 10^6^	2.138 × 10^6^	1.355 × 10^7^	3.182 × 10^6^	2.397 × 10^6^	3.710 × 10^6^	5.339 × 10^7^
	P	~	1.734 × 10^−6^	1.734 × 10^−6^	1.734 × 10^−6^	1.734 × 10^−6^	1.734 × 10^−6^	4.286 × 10^−6^	1.734 × 10^−6^
	Rank	1	10	8	15	7	9	5	13
F15	Best	**1.761 × 10^4^**	6.001 × 10^8^	4.400 × 10^5^	9.952 × 10^8^	2.426 × 10^5^	3.054 × 10^8^	1.774 × 10^4^	1.864 × 10^8^
	Mean	8.831 × 10^4^	1.962 × 10^9^	8.798 × 10^6^	2.159 × 10^9^	4.090 × 10^6^	7.343 × 10^8^	3.749 × 10^7^	4.677 × 10^9^
	Std	6.555 × 10^4^	9.254 × 10^8^	6.060 × 10^6^	9.090 × 10^8^	5.374 × 10^6^	3.083 × 10^8^	1.064 × 10^8^	3.729 × 10^9^
	P	~	1.734 × 10^−6^	1.734 × 10^−6^	1.734 × 10^−6^	1.734 × 10^−6^	1.734 × 10^−6^	1.319 × 10^−2^	1.734 × 10^−6^
	Rank	2	12	7	13	6	10	3	14
F16	Best	**2.452 × 10^3^**	5.252 × 10^3^	4.100 × 10^3^	5.751 × 10^3^	4.368 × 10^3^	5.315 × 10^3^	3.132 × 10^3^	5.892 ×10^3^
	Mean	**3.306 × 10^3^**	6.242 × 10^3^	4.553 × 10^3^	6.758 × 10^3^	6.046 × 10^3^	6.015 × 10^3^	4.224 × 10^3^	7.739 ×10^3^
	Std	4.677 × 10^2^	5.064 × 10^2^	**1.969 × 10^2^**	5.169 × 10^2^	9.941 × 10^2^	3.963 × 10^2^	6.167 × 10^2^	1.485 ×10^3^
	P	~	1.734 × 10^−6^	1.734 × 10^−6^	1.734 × 10^−6^	1.734 × 10^−6^	1.734 × 10^−6^	5.752 × 10^−6^	1.734 ×10^−6^
	Rank	1	12	5	13	10	11	4	15
F17	Best	2.710 × 10^3^	5.270 × 10^3^	3.343 × 10^3^	**1.816 × 10^3^**	3.313 × 10^3^	4.075 × 10^3^	3.101 × 10^3^	4.465 × 10^3^
	Mean	3.132 × 10^3^	6.343 × 10^3^	3.868 × 10^3^	**2.293 × 10^3^**	4.222 × 10^3^	4.689 × 10^3^	4.014 × 10^3^	9.195 × 10^3^
	Std	3.703 × 10^2^	6.126 × 10^2^	**2.313 × 10^2^**	3.439 × 10^2^	5.352 × 10^2^	2.792 × 10^2^	4.062 × 10^2^	4.301 × 10^3^
	P	~	1.734 × 10^−6^	6.339 × 10^−6^	4.286 × 10^−6^	3.882 × 10^−6^	1.734 × 10^−6^	5.216 × 10^−6^	1.734 × 10^−6^
	Rank	2	13	6	1	8	12	7	14
F18	Best	3.191 × 10^5^	1.287 × 10^7^	6.511 × 10^6^	1.497 × 10^7^	3.445 × 10^6^	1.200 × 10^7^	**2.679 × 10** ^5^	1.141 × 10^7^
	Mean	**1.882 × 10^6^**	5.322 × 10^7^	1.558 × 10^7^	1.037 × 10^8^	3.186 × 10^7^	3.286 × 10^7^	1.747 × 10^7^	1.119 × 10^8^
	Std	1.564 × 10^6^	5.756 × 10^7^	6.622 × 10^6^	5.355 × 10^7^	2.591 × 10^7^	1.444 × 10^7^	4.818 × 10^7^	1.380 × 10^8^
	P	~	1.734 × 10^−6^	1.734 × 10^−6^	1.734 × 10^−6^	1.734 × 10^−6^	1.734 × 10^−6^	8.944 × 10^−4^	1.734 × 10^−6^
	Rank	1	12	7	15	9	11	3	13
F19	Best	8.193 × 10^4^	3.197 × 10^8^	1.772 × 10^5^	1.358 × 10^5^	7.185 × 10^4^	1.793 × 10^8^	**9.076 × 10^3^**	1.133 × 10^8^
	Mean	4.575 × 10^6^	9.218 × 10^8^	1.031 × 10^6^	1.107 × 10^9^	7.514 × 10^6^	5.133 × 10^8^	1.559 × 10^8^	2.286 × 10^9^
	Std	3.804 × 10^6^	4.387 × 10^8^	6.828 × 10^5^	6.306 × 10^8^	9.032 × 10^6^	2.382 × 10^8^	6.245 × 10^8^	1.531 × 10^9^
	P	~	1.734 × 10^−6^	4.072 × 10^−5^	1.734 × 10^−6^	1.109 × 10^−1^	1.734 × 10^−6^	6.733 × 10^−1^	1.734 × 10^−6^
	Rank	5	13	2	12	7	11	6	14
F20	Best	**2.308 × 10^3^**	3.714 × 10^3^	3.089 × 10^3^	2.734 × 10^3^	2.996 × 10^3^	3.821 × 10^3^	2.814 × 10^3^	3.296 × 10^3^
	Mean	**2.958 × 10^3^**	4.281 × 10^3^	3.511 × 10^3^	3.659 × 10^3^	3.836 × 10^3^	4.182 × 10^3^	3.608 × 10^3^	4.077 × 10^3^
	Std	3.188 × 10^2^	2.728 × 10^2^	1.914 × 10^2^	2.767 × 10^2^	3.166 × 10^2^	**1.754 × 10^2^**	4.235 × 10^2^	3.082 × 10^2^
	P	~	1.734 × 10^−6^	5.752 × 10^−6^	5.752 × 10^−6^	3.882 × 10^−6^	1.734 × 10^−6^	1.238 × 10^−5^	2.353 × 10^−6^
	Rank	1	15	5	9	10	14	8	12
F21	Best	2.462 × 10^3^	2.930 × 10^3^	2.780 × 10^3^	**2.336 × 10^3^**	2.707 × 10^3^	2.851 × 10^3^	2.606 × 10^3^	2.990 × 10^3^
	Mean	**2.529 × 10^3^**	3.035 × 10^3^	2.825 × 10^3^	2.609 × 10^3^	2.976 × 10^3^	2.926 × 10^3^	2.755 × 10^3^	3.155 × 10^3^
	Std	4.841 × 10^1^	5.365 × 10^1^	**2.249 × 10^1^**	2.773 × 10^2^	1.348 × 10^2^	3.807 × 10^1^	7.201 × 10^1^	7.925 × 10^1^
	P	~	1.734 × 10^−6^	1.734 × 10^−6^	5.710 × 10^−2^	1.734 × 10^−6^	1.734 × 10^−6^	1.734 × 10^−6^	1.734 × 10^−6^
	Rank	1	11	7	3	10	9	5	15
F22	Best	**2.321 × 10^3^**	1.476 × 10^4^	1.202 × 10^4^	1.486 × 10^4^	1.119 × 10^4^	1.542 × 10^4^	8.659 × 10^3^	1.447 × 10^4^
	Mean	**8.585 × 10^3^**	1.632 × 10^4^	1.346 × 10^4^	1.631 × 10^4^	1.334 × 10^4^	1.671 × 10^4^	1.062 × 10^4^	1.641 × 10^4^
	Std	2.286 × 10^3^	6.874 × 10^2^	**4.563 × 10^2^**	4.593 × 10^2^	1.148 × 10^3^	4.806 × 10^2^	1.036 × 10^3^	7.365 × 10^2^
	P	~	1.734 × 10^−6^	1.734 × 10^−6^	1.734 × 10^−6^	1.734 × 10^−6^	1.734 × 10^−6^	1.973 × 10^−5^	1.734 × 10^−6^
	Rank	1	13	8	11	7	15	2	14
F23	Best	**2.881 × 10^3^**	3.474 × 10^3^	3.121 × 10^3^	3.320 × 10^3^	3.374 × 10^3^	3.512 × 10^3^	3.074 × 10^3^	3.891 × 10^3^
	Mean	**2.999 × 10^3^**	3.778 × 10^3^	3.208 × 10^3^	3.440 × 10^3^	3.713 × 10^3^	3.643 × 10^3^	3.210 × 10^3^	4.382 × 10^3^
	Std	7.527 × 10^1^	1.561 × 10^2^	**2.480 × 10^1^**	4.137 × 10^1^	1.736 × 10^2^	6.663 × 10^1^	8.912 × 10^1^	2.724 × 10^2^
	P	~	1.734 ×10^−6^	1.734 ×10^−6^	1.734 ×10^−6^	1.734 ×10^−6^	1.734 ×10^−6^	1.734 ×10^−6^	1.734 × 10^−6^
	Rank	1	10	3	6	9	8	2	14
F24	Best	**3.050 × 10^3^**	3.537 × 10^3^	3.370 × 10^3^	3.387 × 10^3^	3.458 × 10^3^	3.632 × 10^3^	3.149 × 10^3^	4.125 × 10^3^
	Mean	**3.137 × 10^3^**	3.830 × 10^3^	3.421 × 10^3^	3.515 × 10^3^	3.744 × 10^3^	3.784 × 10^3^	3.237 × 10^3^	4.592 × 10^3^
	Std	6.548 × 10^1^	1.641 × 10^2^	**1.888 × 10** ^1^	4.167 × 10^1^	1.427 × 10^2^	5.931 × 10^1^	5.462 × 10^1^	3.345 × 10^2^
	P	~	1.734 × 10^−6^	1.734 × 10^−6^	1.734 × 10^−6^	1.734 × 10^−6^	1.734 × 10^−6^	8.188 × 10^−5^	1.734 × 10^−6^
	Rank	1	10	4	5	8	9	2	15
F25	Best	**3.035 × 10^3^**	9.853 × 10^3^	4.536 × 10^3^	3.144 × 10^3^	3.311 × 10^3^	5.470 × 10^3^	3.149 × 10^3^	1.067 × 10^4^
	Mean	**3.062 × 10^3^**	1.589 × 10^4^	5.142 × 10^3^	3.597 × 10^3^	3.568 × 10^3^	7.438 × 10^3^	5.326 × 10^3^	1.405 × 10^4^
	Std	2.471 × 10^1^	4.789 × 10^3^	3.462 × 10^2^	7.408 × 10^2^	1.741 × 10^2^	8.085 × 10^2^	1.944 × 10^3^	1.635 × 10^3^
	P	~	1.734 ×10^−6^	1.734 ×10^−6^	1.734 ×10^−6^	1.734 ×10^−6^	1.734 ×10^−6^	1.734 ×10^−6^	1.734 × 10^−6^
	Rank	1	14	8	4	5	11	7	13
F26	Best	**2.908 × 10^3^**	1.038 × 10^4^	7.969 × 10^3^	1.057 × 10^4^	1.098 × 10^4^	1.146 × 10^4^	7.699 × 10^3^	1.481 × 10^4^
	Mean	**3.980 × 10^3^**	1.303 × 10^4^	8.614 × 10^3^	1.129 × 10^4^	1.412 × 10^4^	1.300 × 10^4^	8.978 × 10^3^	1.707 × 10^4^
	Std	1.619 × 10^3^	1.380 × 10^3^	**2.064 × 10^2^**	4.330 × 10^2^	1.520 × 10^3^	1.014 × 10^3^	7.813 × 10^2^	8.721 × 10^2^
	P	~	1.734 ×10^−6^	1.734 ×10^−6^	1.734 ×10^−6^	1.734 ×10^−6^	1.734 ×10^−6^	1.734 ×10^−6^	1.734 × 10^−6^
	Rank	1	9	3	6	12	10	4	15
F27	Best	**3.250 × 10^3^**	3.493 × 10^3^	3.429 × 10^3^	3.688 × 10^3^	3.608 × 10^3^	4.335 × 10^3^	3.439 × 10^3^	5.561 × 10^3^
	Mean	**3.397 × 10^3^**	4.119 × 10^3^	3.496 × 10^3^	3.852 × 10^3^	4.428 × 10^3^	4.715 × 10^3^	3.630 × 10^3^	6.631 × 10^3^
	Std	1.427 × 10^2^	2.832 × 10^2^	**3.280 × 10^1^**	8.150 × 10^1^	4.151 × 10^2^	1.925 × 10^2^	1.457 × 10^2^	7.669 × 10^2^
	P	~	1.734 × 10^−6^	2.831 × 10^−4^	2.127 × 10^−6^	1.734 × 10^−6^	1.734 × 10^−6^	8.466 × 10^−6^	1.734 × 10^−6^
	Rank	1	7	2	5	9	11	3	15
F28	Best	**3.263 × 10^3^**	7.559 × 10^3^	7.933 × 10^3^	8.210 × 10^3^	3.590 × 10^3^	6.526 × 10^3^	3.953 × 10^3^	8.452 × 10^3^
	Mean	**3.298 × 10^3^**	9.642 × 10^3^	8.085 × 10^3^	9.587 × 10^3^	4.337 × 10^3^	7.755 × 10^3^	8.092 × 10^3^	1.159 × 10^4^
	Std	**2.288 × 10^1^**	9.258 × 10^2^	1.184 × 10^2^	4.430 × 10^2^	3.617 × 10^2^	8.141 × 10^2^	1.415 × 10^3^	1.227 × 10^3^
	P	~	1.734 × 10^−6^	1.734 × 10^−6^	1.734 × 10^−6^	1.734 × 10^−6^	1.734 × 10^−6^	1.734 × 10^−6^	1.734 × 10^−6^
	Rank	1	13	9	12	4	8	10	14
F29	Best	**3.893 × 10^3^**	6.629 × 10^3^	4.689 × 10^3^	9.349 × 10^3^	6.502 × 10^3^	6.533 × 10^3^	4.527 × 10^3^	1.067 × 10^4^
	Mean	**4.874 × 10^3^**	8.114 × 10^3^	5.023 × 10^3^	1.272 × 10^4^	8.284 × 10^3^	7.836 × 10^3^	5.350 × 10^3^	4.509 × 10^4^
	Std	5.387 × 10^2^	1.133 × 10^3^	**1.565 × 10^2^**	1.983 × 10^3^	1.363 × 10^3^	6.606 × 10^2^	4.681 × 10^2^	7.128 × 10^4^
	P	~	1.734 × 10^−6^	5.193 × 10^−2^	1.734 × 10^−6^	1.734 × 10^−6^	1.734 × 10^−6^	8.307 × 10^−4^	1.734 × 10^−6^
	Rank	1	9	2	13	10	8	3	15
F30	Best	6.073 × 10^7^	5.077 × 10^8^	4.159 × 10^6^	6.258 × 10^8^	1.190 × 10^8^	4.928 × 10^8^	**2.903 × 10^6^**	2.499 × 10^8^
	Mean	8.295 × 10^7^	1.491 × 10^9^	**1.316 × 10^7^**	2.243 × 10^9^	2.224 × 10^8^	8.574 × 10^8^	1.435 × 10^8^	3.582 × 10^9^
	Std	1.783 × 10^7^	6.420 × 10^8^	**5.784 × 10^6^**	8.871 × 10^8^	7.942 × 10^7^	3.370 × 10^8^	4.425 × 10^8^	2.391 × 10^9^
	P	~	1.734 × 10^−6^	1.734 × 10^−6^	1.734 × 10^−6^	1.734 × 10^−6^	1.734 × 10^−6^	7.731 × 10^−3^	1.734 × 10^−6^
	Rank	4	12	1	14	7	10	3	13
Mean Rank		**1.6**	12.3	6.5	8.5	7.8	10.2	5.7	13.8
+/=/−		~	29/0/0	27/2/0	24/0/5	28/1/0	29/0/0	28/1/0	29/0/0

**Table A13:** Comparison results with newly proposed algorithms on CEC2017 (Dim = 50).

Function	Index	MShOA	GJO	WAA	SHO	HO	PO	AO
F1	Best	7.835 × 10^10^	1.841 × 10^10^	3.650 × 10^6^	5.405 × 10^10^	2.176 × 10^10^	5.547 × 10^9^	1.622 × 10^8^
	Mean	1.082 × 10^11^	3.566 × 10^10^	7.080 × 10^6^	7.060 × 10^10^	3.270 × 10^10^	1.337 × 10^10^	4.351 × 10^8^
	Std	1.225 × 10^10^	8.493 × 10^9^	**1.399 × 10^6^**	6.980 × 10^9^	4.488 × 10^9^	5.804 × 10^9^	1.686 × 10^8^
	P	1.734 × 10^−6^	1.734 × 10^−6^	1.891 × 10^−4^	1.734 × 10^−6^	1.734 × 10^−6^	1.734 × 10^−6^	1.734 × 10^−6^
	Rank	15	10	1	12	9	6	4
F3	Best	1.405 × 10^5^	8.014 × 10^4^	8.869 × 10^4^	1.176 × 10^5^	1.403 × 10^5^	5.281 × 10^4^	1.310 × 10^5^
	Mean	1.927 × 10^5^	1.221 × 10^5^	1.178 × 10^5^	1.465 × 10^5^	1.589 × 10^5^	8.382 × 10^4^	1.765 × 10^5^
	Std	2.322 × 10^4^	1.710 × 10^4^	1.300 × 10^4^	1.205 × 10^4^	1.115 × 10^4^	1.464 × 10^4^	2.168 × 10^4^
	P	1.734 × 10^−6^	1.734 × 10^−6^	1.734 × 10^−6^	1.734 × 10^−6^	1.734 × 10^−6^	1.734 × 10^−6^	1.734 × 10^−6^
	Rank	9	4	3	5	7	2	8
F4	Best	1.661 × 10^4^	2.434 × 10^3^	5.350 × 10^2^	1.101 × 10^4^	2.196 × 10^3^	1.031 × 10^3^	7.020 × 10^2^
	Mean	3.530 × 10^4^	5.130 × 10^3^	**5.567 × 10^2^**	1.504 × 10^4^	5.386 × 10^3^	1.851 × 10^3^	8.428 × 10^2^
	Std	9.532 × 10^3^	1.420 × 10^3^	**2.956 × 10^1^**	2.791 × 10^3^	1.542 × 10^3^	6.513 × 10^2^	8.184 × 10^1^
	P	1.734 × 10^−6^	1.734 × 10^−6^	4.897 × 10^−4^	1.734 × 10^−6^	1.734 × 10^−6^	1.734 × 10^−6^	1.734 × 10^−6^
	Rank	15	8	1	13	9	5	3
F5	Best	1.056 × 10^3^	7.696 × 10^2^	8.713 × 10^2^	9.996 × 10^2^	9.530 × 10^2^	9.020 × 10^2^	7.782 × 10^2^
	Mean	1.161 × 10^3^	9.010 × 10^2^	9.027 × 10^2^	1.060 × 10^3^	1.024 × 10^3^	9.601 × 10^2^	8.623 × 10^2^
	Std	6.542 × 10^1^	5.738 × 10^1^	**2.284 × 10^1^**	3.149 × 10^1^	3.454 × 10^1^	3.368 × 10^1^	3.539 × 10^1^
	P	1.734 × 10^−6^	2.879 × 10^−6^	1.734 × 10^−6^	1.734 × 10^−6^	1.734 × 10^−6^	1.734 × 10^−6^	4.072 × 10^−5^
	Rank	13	4	5	11	9	6	3
F6	Best	6.682 × 10^2^	6.342 × 10^2^	6.689 × 10^2^	6.791 × 10^2^	6.754 × 10^2^	6.633 × 10^2^	6.431 × 10^2^
	Mean	6.986 × 10^2^	6.545 × 10^2^	6.719 × 10^2^	6.864 × 10^2^	6.870 × 10^2^	6.753 × 10^2^	6.625 × 10^2^
	Std	1.322 × 10^1^	1.077 × 10^1^	**1.480 × 10^0^**	4.322 × 10^0^	5.163 × 10^0^	7.720 × 10^0^	7.053 × 10^0^
	P	1.734 × 10^−6^	1.709 × 10^−3^	2.353 × 10^−6^	1.921 × 10^−6^	1.734 × 10^−6^	2.353 × 10^−6^	3.405 × 10^−5^
	Rank	14	3	7	10	12	8	6
F7	Best	1.754 × 10^3^	1.259 × 10^3^	1.747 × 10^3^	1.582 × 10^3^	1.615 × 10^3^	1.489 × 10^3^	1.336 × 10^3^
	Mean	2.035 × 10^3^	1.417 × 10^3^	1.826 × 10^3^	1.755 × 10^3^	1.840 × 10^3^	1.729 × 10^3^	1.522 × 10^3^
	Std	9.753 × 10^1^	1.010 × 10^2^	**3.961 × 10^1^**	7.445 × 10^1^	9.005 × 10^1^	1.187 × 10^2^	1.084 × 10^2^
	P	1.734 × 10^−6^	1.734 × 10^−6^	1.734 × 10^−6^	1.734 × 10^−6^	1.734 × 10^−6^	1.734 × 10^−6^	1.734 × 10^−6^
	Rank	13	3	9	5	10	6	4
F8	Best	1.362 × 10^3^	1.111 × 10^3^	1.164 × 10^3^	1.310 × 10^3^	1.200 × 10^3^	1.175 × 10^3^	1.119 × 10^3^
	Mean	1.470 × 10^3^	1.252 × 10^3^	1.217 × 10^3^	1.391 × 10^3^	1.344 × 10^3^	1.270 × 10^3^	1.192 × 10^3^
	Std	6.611 × 10^1^	8.667 × 10^1^	2.989 × 10^1^	3.646 × 10^1^	4.925 × 10^1^	4.622 × 10^1^	3.944 × 10^1^
	P	1.734 × 10^−6^	3.515 × 10^−6^	5.216 × 10^−6^	1.734 × 10^−6^	1.921 × 10^−6^	4.729 × 10^−6^	1.494 × 10^−5^
	Rank	13	5	4	12	10	6	3
F9	Best	2.789 × 10^4^	7.173 × 10^3^	1.694 × 10^4^	1.858 × 10^4^	1.896 × 10^4^	1.416 × 10^4^	1.253 × 10^4^
	Mean	3.680 × 10^4^	2.056 × 10^4^	2.107 × 10^4^	2.583 × 10^4^	2.534 × 10^4^	2.113 × 10^4^	2.043 × 10^4^
	Std	4.938 × 10^3^	6.323 × 10^3^	3.001 × 10^3^	3.013 × 10^3^	**2.934 × 10^3^**	3.738 × 10^3^	3.492 × 10^3^
	P	1.734 × 10^−6^	2.225 × 10^−4^	3.882 × 10^−6^	1.734 × 10^−6^	2.127 × 10^−6^	8.466 × 10^−6^	3.112 × 10^−5^
	Rank	14	6	4	9	8	7	5
F10	Best	1.302 × 10^4^	7.835 × 10^3^	7.114 × 10^3^	1.067 × 10^4^	1.017 × 10^4^	7.489 × 10^3^	6.922 × 10^3^
	Mean	1.403 × 10^4^	1.011 × 10^4^	9.040 × 10^3^	1.250 × 10^4^	1.285 × 10^4^	1.038 × 10^4^	9.467 × 10^3^
	Std	6.440 × 10^2^	1.676 × 10^3^	9.493 × 10^2^	8.322 × 10^2^	1.077 × 10^3^	1.095 × 10^3^	1.054 × 10^3^
	P	1.734 × 10^−6^	3.515 × 10^−6^	1.799 × 10^−5^	1.734 × 10^−6^	1.734 × 10^−6^	1.734 × 10^−6^	1.025 × 10^−5^
	Rank	11	5	2	9	10	6	4
F11	Best	1.423 × 10^4^	3.768 × 10^3^	1.318 × 10^3^	8.182 × 10^3^	4.152 × 10^3^	1.674 × 10^3^	1.612 × 10^3^
	Mean	2.558 × 10^4^	9.406 × 10^3^	**1.383 × 10^3^**	1.335 × 10^4^	8.748 × 10^3^	2.599 × 10^3^	1.928 × 10^3^
	Std	5.972 × 10^3^	2.477 × 10^3^	**2.548 × 10^1^**	2.511 × 10^3^	1.876 × 10^3^	6.849 × 10^2^	1.696 × 10^2^
	P	1.734 × 10^−6^	1.734 × 10^−6^	8.466 × 10^−6^	1.734 × 10^−6^	1.734 × 10^−6^	1.734 × 10^−6^	1.734 × 10^−6^
	Rank	14	7	1	9	6	4	3
F12	Best	1.845 × 10^10^	2.574 × 10^9^	**3.342 × 10^6^**	1.809 × 10^10^	2.755 × 10^9^	7.718 × 10^7^	9.093 × 10^7^
	Mean	6.629 × 10^10^	8.617 × 10^9^	**1.669 × 10^7^**	3.178 × 10^10^	1.045 × 10^10^	7.909 × 10^8^	3.956 × 10^8^
	Std	2.838 × 10^10^	4.502 × 10^9^	**1.075 × 10^7^**	8.147 × 10^9^	4.295 × 10^9^	8.820 × 10^8^	2.122 × 10^8^
	P	1.734 × 10^−6^	1.734 × 10^−6^	6.339 × 10^−6^	1.734 × 10^−6^	1.734 × 10^−6^	2.127 × 10^−6^	2.127 × 10^−6^
	Rank	15	8	1	13	9	4	3
F13	Best	2.155 × 10^9^	6.674 × 10^7^	2.174 × 10^5^	2.954 × 10^8^	6.766 × 10^8^	2.654 × 10^5^	1.753 × 10^6^
	Mean	2.416 × 10^10^	1.433 × 10^9^	4.079 × 10^5^	9.080 × 10^9^	2.915 × 10^9^	2.732 × 10^7^	5.543 × 10^6^
	Std	1.948 × 10^10^	1.422 × 10^9^	**1.137 × 10^5^**	7.337 × 10^9^	2.731 × 10^9^	3.206 × 10^7^	3.895 × 10^6^
	P	1.734 × 10^−6^	1.734 × 10^−6^	1.833 × 10^−3^	1.734 × 10^−6^	1.734 × 10^−6^	1.734 × 10^−6^	1.734 × 10^−6^
	Rank	14	8	2	12	9	4	3
F14	Best	3.554 × 10^6^	2.989 × 10^5^	5.004 × 10^4^	1.065 × 10^6^	7.963 × 10^5^	1.486 × 10^5^	3.999 × 10^5^
	Mean	5.651 × 10^7^	2.200 × 10^6^	2.131 × 10^5^	8.185 × 10^6^	7.552 × 10^6^	1.372 × 10^6^	2.291 × 10^6^
	Std	8.221 × 10^7^	2.958 × 10^6^	1.380 × 10^5^	1.058 × 10^7^	5.575 × 10^6^	1.221 × 10^6^	1.679 × 10^6^
	P	1.734 × 10^−6^	1.921 × 10^−6^	1.650 × 10^−1^	1.734 × 10^−6^	1.734 × 10^−6^	1.921 × 10^−6^	1.734 × 10^−6^
	Rank	14	4	2	11	12	3	6
F15	Best	1.284 × 10^8^	2.428 × 10^5^	2.999 × 10^4^	8.296 × 10^7^	1.508 × 10^7^	2.550 × 10^4^	1.810 × 10^5^
	Mean	6.400 × 10^9^	6.625 × 10^8^	**6.205 × 10^4^**	1.118 × 10^9^	1.497 × 10^8^	2.513 × 10^6^	5.533 × 10^5^
	Std	3.540 × 10^9^	8.635 × 10^8^	**2.873 × 10^4^**	6.482 × 10^8^	1.454 × 10^8^	5.422 × 10^6^	2.441 × 10^5^
	P	1.734 × 10^−6^	1.921 × 10^−6^	4.716 × 10^−2^	1.734 × 10^−6^	1.734 × 10^−6^	2.412 × 10^−4^	1.734 × 10^−6^
	Rank	15	9	1	11	8	4	5
F16	Best	5.207 × 10^3^	2.729 × 10^3^	3.481 × 10^3^	4.003 × 10^3^	3.675 × 10^3^	3.581 × 10^3^	3.225 × 10^3^
	Mean	7.048 × 10^3^	3.980 × 10^3^	4.537 × 10^3^	5.384 × 10^3^	5.319 × 10^3^	4.917 × 10^3^	4.052 × 10^3^
	Std	1.132 × 10^3^	6.531 × 10^2^	5.821 × 10^2^	7.051 × 10^2^	7.213 × 10^2^	6.682 × 10^2^	4.685 × 10^2^
	P	1.734 × 10^−6^	1.287 × 10^−3^	3.882 × 10^−6^	1.734 × 10^−6^	1.734 × 10^−6^	1.921 × 10^−6^	1.734 × 10^−6^
	Rank	14	2	6	9	8	7	3
F17	Best	5.192 × 10^3^	2.624 × 10^3^	3.253 × 10^3^	3.289 × 10^3^	3.430 × 10^3^	3.557 × 10^3^	2.954 × 10^3^
	Mean	3.746 × 10^4^	3.506 × 10^3^	3.743 × 10^3^	4.330 × 10^3^	4.318 × 10^3^	4.376 × 10^3^	3.654 × 10^3^
	Std	4.286 × 10^4^	4.253 × 10^2^	3.239 × 10^2^	5.489 × 10^2^	4.941 × 10^2^	5.467 × 10^2^	3.785 × 10^2^
	P	1.734 × 10^−6^	6.156 × 10^−4^	1.799 × 10^−5^	1.921 × 10^−6^	1.921 × 10^−6^	2.127 × 10^−6^	5.752 × 10^−6^
	Rank	15	3	5	9	11	10	4
F18	Best	8.981 × 10^6^	1.711 × 10^6^	9.910 × 10^5^	1.498 × 10^6^	6.988 × 10^6^	5.206 × 10^5^	1.701 × 10^6^
	Mean	2.095 × 10^8^	1.313 × 10^7^	1.981 × 10^6^	2.207 × 10^7^	3.473 × 10^7^	7.042 × 10^6^	8.701 × 10^6^
	Std	3.033 × 10^8^	2.017 × 10^7^	**1.241 × 10^6^**	1.896 × 10^7^	2.194 × 10^7^	4.800 × 10^6^	6.392 × 10^6^
	P	1.734 × 10^−6^	1.973 × 10^−5^	5.170 × 10^−1^	1.921 × 10^−6^	1.734 × 10^−6^	3.112 × 10^−5^	6.984 × 10^−6^
	Rank	14	6	2	8	10	4	5
F19	Best	1.679 × 10^8^	1.486 × 10^5^	3.754 × 10^4^	2.178 × 10^6^	1.402 × 10^7^	5.512 × 10^5^	1.717 × 10^5^
	Mean	3.744 × 10^9^	1.707 × 10^8^	**3.337 × 10^5^**	5.231 × 10^8^	9.778 × 10^7^	3.974 × 10^6^	2.925 × 10^6^
	Std	3.330 × 10^9^	3.339 × 10^8^	**4.277 × 10^5^**	7.492 × 10^8^	1.016 × 10^8^	3.476 × 10^6^	2.795 × 10^6^
	P	1.734 × 10^−6^	3.724 × 10^−5^	6.339 × 10^−6^	1.921 × 10^−6^	1.734 × 10^−6^	4.653 × 10^−1^	7.865 × 10^−2^
	Rank	15	8	1	10	9	4	3
F20	Best	2.983 × 10^3^	2.656 × 10^3^	3.077 × 10^3^	2.891 × 10^3^	2.971 × 10^3^	2.901 × 10^3^	2.854 × 10^3^
	Mean	4.258 × 10^3^	3.313 × 10^3^	3.823 × 10^3^	3.547 × 10^3^	3.503 × 10^3^	3.573 × 10^3^	3.334 × 10^3^
	Std	6.069 × 10^2^	3.769 × 10^2^	3.028 × 10^2^	2.893 × 10^2^	2.355 × 10^2^	3.042 × 10^2^	2.758 × 10^2^
	P	2.353 × 10^−6^	4.534 × 10^−4^	1.921 × 10^−6^	6.339 × 10^−6^	3.182 × 10^−6^	6.984 × 10^−6^	4.897 × 10^−4^
	Rank	13	3	11	6	4	7	2
F21	Best	2.929 × 10^3^	2.630 × 10^3^	2.933 × 10^3^	2.854 × 10^3^	2.862 × 10^3^	2.624 × 10^3^	2.508 × 10^3^
	Mean	3.072 × 10^3^	2.716 × 10^3^	3.163 × 10^3^	2.928 × 10^3^	3.046 × 10^3^	2.776 × 10^3^	2.650 × 10^3^
	Std	7.445 × 10^1^	8.263 × 10^1^	1.076 × 10^2^	4.520 × 10^1^	6.708 × 10^1^	8.105 × 10^1^	5.887 × 10^1^
	P	1.734 × 10^−6^	2.127 × 10^−6^	1.734 × 10^−6^	1.734 × 10^−6^	1.734 × 10^−6^	1.921 × 10^−6^	2.353 × 10^−6^
	Rank	13	4	14	8	12	6	2
F22	Best	1.453 × 10^4^	9.189 × 10^3^	9.903 × 10^3^	1.385 × 10^4^	1.302 × 10^4^	9.206 × 10^3^	2.874 × 10^3^
	Mean	1.632 × 10^4^	1.256 × 10^4^	1.081 × 10^4^	1.531 × 10^4^	1.453 × 10^4^	1.252 × 10^4^	1.045 × 10^4^
	Std	7.771 × 10^2^	2.175 × 10^3^	5.748 × 10^2^	6.698 × 10^2^	8.855 × 10^2^	1.154 × 10^3^	2.025 × 10^3^
	P	1.734 × 10^−6^	2.353 × 10^−6^	3.882 × 10^−6^	1.734 × 10^−6^	1.734 × 10^−6^	2.353 × 10^−6^	1.477 × 10^−4^
	Rank	12	6	3	10	9	5	4
F23	Best	3.597 × 10^3^	3.115 × 10^3^	3.895 × 10^3^	3.615 × 10^3^	3.509 × 10^3^	3.270 × 10^3^	3.079 × 10^3^
	Mean	3.944 × 10^3^	3.308 × 10^3^	4.480 × 10^3^	3.936 × 10^3^	3.820 × 10^3^	3.494 × 10^3^	3.264 × 10^3^
	Std	1.790 × 10^2^	1.226 × 10^2^	2.421 × 10^2^	1.742 × 10^2^	1.836 × 10^2^	1.217 × 10^2^	8.388 × 10^1^
	P	1.734 × 10^−6^	2.127 × 10^−6^	1.734 × 10^−6^	1.734 × 10^−6^	1.734 × 10^−6^	1.734 × 10^−6^	1.734 × 10^−6^
	Rank	13	5	15	12	11	7	4
F24	Best	3.872 × 10^3^	3.330 × 10^3^	3.375 × 10^3^	3.967 × 10^3^	3.716 × 10^3^	3.356 × 10^3^	3.175 × 10^3^
	Mean	4.237 × 10^3^	3.532 × 10^3^	4.415 × 10^3^	4.268 × 10^3^	4.071 × 10^3^	3.588 × 10^3^	3.331 × 10^3^
	Std	2.174 × 10^2^	1.123 × 10^2^	4.457 × 10^2^	1.337 × 10^2^	2.001 × 10^2^	1.228 × 10^2^	7.759 × 10^1^
	P	1.734 × 10^−6^	1.734 × 10^−6^	1.734 × 10^−6^	1.734 × 10^−6^	1.734 × 10^−6^	1.734 × 10^−6^	2.127 × 10^−6^
	Rank	12	6	14	13	11	7	3
F25	Best	1.055 × 10^4^	4.648 × 10^3^	3.064 × 10^3^	6.957 × 10^3^	4.370 × 10^3^	3.390 × 10^3^	3.147 × 10^3^
	Mean	1.567 × 10^4^	5.929 × 10^3^	3.100 × 10^3^	8.682 × 10^3^	5.557 × 10^3^	3.974 × 10^3^	3.264 × 10^3^
	Std	2.473 × 10^3^	6.274 × 10^2^	**1.350 × 10^1^**	1.188 × 10^3^	5.549 × 10^2^	3.779 × 10^2^	7.236 × 10^1^
	P	1.734 × 10^−6^	1.734 × 10^−6^	2.597 × 10^−5^	1.734 × 10^−6^	1.734 × 10^−6^	1.734 × 10^−6^	1.734 × 10^−6^
	Rank	15	10	2	12	9	6	3
F26	Best	1.259 × 10^4^	8.273 × 10^3^	1.126 × 10^4^	1.160 × 10^4^	1.221 × 10^4^	9.227 × 10^3^	4.110 × 10^3^
	Mean	1.726 × 10^4^	9.522 × 10^3^	1.233 × 10^4^	1.389 × 10^4^	1.368 × 10^4^	1.202 × 10^4^	6.271 × 10^3^
	Std	1.651 × 10^3^	8.054 × 10^2^	4.710 × 10^2^	9.440 × 10^2^	8.185 × 10^2^	1.268 × 10^3^	2.557 × 10^3^
	P	1.734 × 10^−6^	1.734 × 10^−6^	1.734 × 10^−6^	1.734 × 10^−6^	1.734 × 10^−6^	1.734 × 10^−6^	1.359 × 10^−4^
	Rank	14	5	8	13	11	7	2
F27	Best	4.663 × 10^3^	3.770 × 10^3^	4.168 × 10^3^	4.947 × 10^3^	4.146 × 10^3^	3.599 × 10^3^	3.563 × 10^3^
	Mean	5.952 × 10^3^	4.110 × 10^3^	5.986 × 10^3^	5.540 × 10^3^	4.733 × 10^3^	4.075 × 10^3^	3.762 × 10^3^
	Std	7.299 × 10^2^	2.243 × 10^2^	8.394 × 10^2^	3.015 × 10^2^	3.659 × 10^2^	2.871 × 10^2^	1.161 × 10^2^
	P	1.734 × 10^−6^	1.734 × 10^−6^	1.734 × 10^−6^	1.734 × 10^−6^	1.734 × 10^−6^	1.734 × 10^−6^	2.603 × 10^−6^
	Rank	13	8	14	12	10	6	4
F28	Best	9.020 × 10^3^	4.968 × 10^3^	3.281 × 10^3^	7.122 × 10^3^	5.389 × 10^3^	3.878 × 10^3^	3.629 × 10^3^
	Mean	1.224 × 10^4^	6.152 × 10^3^	**3.319 × 10^3^**	8.347 × 10^3^	6.533 × 10^3^	4.593 × 10^3^	3.885 × 10^3^
	Std	1.823 × 10^3^	5.811 × 10^2^	3.000 × 10^1^	6.567 × 10^2^	7.110 × 10^2^	3.612 × 10^2^	1.831 × 10^2^
	P	1.734 × 10^−6^	1.734 × 10^−6^	8.730 × 10^−3^	1.734 × 10^−6^	1.734 × 10^−6^	1.734 × 10^−6^	1.734 × 10^−6^
	Rank	15	6	2	11	7	5	3
F29	Best	6.125 × 10^3^	4.921 × 10^3^	5.262 × 10^3^	7.129 × 10^3^	6.153 × 10^3^	5.714 × 10^3^	4.864 × 10^3^
	Mean	4.269 × 10^4^	5.875 × 10^3^	6.256 × 10^3^	9.986 × 10^3^	9.121 × 10^3^	7.476 × 10^3^	5.807 × 10^3^
	Std	7.984 × 10^4^	6.059 × 10^2^	7.167 × 10^2^	2.325 × 10^3^	1.312 × 10^3^	9.536 × 10^2^	5.735 × 10^2^
	P	1.734 × 10^−6^	2.163 × 10^−5^	1.921 × 10^−6^	1.734 × 10^−6^	1.734 × 10^−6^	1.734 × 10^−6^	6.984 × 10^−6^
	Rank	14	5	6	12	11	7	4
F30	Best	3.999 × 10^8^	1.297 × 10^8^	1.984 × 10^7^	3.334 × 10^8^	2.383 × 10^8^	4.805 × 10^7^	3.267 × 10^7^
	Mean	5.782 × 10^9^	4.056 × 10^8^	2.802 × 10^7^	1.449 × 10^9^	6.401 × 10^8^	1.680 × 10^8^	9.314 × 10^7^
	Std	3.446 × 10^9^	2.824 × 10^8^	7.385 × 10^6^	1.777 × 10^9^	2.925 × 10^8^	6.089 × 10^7^	3.034 × 10^7^
	P	1.734 × 10^−6^	1.734 × 10^−6^	1.734 × 10^−6^	1.734 × 10^−6^	1.734 × 10^−6^	4.729 × 10^−6^	1.306 × 10^−1^
	Rank	15	8	2	11	9	6	5
Mean Rank		13.7	5.8	5.1	10.3	9.3	5.7	3.8
+/=/−		29/0/0	29/0/0	19/2/8	29/0/0	29/0/0	28/1/0	27/2/0

**Table A14:** Comparison results with classic highly-cited algorithms on CEC2017 (Dim = 100).

Function	Index	EAO	PSO	DE	CMA-ES	WOA	SCA	MFO	HHO
F1	Best	2.173 × 10^7^	2.645 × 10^11^	1.134 × 10^11^	1.383 × 10^2^	4.931 × 10^9^	1.606 × 10^11^	6.216 × 10^10^	2.083 × 10^11^
	Mean	2.242 × 10^8^	3.458 × 10^11^	1.244 × 10^11^	3.255 × 10^9^	8.462 × 10^9^	1.822 × 10^11^	1.391 × 10^11^	2.508 × 10^11^
	Std	1.162 × 10^8^	5.222 × 10^10^	5.367 × 10^9^	1.783 × 10^10^	1.908 × 10^9^	1.095 × 10^10^	4.827 × 10^10^	1.625 × 10^10^
	P	~	1.734 × 10^−6^	1.734 × 10^−6^	3.112 × 10^−5^	1.734 × 10^−6^	1.734 × 10^−6^	1.734 × 10^−6^	1.734 × 10^−6^
	Rank	3	15	8	1	5	11	10	13
F3	Best	**1.139 × 10^5^**	7.050 × 10^5^	8.292 × 10^5^	6.773 × 10^5^	4.215 × 10^5^	3.228 × 10^5^	5.245 × 10^5^	3.072 × 10^5^
	Mean	**1.474 × 10^5^**	1.041 × 10^6^	9.483 × 10^5^	8.325 × 10^5^	8.769 × 10^5^	3.858 × 10^5^	8.345 × 10^5^	3.592 × 10^5^
	Std	2.328 × 10^4^	1.872 × 10^5^	6.877 × 10^4^	7.870 × 10^4^	1.699 × 10^5^	3.792 × 10^4^	1.753 × 10^5^	1.243 × 10^4^
	P	~	1.734 × 10^−6^	1.734 × 10^−6^	1.734 × 10^−6^	1.734 × 10^−6^	1.734 × 10^−6^	1.734 × 10^−6^	1.734 × 10^−6^
	Rank	1	15	14	11	13	10	12	9
F4	Best	6.605 × 10^2^	4.249 × 10^4^	1.253 × 10^4^	1.030 × 10^4^	2.003 × 10^3^	2.418 × 10^4^	4.862 × 10^3^	5.476 × 10^4^
	Mean	7.546 × 10^2^	6.868 × 10^4^	1.607 × 10^4^	1.894 × 10^4^	3.115 × 10^3^	3.609 × 10^4^	2.686 × 10^4^	8.727 × 10^4^
	Std	5.421 × 10^1^	2.116 × 10^4^	1.851 × 10^3^	3.657 × 10^3^	6.220 × 10^2^	6.547 × 10^3^	1.510 × 10^4^	1.327 × 10^4^
	P	~	1.734 × 10^−6^	1.734 × 10^−6^	1.734 × 10^−6^	1.734 × 10^−6^	1.734 × 10^−6^	1.734 × 10^−6^	1.734 × 10^−6^
	Rank	2	13	7	9	4	11	10	14
F5	Best	9.560 × 10^2^	2.072 × 10^3^	1.901 × 10^3^	**6.044 × 10^2^**	1.402 × 10^3^	1.832 × 10^3^	1.434 × 10^3^	1.876 × 10^3^
	Mean	1.224 × 10^3^	2.292 × 10^3^	1.992 × 10^3^	**9.230 × 10^2^**	1.672 × 10^3^	1.953 × 10^3^	1.813 × 10^3^	2.067 × 10^3^
	Std	1.415 × 10^2^	1.228 × 10^2^	**3.611 × 10^1^**	4.905 × 10^2^	1.442 × 10^2^	6.157 × 10^1^	1.555 × 10^2^	6.572 × 10^1^
	P	~	1.734 × 10^−6^	1.734 × 10^−6^	1.734 × 10^−6^	1.734 × 10^−6^	1.734 × 10^−6^	1.734 × 10^−6^	1.734 × 10^−6^
	Rank	1	15	12	2	7	11	9	14
F6	Best	6.429 × 10^2^	7.104 × 10^2^	6.579 × 10^2^	**6.000 × 10^2^**	6.824 × 10^2^	6.879 × 10^2^	6.545 × 10^2^	7.036 × 10^2^
	Mean	6.606 × 10^2^	7.263 × 10^2^	6.651 × 10^2^	**6.000 × 10^2^**	6.964 × 10^2^	6.970 × 10^2^	6.776 × 10^2^	7.111 × 10^2^
	Std	7.103 × 10^0^	7.743 × 10^0^	2.523 × 10^0^	**1.473 × 10^−3^**	1.132 × 10^1^	5.029 × 10^0^	9.601 × 10^0^	4.727 × 10^0^
	P	~	1.734 × 10^−6^	2.958 × 10^−3^	1.734 × 10^−6^	1.734 × 10^−6^	1.734 × 10^−6^	5.217 × 10^−6^	1.734 × 10^−6^
	Rank	2	15	3	1	9	10	7	14
F7	Best	1.483 × 10^3^	8.201 × 10^3^	5.902 × 10^3^	**8.200 × 10^2^**	3.171 × 10^3^	3.310 × 10^3^	3.889 × 10^3^	3.725 × 10^3^
	Mean	1.792 × 10^3^	9.082 × 10^3^	6.526 × 10^3^	**9.338 × 10^2^**	3.486 × 10^3^	3.658 × 10^3^	5.285 × 10^3^	3.984 × 10^3^
	Std	2.102 × 10^2^	5.480 × 10^2^	2.879 × 10^2^	**3.246 × 10^1^**	1.731 × 10^2^	1.818 × 10^2^	6.602 × 10^2^	9.074 × 10^1^
	P	~	1.734 × 10^−6^	1.734 × 10^−6^	1.734 × 10^−6^	1.734 × 10^−6^	1.734 × 10^−6^	1.734 × 10^−6^	1.734 × 10^−6^
	Rank	2	15	14	1	7	9	13	12
F8	Best	1.258 × 10^3^	2.405 × 10^3^	2.179 × 10^3^	**8.965 × 10^2^**	1.915 × 10^3^	2.197 × 10^3^	1.860 × 10^3^	2.355 × 10^3^
	Mean	**1.455 × 10^3^**	2.623 × 10^3^	2.266 × 10^3^	1.529 × 10^3^	2.139 × 10^3^	2.321 × 10^3^	2.185 × 10^3^	2.536 × 10^3^
	Std	1.066 × 10^2^	1.019 × 10^2^	4.654 × 10^1^	5.724 × 10^2^	1.620 × 10^2^	5.439 × 10^1^	1.678 × 10^2^	8.131 × 10^1^
	P	~	1.734 × 10^−6^	1.734 × 10^−6^	2.369 × 10^−1^	1.734 × 10^−6^	1.734 × 10^−6^	1.734 × 10^−6^	1.734 × 10^−6^
	Rank	1	15	10	4	7	11	8	14
F9	Best	2.437 × 10^4^	1.057 × 10^5^	1.064 × 10^5^	**9.000 × 10^2^**	3.640 × 10^4^	6.514 × 10^4^	3.793 × 10^4^	5.831 × 10^4^
	Mean	3.602 × 10^4^	1.334 × 10^5^	1.189 × 10^5^	**6.286 × 10^3^**	5.482 × 10^4^	7.717 × 10^4^	4.904 × 10^4^	7.477 × 10^4^
	Std	6.490 × 10^3^	2.073 × 10^4^	5.907 × 10^3^	1.410 × 10^4^	1.275 × 10^4^	6.451 × 10^3^	5.479 × 10^3^	7.688 × 10^3^
	P	~	1.734 × 10^−6^	1.734 × 10^−6^	4.729 × 10^−6^	1.734 × 10^−6^	1.734 × 10^−6^	5.217 × 10^−6^	1.734 × 10^−6^
	Rank	2	15	14	1	8	13	5	11
F10	Best	**1.244 × 10^4^**	3.075 × 10^4^	2.712 × 10^4^	2.978 × 10^4^	2.144 × 10^4^	3.086 × 10^4^	1.370 × 10^4^	2.933 × 10^4^
	Mean	**1.551 × 10^4^**	3.228 × 10^4^	2.822 × 10^4^	3.144 × 10^4^	2.461 × 10^4^	3.226 × 10^4^	1.757 × 10^4^	3.131 × 10^4^
	Std	1.531 × 10^3^	5.730 × 10^2^	6.137 × 10^2^	6.969 × 10^2^	2.013 × 10^3^	**5.340 × 10^2^**	2.049 × 10^3^	1.164 × 10^3^
	P	~	1.734 × 10^−6^	1.734 × 10^−6^	1.734 × 10^−6^	1.734 × 10^−6^	1.734 × 10^−6^	3.881 × 10^−4^	1.734 × 10^−6^
	Rank	1	14	10	12	7	15	2	13
F11	Best	**2.568 × 10^3^**	1.211 × 10^5^	9.581 × 10^4^	3.420 × 10^5^	4.620 × 10^4^	7.112 × 10^4^	2.163 × 10^4^	1.702 × 10^5^
	Mean	**3.409 × 10^3^**	2.615 × 10^5^	1.324 × 10^5^	4.596 × 10^5^	1.109 × 10^5^	1.082 × 10^5^	1.546 × 10^5^	2.637 × 10^5^
	Std	2.973 × 10^2^	7.202 × 10^4^	1.855 × 10^4^	6.170 × 10^4^	5.685 × 10^4^	1.654 × 10^4^	8.149 × 10^4^	7.681 × 10^4^
	P	~	1.734 × 10^−6^	1.734 × 10^−6^	1.734 × 10^−6^	1.734 × 10^−6^	1.734 × 10^−6^	1.734 × 10^−6^	1.734 × 10^−6^
	Rank	1	13	10	15	6	7	11	14
F12	Best	1.911 × 10^8^	6.500 × 10^10^	1.486 × 10^10^	3.235 × 10^10^	1.213 × 10^9^	5.466 × 10^10^	1.608 × 10^10^	1.242 × 10^11^
	Mean	5.108 × 10^8^	9.856 × 10^10^	1.938 × 10^10^	5.108 × 10^10^	2.582 × 10^9^	7.371 × 10^10^	4.216 × 10^10^	1.696 × 10^11^
	Std	2.350 × 10^8^	1.679 × 10^10^	2.743 × 10^9^	1.043 × 10^10^	6.116 × 10^8^	1.085 × 10^10^	2.057 × 10^10^	2.141 × 10^10^
	P	~	1.734 × 10^−6^	1.734 × 10^−6^	1.734 × 10^−6^	1.734 × 10^−6^	1.734 × 10^−6^	1.734 × 10^−6^	1.734 × 10^−6^
	Rank	2	12	6	10	4	11	8	15
F13	Best	**8.410 × 10^4^**	8.568 × 10^9^	6.137 × 10^7^	6.194 × 10^9^	3.429 × 10^6^	8.176 × 10^9^	3.355 × 10^8^	2.171 × 10^10^
	Mean	**3.362 × 10^5^**	1.726 × 10^10^	1.501 × 10^8^	1.112 × 10^10^	1.966 × 10^7^	1.294 × 10^10^	6.719 × 10^9^	3.710 × 10^10^
	Std	2.982 × 10^5^	4.772 × 10^9^	4.411 × 10^7^	2.286 × 10^9^	1.577 × 10^7^	3.141 × 10^9^	5.838 × 10^9^	9.290 × 10^9^
	P	~	1.734 × 10^−6^	1.734 × 10^−6^	1.734 × 10^−6^	1.734 × 10^−6^	1.734 × 10^−6^	1.734 × 10^−6^	1.734 × 10^−6^
	Rank	1	12	6	10	4	11	8	14
F14	Best	4.693 × 10^5^	1.940 × 10^7^	1.768 × 10^7^	3.927 × 10^7^	2.452 × 10^6^	1.471 × 10^7^	8.605 × 10^5^	1.149 × 10^7^
	Mean	1.439 × 10^6^	5.642 × 10^7^	3.283 × 10^7^	1.034 × 10^8^	9.171 × 10^6^	2.964 × 10^7^	9.754 × 10^6^	4.165 × 10^7^
	Std	7.837 × 10^5^	2.074 × 10^7^	1.002 × 10^7^	4.660 × 10^7^	4.371 × 10^6^	1.224 × 10^7^	9.873 × 10^6^	3.054 × 10^7^
	P	~	1.734 × 10^−6^	1.734 × 10^−6^	1.734 × 10^−6^	1.921 × 10^−6^	1.734 × 10^−6^	2.353 × 10^−6^	1.734 × 10^−6^
	Rank	2	14	11	15	6	10	5	12
F15	Best	**4.205 × 10^4^**	3.712 × 10^9^	2.226 × 10^6^	3.304 × 10^9^	4.680 × 10^5^	1.806 × 10^9^	1.904 × 10^5^	3.436 × 10^9^
	Mean	**1.156 × 10^5^**	6.346 × 10^9^	1.602 × 10^7^	5.342 × 10^9^	7.800 × 10^6^	4.074 × 10^9^	2.011 × 10^9^	1.603 × 10^10^
	Std	**5.092 × 10^4^**	1.707 × 10^9^	1.241 × 10^7^	1.021 × 10^9^	2.912 × 10^7^	1.048 × 10^9^	1.366 × 10^9^	5.571 × 10^9^
	P	~	1.734 × 10^−6^	1.734 × 10^−6^	1.734 × 10^−6^	1.734 × 10^−6^	1.734 × 10^−6^	1.734 × 10^−6^	1.734 × 10^−6^
	Rank	1	12	6	11	4	10	8	14
F16	Best	**4.258 × 10^3^**	1.190 × 10^4^	9.668 × 10^3^	1.228 × 10^4^	7.083 × 10^3^	1.142 × 10^4^	6.455 × 10^3^	1.698 × 10^4^
	Mean	**6.006 × 10^3^**	1.390 × 10^4^	1.043 × 10^4^	1.356 × 10^4^	1.304 × 10^4^	1.370 × 10^4^	8.287 × 10^3^	2.214 × 10^4^
	Std	7.028 × 10^2^	1.174 × 10^3^	**3.721 × 10^2^**	6.291 × 10^2^	2.020 × 10^3^	7.926 × 10^2^	7.242 × 10^2^	2.727 × 10^3^
	P	~	1.734 × 10^−6^	1.734 × 10^−6^	1.734 × 10^−6^	1.734 × 10^−6^	1.734 × 10^−6^	1.921 × 10^−6^	1.734 × 10^−6^
	Rank	1	11	6	10	9	12	4	15
F17	Best	**3.858 × 10^3^**	1.581 × 10^4^	7.043 × 10^3^	1.424 × 10^4^	6.312 × 10^3^	1.129 × 10^4^	6.605 × 10^3^	1.297 × 10^4^
	Mean	**4.873 × 10^3^**	1.074 × 10^5^	7.768 × 10^3^	3.431 × 10^4^	7.879 × 10^3^	2.134 × 10^4^	1.301 × 10^4^	3.196 × 10^6^
	Std	5.700 × 10^2^	1.492 × 10^5^	**3.097 × 10^2^**	1.888 × 10^4^	1.284 × 10^3^	2.088 × 10^4^	1.361 × 10^4^	6.929 × 10^6^
	P	~	1.734 × 10^−6^	1.734 × 10^−6^	1.734 × 10^−6^	1.734 × 10^−6^	1.734 × 10^−6^	1.734 × 10^−6^	1.734 × 10^−6^
	Rank	1	12	6	11	4	9	8	14
F18	Best	**5.024 × 10^5^**	4.500 × 10^7^	2.498 × 10^7^	6.175 × 10^7^	1.311 × 10^6^	2.844 × 10^7^	1.115 × 10^6^	1.237 × 10^7^
	Mean	2.188 × 10^6^	8.750 × 10^7^	4.840 × 10^7^	1.378 × 10^8^	4.976 × 10^6^	6.519 × 10^7^	2.823 × 10^7^	8.460 × 10^7^
	Std	1.113 × 10^6^	3.241 × 10^7^	1.247 × 10^7^	3.842 × 10^7^	2.133 × 10^6^	2.825 × 10^7^	4.039 × 10^7^	7.272 × 10^7^
	P	~	1.734 × 10^−6^	1.734 × 10^−6^	1.734 × 10^−6^	4.286 × 10^−6^	1.734 × 10^−6^	1.973 × 10^−5^	1.734 × 10^−6^
	Rank	2	13	10	15	4	11	7	12
F19	Best	**8.691 × 10^5^**	3.078 × 10^9^	2.012 × 10^7^	3.311 × 10^9^	5.638 × 10^6^	2.081 × 10^9^	1.091 × 10^7^	5.271 × 10^9^
	Mean	1.177 × 10^7^	7.168 × 10^9^	3.787 × 10^7^	5.110 × 10^9^	4.048 × 10^7^	3.052 × 10^9^	2.062 × 10^9^	1.623 × 10^10^
	Std	6.415 × 10^6^	3.064 × 10^9^	1.188 × 10^7^	1.051 × 10^9^	2.549 × 10^7^	7.798 × 10^8^	2.171 × 10^9^	5.768 × 10^9^
	P	~	1.734 × 10^−6^	1.734 × 10^−6^	1.734 × 10^−6^	2.163 × 10^−5^	1.734 × 10^−6^	1.734 × 10^−6^	1.734 × 10^−6^
	Rank	3	12	6	11	5	10	8	14
F20	Best	**4.046 × 10^3^**	7.059 × 10^3^	5.847 × 10^3^	6.088 × 10^3^	5.121 × 10^3^	7.021 × 10^3^	4.709 × 10^3^	6.670 × 10^3^
	Mean	**5.067 × 10^3^**	7.860 × 10^3^	6.420 × 10^3^	6.932 × 10^3^	6.527 × 10^3^	7.686 × 10^3^	5.847 × 10^3^	7.503 × 10^3^
	Std	5.223 × 10^2^	3.278 × 10^2^	**2.244 × 10^2^**	4.845 × 10^2^	4.875 × 10^2^	3.257 × 10^2^	5.697 × 10^2^	4.570 × 10^2^
	P	~	1.734 × 10^−6^	1.734 × 10^−6^	1.734 × 10^−6^	1.734 × 10^−6^	1.734 × 10^−6^	1.057 × 10^−4^	1.734 × 10^−6^
	Rank	1	15	7	11	10	14	3	13
F21	Best	2.780 × 10^3^	4.192 × 10^3^	3.710 × 10^3^	**2.427 × 10^3^**	3.713 × 10^3^	3.849 × 10^3^	3.476 × 10^3^	4.271 × 10^3^
	Mean	**2.998 × 10^3^**	4.403 × 10^3^	3.853 × 10^3^	3.213 × 10^3^	4.161 × 10^3^	4.038 × 10^3^	3.733 × 10^3^	4.787 × 10^3^
	Std	9.322 × 10^1^	1.215 × 10^2^	**4.108 × 10^1^**	5.439 × 10^2^	2.437 × 10^2^	8.944 × 10^1^	1.773 × 10^2^	2.712 × 10^2^
	P	~	1.734 × 10^−6^	1.734 × 10^−6^	1.566 × 10^−2^	1.734 × 10^−6^	1.734 × 10^−6^	1.734 × 10^−6^	1.734 × 10^−6^
	Rank	1	13	7	3	9	8	5	15
F22	Best	**2.344 × 10^3^**	3.197 × 10^4^	2.810 × 10^4^	3.283 × 10^4^	2.520 × 10^4^	3.332 × 10^4^	1.685 × 10^4^	3.136 × 10^4^
	Mean	**1.766 × 10^4^**	3.402 × 10^4^	2.993 × 10^4^	3.391 × 10^4^	2.793 × 10^4^	3.459 × 10^4^	2.040 × 10^4^	3.415 × 10^4^
	Std	5.488 × 10^3^	7.492 × 10^2^	6.252 × 10^2^	**5.254 × 10^2^**	1.907 × 10^3^	5.593 × 10^2^	1.808 × 10^3^	1.510 × 10^3^
	P	~	1.734 × 10^−6^	1.734 × 10^−6^	1.734 × 10^−6^	1.734 × 10^−6^	1.734 × 10^−6^	9.842 × 10^−3^	1.734 × 10^−6^
	Rank	1	13	9	12	7	15	2	14
F23	Best	**3.342 × 10^3^**	4.941 × 10^3^	3.718 × 10^3^	4.107 × 10^3^	4.392 × 10^3^	4.692 × 10^3^	3.660 × 10^3^	5.537 × 10^3^
	Mean	**3.594 × 10^3^**	5.405 × 10^3^	3.811 × 10^3^	4.176 × 10^3^	4.988 × 10^3^	5.004 × 10^3^	3.892 × 10^3^	6.491 × 10^3^
	Std	5.960 × 10^2^	2.308 × 10^2^	**3.756 × 10^1^**	4.205 × 10^1^	2.564 × 10^2^	1.115 × 10^2^	1.194 × 10^2^	6.505 × 10^2^
	P	~	1.921 × 10^−6^	3.405 × 10^−5^	3.112 × 10^−5^	1.973 × 10^−5^	1.238 × 10^−5^	3.112 × 10^−5^	1.734 × 10^−6^
	Rank	1	11	2	5	9	8	4	15
F24	Best	**3.687 × 10^3^**	6.040 × 10^3^	4.306 × 10^3^	4.703 × 10^3^	5.373 × 10^3^	6.208 × 10^3^	4.256 × 10^3^	7.781 × 10^3^
	Mean	**3.960 × 10^3^**	6.823 × 10^3^	4.384 × 10^3^	4.865 × 10^3^	6.279 × 10^3^	6.824 × 10^3^	4.506 × 10^3^	9.725 × 10^3^
	Std	2.154 × 10^2^	3.804 × 10^2^	**3.387 × 10^1^**	9.078 × 10^1^	4.452 × 10^2^	2.820 × 10^2^	1.492 × 10^2^	1.040 × 10^3^
	P	~	1.734 × 10^−6^	5.217 × 10^−6^	1.734 × 10^−6^	1.734 × 10^−6^	1.734 × 10^−6^	3.182 × 10^−6^	1.734 × 10^−6^
	Rank	1	9	2	4	8	10	3	15
F25	Best	3.293 × 10^3^	5.029 × 10^4^	2.914 × 10^4^	4.477 × 10^3^	4.379 × 10^3^	1.455 × 10^4^	6.581 × 10^3^	2.147 × 10^4^
	Mean	3.417 × 10^3^	6.552 × 10^4^	3.480 × 10^4^	6.484 × 10^3^	4.784 × 10^3^	1.743 × 10^4^	1.477 × 10^4^	2.610 × 10^4^
	Std	**5.368 × 10^1^**	8.724 × 10^3^	3.029 × 10^3^	2.155 × 10^3^	2.381 × 10^2^	1.978 × 10^3^	8.267 × 10^3^	2.439 × 10^3^
	P	~	1.734 × 10^−6^	1.734 × 10^−6^	1.734 × 10^−6^	1.734 × 10^−6^	1.734 × 10^−6^	1.734 × 10^−6^	1.734 × 10^−6^
	Rank	2	15	14	5	4	10	9	12
F26	Best	**2.963 × 10^3^**	2.736 × 10^4^	1.756 × 10^4^	2.047 × 10^4^	2.665 × 10^4^	3.295 × 10^4^	1.658 × 10^4^	4.755 × 10^4^
	Mean	**8.717 × 10^3^**	3.431 × 10^4^	1.844 × 10^4^	2.217 × 10^4^	3.384 × 10^4^	3.645 × 10^4^	1.947 × 10^4^	5.189 × 10^4^
	Std	4.981 × 10^3^	3.305 × 10^3^	**4.000 × 10^2^**	9.097 × 10^2^	3.171 × 10^3^	2.344 × 10^3^	1.920 × 10^3^	2.764 × 10^3^
	P	~	1.734 × 10^−6^	1.921 × 10^−6^	1.734 × 10^−6^	1.734 × 10^−6^	1.734 × 10^−6^	1.734 × 10^−6^	1.734 × 10^−6^
	Rank	1	10	2	4	9	12	3	14
F27	Best	**3.415 × 10^3^**	4.217 × 10^3^	3.652 × 10^3^	4.451 × 10^3^	4.176 × 10^3^	6.916 × 10^3^	3.581 × 10^3^	7.896 × 10^3^
	Mean	**3.511 × 10^3^**	5.582 × 10^3^	3.830 × 10^3^	4.711 × 10^3^	5.283 × 10^3^	7.612 × 10^3^	4.037 × 10^3^	1.182 × 10^4^
	Std	**6.520 × 10^1^**	8.232 × 10^2^	7.257 × 10^1^	1.590 × 10^2^	6.334 × 10^2^	4.214 × 10^2^	3.027 × 10^2^	1.900 × 10^3^
	P	~	1.734 × 10^−6^	1.734 × 10^−6^	1.734 × 10^−6^	1.734 × 10^−6^	1.734 × 10^−6^	1.734 × 10^−6^	1.734 × 10^−6^
	Rank	1	8	2	6	7	12	3	15
F28	Best	3.401 × 10^3^	2.265 × 10^4^	1.574 × 10^4^	1.884 × 10^4^	4.909 × 10^3^	1.925 × 10^4^	1.127 × 10^4^	2.024 × 10^4^
	Mean	3.484 × 10^3^	2.794 × 10^4^	1.598 × 10^4^	2.125 × 10^4^	5.840 × 10^3^	2.299 × 10^4^	1.964 × 10^4^	2.843 × 10^4^
	Std	3.742 × 10^1^	3.669 × 10^3^	1.235 × 10^2^	1.038 × 10^3^	5.316 × 10^2^	1.858 × 10^3^	2.669 × 10^3^	2.561 × 10^3^
	P	~	1.734 × 10^−6^	1.734 × 10^−6^	1.734 × 10^−6^	1.734 × 10^−6^	1.734 × 10^−6^	1.734 × 10^−6^	1.734 × 10^−6^
	Rank	2	13	8	10	4	11	9	14
F29	Best	6.360 × 10^3^	1.756 × 10^4^	8.230 × 10^3^	**5.227 × 10^3^**	1.122 × 10^4^	1.525 × 10^4^	7.796 × 10^3^	3.733 × 10^4^
	Mean	**8.009 × 10^3^**	4.892 × 10^4^	9.182 × 10^3^	1.411 × 10^4^	1.558 × 10^4^	2.039 × 10^4^	1.293 × 10^4^	2.822 × 10^5^
	Std	6.860 × 10^2^	5.556 × 10^4^	**3.717 × 10^2^**	1.044 × 10^4^	1.892 × 10^3^	4.452 × 10^3^	9.812 × 10^3^	2.770 × 10^5^
	P	~	1.734 × 10^−6^	2.603 × 10^−6^	1.986 × 10^−1^	1.734 × 10^−6^	1.734 × 10^−6^	2.353 × 10^−6^	1.734 × 10^−6^
	Rank	1	12	2	5	9	11	6	15
F30	Best	3.756 × 10^7^	5.450 × 10^9^	**1.397 × 10^6^**	7.158 × 10^9^	2.111 × 10^8^	5.964 × 10^9^	3.437 × 10^7^	1.471 × 10^10^
	Mean	8.772 × 10^7^	1.110 × 10^10^	**7.966 × 10^6^**	9.796 × 10^9^	7.435 × 10^8^	8.809 × 10^9^	2.927 × 10^9^	3.077 × 10^10^
	Std	3.338 × 10^7^	4.431 × 10^9^	1.798 × 10^7^	1.584 × 10^9^	3.255 × 10^8^	1.447 × 10^9^	2.482 × 10^9^	8.176 × 10^9^
	P	~	1.734 × 10^−6^	1.734 × 10^−6^	1.734 × 10^−6^	1.734 × 10^−6^	1.734 × 10^−6^	1.921 × 10^−6^	1.734 × 10^−6^
	Rank	3	12	1	11	6	10	7	14
Mean Rank		**1.5**	12.9	7.4	7.8	6.7	10.8	6.8	13.6
+/=/−		~	29/0/0	28/0/1	22/2/4	29/0/0	29/0/0	29/0/0	29/0/0

**Table A15:** Comparison results with newly proposed algorithms on CEC2017 (Dim = 100).

Function	Index	MShOA	GJO	WAA	SHO	HO	PO	AO
F1	Best	2.091 × 10^11^	1.070 × 10^11^	2.934 × 10^7^	1.834 × 10^11^	6.863 × 10^10^	2.375 × 10^10^	7.274 × 10^8^
	Mean	2.617 × 10^11^	1.335 × 10^11^	**3.530 × 10^7^**	2.079 × 10^11^	8.173 × 10^10^	4.370 × 10^10^	1.255 × 10^9^
	Std	2.036 × 10^10^	1.131 × 10^10^	**3.909 × 10^6^**	1.253 × 10^10^	7.100 × 10^9^	1.180 × 10^10^	3.037 × 10^8^
	P	1.734 × 10^−6^	1.734 × 10^−6^	2.127 × 10^−6^	1.734 × 10^−6^	1.734 × 10^−6^	1.734 × 10^−6^	1.734 × 10^−6^
	Rank	14	9	2	12	7	6	4
F3	Best	3.318 × 10^5^	2.478 × 10^5^	1.446 × 10^5^	2.807 × 10^5^	2.879 × 10^5^	1.828 × 10^5^	2.691 × 10^5^
	Mean	3.561 × 10^5^	2.773 × 10^5^	1.992 × 10^5^	3.148 × 10^5^	3.130 × 10^5^	2.109 × 10^5^	3.103 × 10^5^
	Std	1.380 × 10^4^	1.676 × 10^4^	2.626 × 10^4^	1.215 × 10^4^	**8.553 × 10^3^**	1.591 × 10^4^	2.098 × 10^4^
	P	1.734 × 10^−6^	1.734 × 10^−6^	5.752 × 10^−6^	1.734 × 10^−6^	1.734 × 10^−6^	1.734 × 10^−6^	1.734 × 10^−6^
	Rank	8	4	2	7	6	3	5
F4	Best	6.225 × 10^4^	1.156 × 10^4^	**6.212 × 10^2^**	6.605 × 10^2^	9.566 × 10^3^	2.637 × 10^3^	1.041 × 10^3^
	Mean	9.315 × 10^4^	1.858 × 10^4^	**6.981 × 10^2^**	4.715 × 10^4^	1.409 × 10^4^	4.308 × 10^3^	1.299 × 10^3^
	Std	2.321 × 10^4^	3.898 × 10^3^	**3.506 × 10^1^**	7.983 × 10^3^	3.136 × 10^3^	1.028 × 10^3^	1.484 × 10^2^
	P	1.734 × 10^−6^	1.734 × 10^−6^	5.287 × 10^−4^	1.734 × 10^−6^	1.734 × 10^−6^	1.734 × 10^−6^	1.734 × 10^−6^
	Rank	15	8	1	12	6	5	3
F5	Best	1.760 × 10^3^	1.418 × 10^3^	1.365 × 10^3^	9.560 × 10^2^	1.668 × 10^3^	1.449 × 10^3^	1.235 × 10^3^
	Mean	2.058 × 10^3^	1.561 × 10^3^	1.423 × 10^3^	1.883 × 10^3^	1.796 × 10^3^	1.596 × 10^3^	1.399 × 10^3^
	Std	1.164 × 10^2^	1.213 × 10^2^	4.027 × 10^1^	5.396 × 10^1^	5.762 × 10^1^	8.337 × 10^1^	7.704 × 10^1^
	P	1.734 × 10^−6^	2.603 × 10^−6^	1.127 × 10^−5^	1.734 × 10^−6^	1.734 × 10^−6^	1.734 × 10^−6^	4.072 × 10^−5^
	Rank	13	5	4	10	8	6	3
F6	Best	6.949 × 10^2^	6.619 × 10^2^	6.675 × 10^2^	6.429 × 10^2^	6.843 × 10^2^	6.759 × 10^2^	6.649 × 10^2^
	Mean	7.101 × 10^2^	6.715 × 10^2^	6.729 × 10^2^	: 697.5236	6.964 × 10^2^	6.843 × 10^2^	6.735 × 10^2^
	Std	5.819 × 10^0^	6.867 × 10^0^	3.032 × 10^0^	3.116 × 10^0^	4.087 × 10^0^	4.152 × 10^0^	5.711 × 10^0^
	P	1.734 × 10^−6^	1.238 × 10^−5^	2.879 × 10^−6^	1.734 × 10^−6^	1.734 × 10^−6^	1.734 × 10^−6^	3.882 × 10^−6^
	Rank	13	4	6	12	11	8	5
F7	Best	3.688 × 10^3^	2.618 × 10^3^	3.356 × 10^3^	1.483 × 10^3^	3.470 × 10^3^	3.262 × 10^3^	2.428 × 10^3^
	Mean	3.958 × 10^3^	2.877 × 10^3^	3.482 × 10^3^	3.643 × 10^3^	3.720 × 10^3^	3.433 × 10^3^	2.906 × 10^3^
	Std	1.143 × 10^2^	1.325 × 10^2^	8.242 × 10^1^	8.338 × 10^1^	8.085 × 10^1^	9.286 × 10^1^	2.203 × 10^2^
	P	1.734 × 10^−6^	1.734 × 10^−6^	1.734 × 10^−6^	1.734 × 10^−6^	1.734 × 10^−6^	1.734 × 10^−6^	1.734 × 10^−6^
	Rank	11	3	6	8	10	5	4
F8	Best	6.048 × 10^4^	3.051 × 10^4^	3.526 × 10^4^	2.437 × 10^4^	4.669 × 10^4^	3.303 × 10^4^	4.227 × 10^4^
	Mean	2.534 × 10^3^	1.864 × 10^3^	1.903 × 10^3^	2.326 × 10^3^	2.254 × 10^3^	2.039 × 10^3^	1.790 × 10^3^
	Std	9.147 × 10^1^	9.006 × 10^1^	**3.440 × 10^1^**	4.449 × 10^1^	5.514 × 10^1^	8.848 × 10^1^	8.630 × 10^1^
	P	1.734 × 10^−6^	1.734 × 10^−6^	1.734 × 10^−6^	1.734 × 10^−6^	1.734 × 10^−6^	1.734 × 10^−6^	1.734 × 10^−6^
	Rank	13	3	5	12	9	6	2
F9	Best	2.820 × 10^4^	1.736 × 10^4^	1.563 × 10^4^	1.244 × 10^4^	2.331 × 10^4^	1.899 × 10^4^	1.690 × 10^4^
	Mean	7.547 × 10^4^	5.209 × 10^4^	4.338 × 10^4^	5.656 × 10^4^	5.617 × 10^4^	4.061 × 10^4^	5.164 × 10^4^
	Std	7.240 × 10^3^	1.322 × 10^4^	**4.169 × 10^3^**	4.852 × 10^3^	5.072 × 10^3^	5.407 × 10^3^	6.636 × 10^3^
	P	1.734 × 10^−6^	5.792 × 10^−5^	1.477 × 10^−4^	1.734 × 10^−6^	1.734 × 10^−6^	7.731 × 10^−3^	1.921 × 10^−6^
	Rank	12	6	4	9	10	3	7
F10	Best	2.820 × 10^4^	1.736 × 10^4^	1.563 × 10^4^	1.244 × 10^4^	2.331 × 10^4^	1.899 × 10^4^	1.690 × 10^4^
	Mean	3.070 × 10^4^	2.378 × 10^4^	1.760 × 10^4^	2.759 × 10^4^	2.621 × 10^4^	2.243 × 10^4^	2.029 × 10^4^
	Std	1.642 × 10^3^	5.030 × 10^3^	1.183 × 10^3^	1.473 × 10^3^	1.781 × 10^3^	2.245 × 10^3^	1.990 × 10^3^
	P	1.734 × 10^−6^	1.921 × 10^−6^	2.843 × 10^−5^	1.734 × 10^−6^	1.734 × 10^−6^	1.734 × 10^−6^	2.603 × 10^−6^
	Rank	11	6	3	9	8	5	4
F11	Best	1.222 × 10^5^	4.545 × 10^4^	2.869 × 10^3^	1.038 × 10^5^	9.917 × 10^4^	1.287 × 10^4^	6.813 × 10^4^
	Mean	1.763 × 10^5^	8.366 × 10^4^	3.523 × 10^3^	1.214 × 10^5^	1.291 × 10^5^	2.558 × 10^4^	9.803 × 10^4^
	Std	2.780 × 10^4^	2.045 × 10^4^	**2.602 × 10^2^**	1.264 × 10^4^	1.559 × 10^4^	7.174 × 10^3^	2.047 × 10^4^
	P	1.734 × 10^−6^	1.734 × 10^−6^	2.452 × 10^−1^	1.734 × 10^−6^	1.734 × 10^−6^	1.734 × 10^−6^	1.734 × 10^−6^
	Rank	12	4	2	8	9	3	5
F12	Best	9.820 × 10^10^	2.643 × 10^10^	**7.565 × 10^7^**	1.911 × 10^8^	1.431 × 10^10^	1.068 × 10^9^	3.020 × 10^8^
	Mean	1.697 × 10^11^	4.644 × 10^10^	**1.013 × 10^8^**	1.174 × 10^11^	2.510 × 10^10^	5.220 × 10^9^	8.020 × 10^8^
	Std	3.570 × 10^10^	1.187 × 10^10^	**1.595 × 10^7^**	1.432 × 10^10^	7.558 × 10^9^	2.856 × 10^9^	2.985 × 10^8^
	P	1.734 × 10^−6^	1.734 × 10^−6^	1.734 × 10^−6^	1.734 × 10^−6^	1.734 × 10^−6^	1.734 × 10^−6^	8.307 × 10^−4^
	Rank	14	9	1	13	7	5	3
F13	Best	1.488 × 10^10^	2.438 × 10^9^	4.756 × 10^5^	8.410 × 10^4^	1.401 × 10^9^	3.417 × 10^6^	1.757 × 10^6^
	Mean	4.319 × 10^10^	8.440 × 10^9^	7.105 × 10^5^	2.247 × 10^10^	3.067 × 10^9^	2.362 × 10^8^	7.606 × 10^6^
	Std	1.075 × 10^10^	2.821 × 10^9^	**1.398 × 10^5^**	5.308 × 10^9^	1.136 × 10^9^	2.757 × 10^8^	9.305 × 10^6^
	P	1.734 × 10^−6^	1.734 × 10^−6^	7.514 × 10^−5^	1.734 × 10^−6^	1.734 × 10^−6^	1.734 × 10^−6^	1.734 × 10^−6^
	Rank	15	9	2	13	7	5	3
F14	Best	1.900 × 10^7^	1.921 × 10^6^	**2.964 × 10^5^**	4.693 × 10^5^	4.302 × 10^6^	2.509 × 10^6^	3.057 × 10^6^
	Mean	7.415 × 10^7^	1.262 × 10^7^	**4.954 × 10^5^**	1.619 × 10^7^	1.856 × 10^7^	6.246 × 10^6^	7.934 × 10^6^
	Std	7.171 × 10^7^	7.990 × 10^6^	**1.156 × 10^5^**	7.305 × 10^6^	7.668 × 10^6^	2.261 × 10^6^	2.436 × 10^6^
	P	1.734 × 10^−6^	1.734 × 10^−6^	2.127 × 10^−6^	1.734 × 10^−6^	1.734 × 10^−6^	2.127 × 10^−6^	1.734 × 10^−6^
	Rank	13	7	1	8	9	3	4
F15	Best	2.542 × 10^9^	1.428 × 10^8^	1.096 × 10^5^	4.205 × 10^4^	1.429 × 10^8^	5.289 × 10^5^	4.463 × 10^5^
	Mean	2.020 × 10^10^	2.629 × 10^9^	2.159 × 10^5^	9.474 × 10^9^	7.563 × 10^8^	2.925 × 10^7^	1.338 × 10^6^
	Std	8.328 × 10^9^	2.337 × 10^9^	6.017 × 10^4^	3.266 × 10^9^	3.367 × 10^8^	4.040 × 10^7^	5.096 × 10^5^
	P	1.734 × 10^−6^	1.734 × 10^−6^	8.466 × 10^−6^	1.734 × 10^−6^	1.734 × 10^−6^	1.734 × 10^−6^	1.734 × 10^−6^
	Rank	15	9	2	13	7	5	3
F16	Best	1.408 × 10^4^	5.894 × 10^3^	6.465 × 10^3^	4.258 × 10^3^	9.881 × 10^3^	8.544 × 10^3^	6.453 × 10^3^
	Mean	1.942 × 10^4^	9.192 × 10^3^	7.785 × 10^3^	1.437 × 10^4^	1.245 × 10^4^	1.068 × 10^4^	7.862 × 10^3^
	Std	2.828 × 10^3^	1.317 × 10^3^	8.622 × 10^2^	1.490 × 10^3^	1.217 × 10^3^	1.163 × 10^3^	8.849 × 10^2^
	P	1.734 × 10^−6^	1.734 × 10^−6^	2.127 × 10^−6^	1.734 × 10^−6^	1.734 × 10^−6^	1.734 × 10^−6^	2.353 × 10^−6^
	Rank	14	5	2	13	8	7	3
F17	Best	1.790 × 10^4^	6.182 × 10^3^	4.908 × 10^3^	3.858 × 10^3^	8.901 × 10^3^	5.679 × 10^3^	4.856 × 10^3^
	Mean	5.688 × 10^6^	1.073 × 10^4^	6.153 × 10^3^	3.215 × 10^5^	2.875 × 10^4^	8.263 × 10^3^	6.328 × 10^3^
	Std	8.622 × 10^6^	7.960 × 10^3^	8.497 × 10^2^	4.174 × 10^5^	2.691 × 10^4^	2.235 × 10^3^	7.249 × 10^2^
	P	1.734 × 10^−6^	1.734 × 10^−6^	6.984 × 10^−6^	1.734 × 10^−6^	1.734 × 10^−6^	1.921 × 10^−6^	1.921 × 10^−6^
	Rank	15	7	2	13	10	5	3
F18	Best	2.600 × 10^7^	1.769 × 10^6^	8.099 × 10^5^	5.024 × 10^5^	6.772 × 10^6^	1.284 × 10^6^	2.245 × 10^6^
	Mean	1.742 × 10^8^	1.404 × 10^7^	**1.759 × 10^6^**	1.987 × 10^7^	1.951 × 10^7^	4.839 × 10^6^	6.765 × 10^6^
	Std	2.244 × 10^8^	1.106 × 10^7^	**7.890 × 10^5^**	1.129 × 10^7^	7.211 × 10^6^	2.776 × 10^6^	2.334 × 10^6^
	P	1.734 × 10^−6^	2.879 × 10^−6^	1.414 × 10^−1^	1.734 × 10^−6^	1.734 × 10^−6^	3.405 × 10^−5^	1.734 × 10^−6^
	Rank	14	6	1	8	9	3	5
F19	Best	7.984 × 10^9^	9.428 × 10^7^	1.081 × 10^6^	8.691 × 10^5^	3.517 × 10^8^	1.608 × 10^6^	2.426 × 10^6^
	Mean	2.009 × 10^10^	1.746 × 10^9^	**3.081 × 10^6^**	8.478 × 10^9^	1.189 × 10^9^	3.466 × 10^7^	9.432 × 10^6^
	Std	7.237 × 10^9^	1.247 × 10^9^	**1.549 × 10^6^**	3.110 × 10^9^	5.428 × 10^8^	4.437 × 10^7^	5.549 × 10^6^
	P	1.734 × 10^−6^	1.734 × 10^−6^	4.286 × 10^−6^	1.734 × 10^−6^	1.734 × 10^−6^	3.162 × 10^−3^	7.865 × 10^−2^
	Rank	15	9	1	13	7	4	2
F20	Best	6.515 × 10^3^	4.793 × 10^3^	5.263 × 10^3^	4.046 × 10^3^	5.308 × 10^3^	5.358 × 10^3^	4.198 × 10^3^
	Mean	7.437 × 10^3^	6.303 × 10^3^	6.516 × 10^3^	6.289 × 10^3^	6.509 × 10^3^	6.123 × 10^3^	5.566 × 10^3^
	Std	4.387 × 10^2^	9.524 × 10^2^	5.590 × 10^2^	5.909 × 10^2^	5.561 × 10^2^	4.080 × 10^2^	6.640 × 10^2^
	P	1.734 × 10^−6^	3.724 × 10^−5^	2.879 × 10^−6^	3.182 × 10^−6^	1.921 × 10^−6^	3.882 × 10^−6^	3.609 × 10^−3^
	Rank	12	6	9	5	8	4	2
F21	Best	4.118 × 10^3^	3.204 × 10^3^	4.046 × 10^3^	2.780 × 10^3^	3.953 × 10^3^	3.456 × 10^3^	3.056 × 10^3^
	Mean	4.392 × 10^3^	3.449 × 10^3^	4.474 × 10^3^	4.261 × 10^3^	4.175 × 10^3^	3.754 × 10^3^	3.322 × 10^3^
	Std	1.741 × 10^2^	1.357 × 10^2^	2.128 × 10^2^	1.169 × 10^2^	1.373 × 10^2^	1.705 × 10^2^	1.229 × 10^2^
	P	1.734 × 10^−6^	1.734 × 10^−6^	1.734 × 10^−6^	1.734 × 10^−6^	1.734 × 10^−6^	1.734 × 10^−6^	1.734 × 10^−6^
	Rank	12	4	14	11	10	6	2
F22	Best	3.086 × 10^4^	2.068 × 10^4^	1.829 × 10^4^	2.344 × 10^3^	2.727 × 10^4^	2.371 × 10^4^	2.688 × 10^3^
	Mean	3.341 × 10^4^	2.654 × 10^4^	2.093 × 10^4^	3.149 × 10^4^	2.953 × 10^4^	2.647 × 10^4^	2.170 × 10^4^
	Std	1.161 × 10^3^	4.579 × 10^3^	1.206 × 10^3^	1.136 × 10^3^	1.095 × 10^3^	1.397 × 10^3^	5.377 × 10^3^
	P	1.734 × 10^−6^	3.182 × 10^−6^	3.317 × 10^−4^	1.734 × 10^−6^	1.734 × 10^−6^	1.734 × 10^−6^	3.589 × 10^−4^
	Rank	11	6	3	10	8	5	4
F23	Best	5.057 × 10^3^	4.042 × 10^3^	5.177 × 10^3^	5.491 × 10^3^	4.921 × 10^3^	4.058 × 10^3^	3.627 × 10^3^
	Mean	5.632 × 10^3^	4.297 × 10^3^	6.320 × 10^3^	6.006 × 10^3^	5.287 × 10^3^	4.529 × 10^3^	3.877 × 10^3^
	Std	3.342 × 10^2^	1.361 × 10^2^	8.072 × 10^2^	3.784 × 10^2^	2.420 × 10^2^	2.165 × 10^2^	1.532 × 10^2^
	P	1.921 × 10^−6^	3.112 × 10^−5^	1.734 × 10^−6^	1.921 × 10^−6^	2.603 × 10^−6^	3.112 × 10^−5^	3.112 × 10^−5^
	Rank	12	6	14	13	10	7	3
F24	Best	6.949 × 10^3^	5.230 × 10^3^	6.033 × 10^3^	3.687 × 10^3^	5.731 × 10^3^	5.082 × 10^3^	4.434 × 10^3^
	Mean	8.610 × 10^3^	5.801 × 10^3^	9.038 × 10^3^	9.437 × 10^3^	7.229 × 10^3^	5.731 × 10^3^	4.852 × 10^3^
	Std	1.074 × 10^3^	3.510 × 10^2^	1.893 × 10^3^	5.365 × 10^2^	5.740 × 10^2^	3.589 × 10^2^	2.651 × 10^2^
	P	1.734 × 10^−6^	1.734 × 10^−6^	1.734 × 10^−6^	1.734 × 10^−6^	1.734 × 10^−6^	1.734 × 10^−6^	2.127 × 10^−6^
	Rank	13	6	12	14	11	7	5
F25	Best	2.389 × 10^4^	8.428 × 10^3^	**3.213 × 10^3^**	1.519 × 10^4^	7.880 × 10^3^	4.902 × 10^3^	3.757 × 10^3^
	Mean	2.964 × 10^4^	1.147 × 10^4^	**3.338 × 10^3^**	1.853 × 10^4^	9.369 × 10^3^	6.116 × 10^3^	3.983 × 10^3^
	Std	3.075 × 10^3^	1.455 × 10^3^	6.290 × 10^1^	2.085 × 10^3^	8.833 × 10^2^	6.799 × 10^2^	1.402 × 10^2^
	P	1.734 × 10^−6^	1.734 × 10^−6^	5.792 × 10^−5^	1.734 × 10^−6^	1.734 × 10^−6^	1.734 × 10^−6^	1.734 × 10^−6^
	Rank	13	8	1	11	7	6	3
F26	Best	4.550 × 10^4^	2.390 × 10^4^	2.345 × 10^4^	3.852 × 10^4^	3.009 × 10^4^	2.334 × 10^4^	8.680 × 10^3^
	Mean	5.509 × 10^4^	2.688 × 10^4^	2.672 × 10^4^	4.173 × 10^4^	3.565 × 10^4^	3.218 × 10^4^	2.250 × 10^4^
	Std	5.020 × 10^3^	2.197 × 10^3^	1.466 × 10^3^	2.280 × 10^3^	2.351 × 10^3^	2.916 × 10^3^	5.469 × 10^3^
	P	1.734 × 10^−6^	1.734 × 10^−6^	1.734 × 10^−6^	1.734 × 10^−6^	1.734 × 10^−6^	1.734 × 10^−6^	2.879 × 10^−6^
	Rank	15	7	6	13	11	8	5
F27	Best	6.901 × 10^3^	4.776 × 10^3^	4.837 × 10^3^	8.170 × 10^3^	4.770 × 10^3^	3.852 × 10^3^	3.822 × 10^3^
	Mean	9.767 × 10^3^	5.437 × 10^3^	6.948 × 10^3^	1.013 × 10^4^	5.997 × 10^3^	4.526 × 10^3^	4.340 × 10^3^
	Std	1.523 × 10^3^	4.398 × 10^2^	1.690 × 10^3^	9.019 × 10^2^	6.243 × 10^2^	3.941 × 10^2^	2.823 × 10^2^
	P	1.734 × 10^−6^	1.734 × 10^−6^	1.734 × 10^−6^	1.734 × 10^−6^	1.734 × 10^−6^	1.734 × 10^−6^	1.734 × 10^−6^
	Rank	13	9	11	14	10	5	4
F28	Best	2.573 × 10^4^	1.202 × 10^4^	**3.355 × 10^3^**	2.082 × 10^4^	1.027 × 10^4^	5.586 × 10^3^	3.921 × 10^3^
	Mean	3.493 × 10^4^	1.588 × 10^4^	**3.404 × 10^3^**	2.389 × 10^4^	1.289 × 10^4^	6.599 × 10^3^	4.272 × 10^3^
	Std	3.740 × 10^3^	1.709 × 10^3^	**2.706 × 10^1^**	1.798 × 10^3^	1.460 × 10^3^	7.585 × 10^2^	1.768 × 10^2^
	P	1.734 × 10^−6^	1.734 × 10^−6^	1.734 × 10^−6^	1.734 × 10^−6^	1.734 × 10^−6^	1.734 × 10^−6^	1.734 × 10^−6^
	Rank	15	7	1	12	6	5	3
F29	Best	1.844 × 10^4^	9.582 × 10^3^	8.946 × 10^3^	2.135 × 10^4^	1.457 × 10^4^	1.170 × 10^4^	8.366 × 10^3^
	Mean	7.436 × 10^5^	1.636 × 10^4^	9.964 × 10^3^	4.801 × 10^4^	1.866 × 10^4^	1.403 × 10^4^	1.018 × 10^4^
	Std	1.414 × 10^6^	6.749 × 10^3^	1.269 × 10^3^	3.346 × 10^4^	2.254 × 10^3^	1.509 × 10^3^	1.080 × 10^3^
	P	1.734 × 10^−6^	1.734 × 10^−6^	1.921 × 10^−6^	1.734 × 10^−6^	1.734 × 10^−6^	1.734 × 10^−6^	3.182 × 10^−6^
	Rank	14	8	3	13	10	7	4
F30	Best	1.316 × 10^10^	3.362 × 10^9^	4.945 × 10^6^	8.156 × 10^9^	1.385 × 10^9^	2.004 × 10^8^	5.123 × 10^7^
	Mean	3.406 × 10^10^	7.494 × 10^9^	1.084 × 10^7^	2.031 × 10^10^	4.413 × 10^9^	5.599 × 10^8^	1.337 × 10^8^
	Std	1.089 × 10^10^	2.801 × 10^9^	**3.167 × 10^6^**	5.907 × 10^9^	1.736 × 10^9^	4.308 × 10^8^	6.516 × 10^7^
	P	1.734 × 10^−6^	1.734 × 10^−6^	1.734 × 10^−6^	1.734 × 10^−6^	1.734 × 10^−6^	1.734 × 10^−6^	6.836 × 10^−3^
	Rank	15	9	2	13	8	5	4
Mean Rank		13.2	6.5	4.2	11.1	8.5	5.2	3.7
+/=/−		29/0/0	29/0/0	20/1/8	29/0/0	29/0/0	29/0/0	28/1/0

**Table A16:** Comparison results with classic highly-cited algorithms on CEC2020 (Dim = 10).

Function	Index	EAO	PSO	DE	CMA-ES	WOA	SCA	MFO	HHO
F1	Best	3.044 × 10^4^	4.053 × 10^8^	1.717 × 10^6^	7.609 × 10^7^	1.345 × 10^7^	5.196 × 10^8^	**2.681 × 10^2^**	1.598 × 10^9^
	Mean	1.758 × 10^8^	1.459 × 10^9^	2.572 × 10^7^	4.176 × 10^9^	7.222 × 10^7^	1.344 × 10^9^	1.652 × 10^8^	8.042 × 10^9^
	Std	3.469 × 10^8^	6.407 × 10^8^	1.354 × 10^7^	1.709 × 10^9^	5.301 × 10^7^	6.603 × 10^8^	4.963 × 10^8^	3.705 × 10^9^
	P	2.127 × 10^−6^	4.492 × 10^−2^	1.734 × 10^−6^	8.290 × 10^−1^	2.353 × 10^−6^	7.271 × 10^−3^	1.734 × 10^−6^	2.127 × 10^−6^
	Rank	5	12	4	13	7	10	2	15
F2	Best	1.278 × 10^3^	1.878 × 10^3^	1.760 × 10^3^	2.322 × 10^3^	1.775 × 10^3^	2.096 × 10^3^	1.401 × 10^3^	2.281 × 10^3^
	Mean	**1.849 × 10^3^**	2.576 × 10^3^	2.148 × 10^3^	2.787 × 10^3^	2.414 × 10^3^	2.614 × 10^3^	2.031 × 10^3^	2.675 × 10^3^
	Std	2.988 × 10^2^	2.504 × 10^2^	**1.573 × 10^2^**	1.798 × 10^2^	3.197 × 10^2^	1.921 × 10^2^	3.049 × 10^2^	2.115 × 10^2^
	P	4.286 × 10^−6^	4.196 × 10^−4^	1.734 × 10^−6^	2.370 × 10^−5^	1.734 × 10^−6^	1.852 × 10^−2^	1.734 × 10^−6^	4.286 × 10^−6^
	Rank	1	11	5	15	10	13	4	14
F3	Best	7.212 × 10^2^	7.958 × 10^2^	7.402 × 10^2^	7.306 × 10^2^	7.490 × 10^2^	7.630 × 10^2^	**7.173 × 10^2^**	7.841 × 10^2^
	Mean	**7.359 × 10^2^**	8.412 × 10^2^	7.532 × 10^2^	7.408 × 10^2^	7.987 × 10^2^	7.898 × 10^2^	7.425 × 10^2^	8.126 × 10^2^
	Std	1.144 × 10^1^	2.330 × 10^1^	7.846 × 10^0^	**4.501 × 10^0^**	2.553 × 10^1^	1.723 × 10^1^	1.814 × 10^1^	1.953 × 10^1^
	P	1.734 × 10^−6^	1.973 × 10^−5^	3.160 × 10^−2^	1.734 × 10^−6^	1.734 × 10^−6^	1.020 × 10^−1^	1.734 × 10^−6^	1.734 × 10^−6^
	Rank	1	15	4	2	11	9	3	12
F4	Best	**1.900 × 10^3^**	1.909 × 10^3^	1.904 × 10^3^	2.210 × 10^3^	1.903 × 10^3^	1.912 × 10^3^	1.901 × 10^3^	1.909 × 10^3^
	Mean	**1.902 × 10^3^**	2.023 × 10^3^	1.905 × 10^3^	3.429 × 10^3^	1.912 × 10^3^	2.020 × 10^3^	1.920 × 10^3^	1.341 × 10^4^
	Std	**7.706 × 10^−1^**	2.938 × 10^2^	9.227 × 10^−1^	9.194 × 10^2^	8.749 × 10^0^	2.019 × 10^2^	5.321 × 10^1^	3.305 × 10^4^
	P	1.734 × 10^−6^	1.734 × 10^−6^	1.734 × 10^−6^	1.734 × 10^−6^	1.734 × 10^−6^	3.379 × 10^−3^	1.734 × 10^−6^	1.734 × 10^−6^
	Rank	1	9	4	13	8	10	2	12
F5	Best	**2.290 × 10^3^**	2.386 × 10^4^	3.605 × 10^4^	6.924 × 10^3^	7.735 × 10^3^	8.636 × 10^3^	3.118 × 10^3^	7.901 × 10^3^
	Mean	**7.537 × 10^3^**	2.100 × 10^5^	5.955 × 10^5^	3.919 × 10^5^	3.196 × 10^5^	9.307 × 10^4^	1.683 × 10^5^	4.920 × 10^5^
	Std	**7.133 × 10^3^**	2.422 × 10^5^	6.174 × 10^5^	4.647 × 10^5^	5.977 × 10^5^	1.515 × 10^5^	3.046 × 10^5^	2.595 × 10^5^
	P	1.734 × 10^−6^	1.734 × 10^−6^	2.353 × 10^−6^	2.353 × 10^−6^	5.752 × 10^−6^	2.879 × 10^−6^	1.734 × 10^−6^	1.734 × 10^−6^
	Rank	1	9	13	12	8	6	7	14
F6	Best	1.620 × 10^3^	1.644 × 10^3^	1.644 × 10^3^	1.933 × 10^3^	1.621 × 10^3^	1.728 × 10^3^	**1.603 × 10^3^**	1.650 × 10^3^
	Mean	1.779 × 10^3^	1.823 × 10^3^	**1.746 × 10^3^**	2.023 × 10^3^	1.882 × 10^3^	1.879 × 10^3^	1.777 × 10^3^	1.953 × 10^3^
	Std	**7.345 × 10^1^**	1.266 × 10^2^	8.090 × 10^1^	7.649 × 10^1^	1.367 × 10^2^	7.821 × 10^1^	1.092 × 10^2^	1.689 × 10^2^
	P	1.529 × 10^−1^	1.020 × 10^−1^	1.734 × 10^−6^	2.415 × 10^−3^	3.724 × 10^−5^	9.263 × 10^−1^	3.112 × 10^−5^	1.529 × 10^−1^
	Rank	2	5	1	14	8	10	3	11
F7	Best	2.618 × 10^3^	4.472 × 10^3^	4.789 × 10^3^	9.029 × 10^3^	7.687 × 10^3^	3.545 × 10^3^	3.013 × 10^3^	3.451 × 10^3^
	Mean	**6.858 × 10^3^**	2.122 × 10^4^	4.093 × 10^5^	6.711 × 10^5^	8.295 × 10^5^	1.750 × 10^4^	3.627 × 10^4^	1.208 × 10^6^
	Std	**4.371 × 10^3^**	1.389 × 10^4^	4.322 × 10^5^	8.411 × 10^5^	1.638 × 10^6^	1.128 × 10^4^	5.366 × 10^4^	1.668 × 10^6^
	P	1.494 × 10^−5^	1.734 × 10^−6^	1.734 × 10^−6^	2.603 × 10^−6^	2.843 × 10^−5^	2.225 × 10^−4^	1.734 × 10^−6^	1.494 × 10^−5^
	Rank	1	11	13	15	14	7	9	12
F8	Best	**2.221 × 10^3^**	2.318 × 10^3^	2.343 × 10^3^	2.300 × 10^3^	2.313 × 10^3^	2.312 × 10^3^	2.231 × 10^3^	2.436 × 10^3^
	Mean	**2.304 × 10^3^**	2.490 × 10^3^	2.411 × 10^3^	2.428 × 10^3^	2.594 × 10^3^	2.422 × 10^3^	2.314 × 10^3^	2.934 × 10^3^
	Std	1.576 × 10^1^	2.282 × 10^2^	4.551 × 10^1^	3.323 × 10^2^	5.408 × 10^2^	5.136 × 10^1^	4.890 × 10^1^	4.039 × 10^2^
	P	1.734 × 10^−6^	1.734 × 10^−6^	6.733 × 10^−1^	1.734 × 10^−6^	1.734 × 10^−6^	9.754 × 10^−1^	1.734 × 10^−6^	1.734 × 10^−6^
	Rank	1	12	10	3	7	11	2	15
F9	Best	**2.501 × 10^3^**	2.631 × 10^3^	2.658 × 10^3^	2.801 × 10^3^	2.554 × 10^3^	2.572 × 10^3^	2.673 × 10^3^	2.769 × 10^3^
	Mean	**2.729 × 10^3^**	2.774 × 10^3^	2.765 × 10^3^	2.814 × 10^3^	2.805 × 10^3^	2.783 × 10^3^	2.758 × 10^3^	2.854 × 10^3^
	Std	8.452 × 10^1^	3.967 × 10^1^	3.200 × 10^1^	**7.631 × 10^0^**	7.936 × 10^1^	5.432 × 10^1^	2.024 × 10^1^	5.765 × 10^1^
	P	1.114 × 10^−3^	2.703 × 10^−2^	1.921 × 10^−6^	1.114 × 10^−3^	3.881 × 10^−4^	1.306 × 10^−1^	1.734 × 10^−6^	1.114 × 10^−3^
	Rank	1	7	4	13	11	9	2	14
F10	Best	**2.607 × 10^3^**	2.948 × 10^3^	2.920 × 10^3^	2.944 × 10^3^	2.834 × 10^3^	2.926 × 10^3^	2.898 × 10^3^	3.057 × 10^3^
	Mean	**2.917 × 10^3^**	3.006 × 10^3^	2.959 × 10^3^	3.121 × 10^3^	2.973 × 10^3^	2.986 × 10^3^	2.944 × 10^3^	3.356 × 10^3^
	Std	6.276 × 10^1^	2.383 × 10^1^	**1.327 × 10^1^**	9.548 × 10^1^	4.332 × 10^1^	3.251 × 10^1^	2.499 × 10^1^	2.591 × 10^2^
	P	1.734 × 10^−6^	7.691 × 10^−6^	1.921 × 10^−6^	1.477 × 10^−4^	1.921 × 10^−6^	7.731 × 10^−3^	1.734 × 10^−6^	1.734 × 10^−6^
	Rank	1	12	7	13	8	10	4	15
Mean Rank		**1.5**	10.3	6.5	11.3	9.2	9.5	3.8	13.4
+/=/−		~	9/1/0	8/1/1	9/1/0	9/1/0	10/0/0	5/4/1	10/0/0

**Table A17:** Comparison results with newly proposed algorithms on CEC2020 (Dim = 10).

Function	Index	MShOA	GJO	WAA	SHO	HO	PO	AO
F1	Best	3.676 × 10^8^	3.834 × 10^5^	2.767 × 10^4^	3.301 × 10^8^	1.550 × 10^8^	7.118 × 10^5^	2.248 × 10^6^
	Mean	7.029 × 10^9^	5.175 × 10^8^	**6.819 × 10^4^**	1.705 × 10^9^	1.104 × 10^9^	8.214 × 10^7^	8.788 × 10^7^
	Std	5.418 × 10^9^	7.672 × 10^8^	**2.760 × 10^4^**	1.217 × 10^9^	9.152 × 10^8^	1.513 × 10^8^	1.322 × 10^8^
	P	1.734 × 10^−6^	9.271 × 10^−3^	3.182 × 10^−6^	1.734 × 10^−6^	2.879 × 10^−6^	2.369 × 10^−1^	1.734 × 10^−6^
	Rank	14	8	1	11	9	3	6
F2	Best	1.634 × 10^3^	**1.274 × 10^3^**	1.467 × 10^3^	1.734 × 10^3^	1.358 × 10^3^	1.562 × 10^3^	1.413 × 10^3^
	Mean	2.646 × 10^3^	1.994 × 10^3^	2.227 × 10^3^	2.238 × 10^3^	2.376 × 10^3^	2.127 × 10^3^	2.020 × 10^3^
	Std	3.797 × 10^2^	3.618 × 10^2^	2.235 × 10^2^	2.101 × 10^2^	3.247 × 10^2^	3.132 × 10^2^	2.752 × 10^2^
	P	2.127 × 10^−6^	2.623 × 10^−1^	7.514 × 10^−5^	1.238 × 10^−5^	7.514 × 10^−5^	2.255 × 10^−3^	4.277 × 10^−2^
	Rank	12	2	7	8	9	6	3
F3	Best	7.610 × 10^2^	7.325 × 10^2^	8.115 × 10^2^	7.660 × 10^2^	7.335 × 10^2^	7.350 × 10^2^	7.391 × 10^2^
	Mean	8.193 × 10^2^	7.577 × 10^2^	8.285 × 10^2^	7.898 × 10^2^	7.789 × 10^2^	7.741 × 10^2^	7.693 × 10^2^
	Std	2.916 × 10^1^	1.437 × 10^1^	1.564 × 10^1^	1.215 × 10^1^	1.817 × 10^1^	2.034 × 10^1^	1.703 × 10^1^
	P	1.734 × 10^−6^	3.405 × 10^−5^	1.734 × 10^−6^	1.734 × 10^−6^	1.921 × 10^−6^	3.882 × 10^−6^	1.734 × 10^−6^
	Rank	13	5	14	10	8	7	6
F4	Best	1.908 × 10^3^	1.902 × 10^3^	1.902 × 10^3^	1.910 × 10^3^	1.906 × 10^3^	1.902 × 10^3^	1.903 × 10^3^
	Mean	1.668 × 10^5^	2.096 × 10^3^	1.905 × 10^3^	1.763 × 10^4^	8.118 × 10^3^	1.909 × 10^3^	1.911 × 10^3^
	Std	1.814 × 10^5^	9.650 × 10^2^	2.970 × 10^0^	1.798 × 10^4^	1.087 × 10^4^	4.777 × 10^0^	1.133 × 10^1^
	P	1.734 × 10^−6^	3.182 × 10^−6^	3.182 × 10^−6^	1.734 × 10^−6^	1.734 × 10^−6^	1.734 × 10^−6^	2.127 × 10^−6^
	Rank	15	5	3	14	11	7	6
F5	Best	1.741 × 10^4^	2.512 × 10^3^	4.065 × 10^3^	3.076 × 10^4^	3.433 × 10^3^	2.896 × 10^3^	3.148 × 10^3^
	Mean	1.728 × 10^6^	4.513 × 10^4^	2.135 × 10^5^	2.620 × 10^5^	2.610 × 10^4^	3.459 × 10^4^	7.318 × 10^4^
	Std	5.822 × 10^6^	1.150 × 10^5^	8.232 × 10^4^	1.446 × 10^5^	3.718 × 10^4^	8.185 × 10^4^	9.923 × 10^4^
	P	1.734 × 10^−6^	1.020 × 10^−1^	1.921 × 10^−6^	1.734 × 10^−6^	8.730 × 10^−3^	2.712 × 10^−1^	5.792 × 10^−5^
	Rank	15	3	10	11	4	2	5
F6	Best	1.770 × 10^3^	1.722 × 10^3^	1.832 × 10^3^	1.655 × 10^3^	1.753 × 10^3^	1.676 × 10^3^	1.608 × 10^3^
	Mean	2.059 × 10^3^	1.834 × 10^3^	2.161 × 10^3^	1.857 × 10^3^	1.942 × 10^3^	1.872 × 10^3^	1.798 × 10^3^
	Std	1.762 × 10^2^	8.785 × 10^1^	1.720 × 10^2^	1.044 × 10^2^	1.170 × 10^2^	7.725 × 10^1^	1.043 × 10^2^
	P	3.515 × 10^−6^	1.108 × 10^−2^	1.734 × 10^−6^	6.639 × 10^−4^	8.466 × 10^−6^	6.892 × 10^−5^	6.884 × 10^−1^
	Rank	13	6	15	7	12	9	4
F7	Best	3.482 × 10^3^	2.724 × 10^3^	2.751 × 10^3^	3.174 × 10^3^	**2.593 × 10^3^**	3.034 × 10^3^	3.557 × 10^3^
	Mean	3.007 × 10^4^	1.047 × 10^4^	4.324 × 10^4^	3.783 × 10^4^	1.435 × 10^4^	8.844 × 10^3^	1.483 × 10^4^
	Std	3.576 × 10^4^	6.599 × 10^3^	4.877 × 10^4^	5.578 × 10^4^	1.221 × 10^4^	5.700 × 10^3^	1.219 × 10^4^
	P	2.843 × 10^−5^	1.108 × 10^−2^	1.287 × 10^−3^	3.317 × 10^−4^	1.484 × 10^−3^	1.714 × 10^−1^	2.585 × 10^−3^
	Rank	10	3	8	6	4	2	5
F8	Best	2.295 × 10^3^	2.308 × 10^3^	2.293 × 10^3^	2.293 × 10^3^	2.310 × 10^3^	2.306 × 10^3^	2.244 × 10^3^
	Mean	2.851 × 10^3^	2.422 × 10^3^	2.662 × 10^3^	2.557 × 10^3^	2.402 × 10^3^	2.330 × 10^3^	2.313 × 10^3^
	Std	2.956 × 10^2^	1.855 × 10^2^	7.115 × 10^2^	3.973 × 10^2^	7.435 × 10^1^	2.800 × 10^1^	**1.372 × 10^1^**
	P	1.921 × 10^−6^	1.734 × 10^−6^	3.405 × 10^−5^	1.921 × 10^−6^	1.734 × 10^−6^	2.353 × 10^−6^	1.734 × 10^−6^
	Rank	14	8	6	13	9	5	4
F9	Best	2.594 × 10^3^	2.529 × 10^3^	2.501 × 10^3^	2.526 × 10^3^	2.552 × 10^3^	2.509 × 10^3^	2.508 × 10^3^
	Mean	2.761 × 10^3^	2.764 × 10^3^	2.906 × 10^3^	2.810 × 10^3^	2.791 × 10^3^	2.751 × 10^3^	2.753 × 10^3^
	Std	8.523 × 10^1^	4.841 × 10^1^	1.058 × 10^2^	5.725 × 10^1^	5.010 × 10^1^	8.157 × 10^1^	6.672 × 10^1^
	P	3.389 × 10^−1^	7.271 × 10^−3^	1.973 × 10^−5^	3.182 × 10^−6^	3.724 × 10^−5^	7.731 × 10^−3^	3.501 × 10^−2^
	Rank	8	5	15	12	10	6	3
F10	Best	3.009 × 10^3^	2.906 × 10^3^	2.898 × 10^3^	2.923 × 10^3^	2.913 × 10^3^	2.912 × 10^3^	2.901 × 10^3^
	Mean	3.242 × 10^3^	2.948 × 10^3^	2.940 × 10^3^	3.011 × 10^3^	2.987 × 10^3^	2.963 × 10^3^	2.941 × 10^3^
	Std	1.793 × 10^2^	3.393 × 10^1^	2.467 × 10^1^	7.097 × 10^1^	5.214 × 10^1^	3.179 × 10^1^	2.931 × 10^1^
	P	1.734 × 10^−6^	1.566 × 10^−2^	3.854 × 10^−3^	2.879 × 10^−6^	2.879 × 10^−6^	1.477 × 10^−4^	5.710 × 10^−2^
	Rank	14	3	2	11	9	6	5
Mean Rank		12.8	4.8	8.1	10.3	8.5	5.3	4.7
+/=/−		9/1/0	8/2/0	9/0/1	10/0/0	10/0/0	7/3/0	7/3/0

**Table A18:** Comparison results with classic highly-cited algorithms on CEC2020 (Dim = 20).

Function	Index	EAO	PSO	DE	CMA-ES	WOA	SCA	MFO	HHO
F1	Best	2.258 × 10^5^	5.387 × 10^9^	9.873 × 10^7^	**5.890 × 10^2^**	9.940 × 10^7^	4.969 × 10^9^	6.293 × 10^3^	1.013 × 10^10^
	Mean	7.337 × 10^7^	9.866 × 10^9^	2.009 × 10^8^	8.245 × 10^9^	3.461 × 10^8^	8.337 × 10^9^	2.438 × 10^9^	2.346 × 10^10^
	Std	1.370 × 10^8^	2.595 × 10^9^	1.086 × 10^8^	7.579 × 10^9^	2.585 × 10^8^	1.899 × 10^9^	2.936 × 10^9^	7.815 × 10^9^
	P	1.734 × 10^−6^	1.057 × 10^−4^	1.484 × 10^−3^	1.891 × 10^−4^	1.734 × 10^−6^	6.339 × 10^−6^	1.734 × 10^−6^	1.734 × 10^−6^
	Rank	2	12	4	8	5	11	6	14
F2	Best	**2.068 × 10^3^**	4.129 × 10^3^	2.305 × 10^3^	5.224 × 10^3^	2.764 × 10^3^	4.680 × 10^3^	2.341 × 10^3^	3.599 × 10^3^
	Mean	3.006 × 10^3^	5.147 × 10^3^	**2.677 × 10^3^**	5.755 × 10^3^	4.241 × 10^3^	5.231 × 10^3^	3.447 × 10^3^	5.064 × 10^3^
	Std	5.007 × 10^2^	3.740 × 10^2^	**1.594 × 10^2^**	2.212 × 10^2^	7.572 × 10^2^	2.676 × 10^2^	5.677 × 10^2^	5.173 × 10^2^
	P	1.734 × 10^−6^	1.709 × 10^−3^	1.734 × 10^−6^	3.515 × 10^−6^	1.734 × 10^−6^	8.730 × 10^−3^	1.734 × 10^−6^	1.734 × 10^−6^
	Rank	2	12	1	15	7	13	3	11
F3	Best	7.595 × 10^2^	1.094 × 10^3^	8.295 × 10^2^	**7.348 × 10^2^**	8.800 × 10^2^	8.899 × 10^2^	7.686 × 10^2^	9.619 × 10^2^
	Mean	**8.028 × 10^2^**	1.279 × 10^3^	8.579 × 10^2^	8.182 × 10^2^	9.737 × 10^2^	9.383 × 10^2^	8.452 × 10^2^	1.034 × 10^3^
	Std	2.545 × 10^1^	9.953 × 10^1^	**1.386 × 10^1^**	1.673 × 10^1^	5.782 × 10^1^	1.984 × 10^1^	7.673 × 10^1^	3.772 × 10^1^
	P	1.734 × 10^−6^	1.734 × 10^−6^	8.730 × 10^−3^	1.734 × 10^−6^	1.734 × 10^−6^	1.852 × 10^−2^	1.734 × 10^−6^	1.734 × 10^−6^
	Rank	1	15	6	2	11	8	3	13
F4	Best	**1.905 × 10^3^**	2.475 × 10^3^	1.917 × 10^3^	4.980 × 10^3^	1.915 × 10^3^	2.047 × 10^3^	1.906 × 10^3^	2.166 × 10^4^
	Mean	**1.908 × 10^3^**	2.898 × 10^4^	2.063 × 10^3^	7.207 × 10^4^	2.108 × 10^3^	4.517 × 10^3^	1.400 × 10^4^	3.500 × 10^5^
	Std	**2.072 × 10^0^**	3.859 × 10^4^	5.857 × 10^2^	1.219 × 10^5^	2.905 × 10^2^	3.008 × 10^3^	1.949 × 10^4^	5.264 × 10^5^
	P	1.734 × 10^−6^	1.734 × 10^−6^	1.734 × 10^−6^	1.734 × 10^−6^	1.734 × 10^−6^	2.353 × 10^−6^	1.734 × 10^−6^	1.734 × 10^−6^
	Rank	1	12	3	13	6	8	9	14
F5	Best	**2.727 × 10^4^**	1.026 × 10^6^	7.761 × 10^5^	3.417 × 10^6^	1.259 × 10^5^	4.148 × 10^5^	5.891 × 10^4^	2.786 × 10^5^
	Mean	**2.520 × 10^5^**	3.966 × 10^6^	3.877 × 10^6^	1.310 × 10^7^	1.721 × 10^6^	2.300 × 10^6^	2.211 × 10^6^	5.106 × 10^6^
	Std	**2.728 × 10^5^**	3.240 × 10^6^	2.014 × 10^6^	6.059 × 10^6^	1.470 × 10^6^	1.119 × 10^6^	3.411 × 10^6^	4.369 × 10^6^
	P	1.734 × 10^−6^	1.734 × 10^−6^	1.734 × 10^−6^	1.025 × 10^−5^	1.734 × 10^−6^	1.639 × 10^−5^	1.734 × 10^−6^	1.734 × 10^−6^
	Rank	1	12	13	15	8	10	7	11
F6	Best	1.761 × 10^3^	1.952 × 10^3^	**1.629 × 10^3^**	2.421 × 10^3^	2.179 × 10^3^	2.115 × 10^3^	1.872 × 10^3^	2.638 × 10^3^
	Mean	2.216 × 10^3^	2.425 × 10^3^	**1.699 × 10^3^**	2.776 × 10^3^	2.799 × 10^3^	2.519 × 10^3^	2.154 × 10^3^	3.157 × 10^3^
	Std	2.171 × 10^2^	2.296 × 10^2^	**6.056 × 10^1^**	2.164 × 10^2^	2.775 × 10^2^	1.804 × 10^2^	2.005 × 10^2^	3.735 × 10^2^
	P	2.255 × 10^−3^	2.127 × 10^−6^	1.734 × 10^−6^	3.182 × 10^−6^	3.405 × 10^−5^	4.284 × 10^−1^	1.734 × 10^−6^	2.255 × 10^−3^
	Rank	3	6	1	11	12	9	2	15
F7	Best	7.469 × 10^3^	1.430 × 10^5^	5.178 × 10^5^	4.942 × 10^4^	2.412 × 10^4^	9.762 × 10^4^	2.295 × 10^4^	1.721 × 10^4^
	Mean	**7.613 × 10^4^**	7.234 × 10^5^	1.502 × 10^6^	5.035 × 10^6^	1.530 × 10^6^	6.843 × 10^5^	6.164 × 10^5^	1.411 × 10^6^
	Std	**6.393 × 10^4^**	5.841 × 10^5^	8.302 × 10^5^	6.113 × 10^6^	1.050 × 10^6^	5.367 × 10^5^	9.082 × 10^5^	1.916 × 10^6^
	P	1.921 × 10^−6^	1.734 × 10^−6^	1.734 × 10^−6^	2.353 × 10^−6^	2.603 × 10^−6^	1.494 × 10^−5^	2.603 × 10^−6^	1.921 × 10^−6^
	Rank	1	10	14	15	13	8	6	11
F8	Best	2.305 × 10^3^	2.970 × 10^3^	3.034 × 10^3^	**2.300 × 10^3^**	2.348 × 10^3^	2.835 × 10^3^	2.302 × 10^3^	3.587 × 10^3^
	Mean	2.427 × 10^3^	5.003 × 10^3^	3.908 × 10^3^	6.583 × 10^3^	5.111 × 10^3^	5.075 × 10^3^	3.904 × 10^3^	6.076 × 10^3^
	Std	6.359 × 10^2^	1.775 × 10^3^	9.535 × 10^2^	1.197 × 10^3^	1.799 × 10^3^	1.958 × 10^3^	1.374 × 10^3^	1.128 × 10^3^
	P	1.734 × 10^−6^	1.973 × 10^−5^	2.353 × 10^−6^	1.734 × 10^−6^	8.466 × 10^−6^	7.691 × 10^−6^	1.734 × 10^−6^	1.734 × 10^−6^
	Rank	1	12	7	15	9	10	6	14
F9	Best	**2.501 × 10^3^**	2.920 × 10^3^	2.905 × 10^3^	2.968 × 10^3^	2.978 × 10^3^	2.947 × 10^3^	2.844 × 10^3^	3.037 × 10^3^
	Mean	**2.882 × 10^3^**	3.017 × 10^3^	2.930 × 10^3^	3.008 × 10^3^	3.127 × 10^3^	3.009 × 10^3^	2.894 × 10^3^	3.271 × 10^3^
	Std	9.872 × 10^1^	7.513 × 10^1^	**1.229 × 10^1^**	2.152 × 10^1^	8.762 × 10^1^	2.056 × 10^1^	2.456 × 10^1^	1.766 × 10^2^
	P	1.360 × 10^−5^	9.842 × 10^−3^	5.752 × 10^−6^	2.353 × 10^−6^	5.216 × 10^−6^	4.165 × 10^−1^	1.734 × 10^−6^	1.360 × 10^−5^
	Rank	3	8	4	9	11	7	1	14
F10	Best	2.911 × 10^3^	3.185 × 10^3^	2.952 × 10^3^	2.959 × 10^3^	3.011 × 10^3^	3.122 × 10^3^	2.914 × 10^3^	3.674 × 10^3^
	Mean	**2.942 × 10^3^**	3.700 × 10^3^	3.021 × 10^3^	3.562 × 10^3^	3.075 × 10^3^	3.280 × 10^3^	3.048 × 10^3^	4.574 × 10^3^
	Std	3.133 × 10^1^	3.717 × 10^2^	3.596 × 10^1^	4.312 × 10^2^	4.806 × 10^1^	1.280 × 10^2^	1.579 × 10^2^	6.776 × 10^2^
	P	1.734 × 10^−6^	1.921 × 10^−6^	1.921 × 10^−6^	1.734 × 10^−6^	1.734 × 10^−6^	4.682 × 10^−3^	1.734 × 10^−6^	1.734 × 10^−6^
	Rank	1	13	5	11	7	9	4	15
Mean Rank		**1.6**	11.2	5.8	11.4	8.9	9.3	4.7	13.2
+/=/−		~	10/0/0	8/0/2	10/0/0	10/0/0	10/0/0	8/2/0	10/0/0

**Table A19:** Comparison results with newly proposed algorithms on CEC2020 (Dim = 20).

Function	Index	MShOA	GJO	WAA	SHO	HO	PO	AO
F1	Best	1.365 × 10^10^	1.814 × 10^9^	2.968 × 10^5^	8.831 × 10^9^	2.908 × 10^9^	1.899 × 10^8^	2.358 × 10^7^
	Mean	2.831 × 10^10^	5.553 × 10^9^	**5.326 × 10^5^**	1.457 × 10^10^	7.550 × 10^9^	2.077 × 10^9^	1.217 × 10^8^
	Std	8.264 × 10^9^	3.060 × 10^9^	**1.388 × 10^5^**	3.148 × 10^9^	2.521 × 10^9^	1.721 × 10^9^	1.214 × 10^8^
	P	1.734 × 10^−6^	1.734 × 10^−6^	3.515 × 10^−6^	1.734 × 10^−6^	1.734 × 10^−6^	1.734 × 10^−6^	2.183 × 10^−2^
	Rank	15	9	1	13	10	7	3
F2	Best	4.043 × 10^3^	2.697 × 10^3^	2.750 × 10^3^	3.471 × 10^3^	3.827 × 10^3^	2.340 × 10^3^	2.305 × 10^3^
	Mean	5.281 × 10^3^	4.296 × 10^3^	3.895 × 10^3^	4.589 × 10^3^	4.904 × 10^3^	3.866 × 10^3^	3.536 × 10^3^
	Std	4.790 × 10^2^	9.768 × 10^2^	5.174 × 10^2^	5.061 × 10^2^	5.262 × 10^2^	5.502 × 10^2^	5.679 × 10^2^
	P	1.734 × 10^−6^	1.973 × 10^−5^	8.466 × 10^−6^	1.734 × 10^−6^	1.734 × 10^−6^	1.639 × 10^−5^	2.255 × 10^−3^
	Rank	14	8	6	9	10	5	4
F3	Best	9.732 × 10^2^	8.001 × 10^2^	9.547 × 10^2^	8.790 × 10^2^	8.502 × 10^2^	8.271 × 10^2^	8.114 × 10^2^
	Mean	1.040 × 10^3^	8.560 × 10^2^	9.869 × 10^2^	9.414 × 10^2^	9.490 × 10^2^	9.203 × 10^2^	8.578 × 10^2^
	Std	3.555 × 10^1^	3.050 × 10^1^	2.887 × 10^1^	2.383 × 10^1^	3.280 × 10^1^	3.803 × 10^1^	3.056 × 10^1^
	P	1.734 × 10^−6^	3.515 × 10^−6^	1.734 × 10^−6^	1.734 × 10^−6^	1.734 × 10^−6^	1.734 × 10^−6^	6.339 × 10^−6^
	Rank	14	4	12	9	10	7	5
F4	Best	1.982 × 10^4^	1.919 × 10^3^	1.918 × 10^3^	4.413 × 10^3^	2.032 × 10^3^	1.918 × 10^3^	1.912 × 10^3^
	Mean	3.086 × 10^5^	4.412 × 10^3^	1.931 × 10^3^	1.421 × 10^4^	6.605 × 10^3^	1.941 × 10^3^	1.937 × 10^3^
	Std	2.343 × 10^5^	3.033 × 10^3^	7.272 × 10^0^	1.376 × 10^4^	3.149 × 10^3^	2.456 × 10^1^	2.632 × 10^1^
	P	1.734 × 10^−6^	1.734 × 10^−6^	1.734 × 10^−6^	1.734 × 10^−6^	1.734 × 10^−6^	1.734 × 10^−6^	1.734 × 10^−6^
	Rank	15	7	2	11	10	5	4
F5	Best	6.667 × 10^5^	3.902 × 10^4^	8.712 × 10^4^	4.447 × 10^5^	5.891 × 10^4^	7.091 × 10^4^	1.878 × 10^5^
	Mean	1.328 × 10^7^	8.229 × 10^5^	3.595 × 10^5^	1.170 × 10^6^	2.136 × 10^6^	8.676 × 10^5^	7.970 × 10^5^
	Std	1.202 × 10^7^	9.695 × 10^5^	3.415 × 10^5^	5.398 × 10^5^	1.120 × 10^6^	6.428 × 10^5^	4.813 × 10^5^
	P	1.734 × 10^−6^	3.854 × 10^−3^	7.521 × 10^−2^	1.734 × 10^−6^	3.182 × 10^−6^	2.613 × 10^−4^	5.307 × 10^−5^
	Rank	14	3	2	6	9	5	4
F6	Best	2.273 × 10^3^	1.819 × 10^3^	2.213 × 10^3^	2.137 × 10^3^	2.085 × 10^3^	2.006 × 10^3^	1.910 × 10^3^
	Mean	2.931 × 10^3^	2.217 × 10^3^	2.816 × 10^3^	2.487 × 10^3^	2.522 × 10^3^	2.451 × 10^3^	2.199 × 10^3^
	Std	3.148 × 10^2^	1.902 × 10^2^	2.136 × 10^2^	1.998 × 10^2^	2.590 × 10^2^	2.547 × 10^2^	2.006 × 10^2^
	P	2.353 × 10^−6^	8.774 × 10^−1^	2.127 × 10^−6^	2.225 × 10^−4^	3.112 × 10^−5^	2.255 × 10^−3^	8.451 × 10^−1^
	Rank	14	5	13	8	10	7	4
F7	Best	1.023 × 10^4^	**7.140 × 10^3^**	8.859 × 10^3^	6.881 × 10^4^	5.293 × 10^4^	7.707 × 10^3^	6.950 × 10^4^
	Mean	2.542 × 10^6^	5.728 × 10^5^	3.284 × 10^5^	2.998 × 10^5^	6.305 × 10^5^	2.082 × 10^5^	8.036 × 10^5^
	Std	2.899 × 10^6^	1.059 × 10^6^	3.122 × 10^5^	3.459 × 10^5^	6.201 × 10^5^	2.241 × 10^5^	7.362 × 10^5^
	P	2.353 × 10^−6^	1.891 × 10^−4^	4.860 × 10^−5^	6.984 × 10^−6^	1.734 × 10^−6^	9.627 × 10^−4^	3.515 × 10^−6^
	Rank	12	4	5	3	7	2	9
F8	Best	3.039 × 10^3^	2.430 × 10^3^	2.308 × 10^3^	3.087 × 10^3^	2.597 × 10^3^	2.342 × 10^3^	2.316 × 10^3^
	Mean	5.455 × 10^3^	3.235 × 10^3^	5.516 × 10^3^	4.175 × 10^3^	3.340 × 10^3^	2.483 × 10^3^	**2.333 × 10^3^**
	Std	1.043 × 10^3^	9.054 × 10^2^	1.169 × 10^3^	9.817 × 10^2^	9.508 × 10^2^	1.744 × 10^2^	**1.527 × 10^1^**
	P	1.921 × 10^−6^	2.597 × 10^−5^	2.127 × 10^−6^	1.734 × 10^−6^	2.163 × 10^−5^	3.112 × 10^−5^	3.112 × 10^−5^
	Rank	13	4	11	8	5	3	2
F9	Best	3.007 × 10^3^	2.845 × 10^3^	3.112 × 10^3^	3.031 × 10^3^	2.983 × 10^3^	2.869 × 10^3^	2.842 × 10^3^
	Mean	3.190 × 10^3^	2.944 × 10^3^	3.354 × 10^3^	3.118 × 10^3^	3.101 × 10^3^	2.963 × 10^3^	2.896 × 10^3^
	Std	1.344 × 10^2^	5.117 × 10^1^	1.617 × 10^2^	5.082 × 10^1^	7.098 × 10^1^	4.683 × 10^1^	3.057 × 10^1^
	P	1.734 × 10^−6^	8.217 × 10^−3^	1.734 × 10^−6^	1.734 × 10^−6^	1.921 × 10^−6^	4.897 × 10^−4^	4.165 × 10^−1^
	Rank	13	5	15	12	10	6	2
F10	Best	3.422 × 10^3^	3.020 × 10^3^	**2.900 × 10^3^**	3.140 × 10^3^	3.095 × 10^3^	2.927 × 10^3^	2.930 × 10^3^
	Mean	4.704 × 10^3^	3.115 × 10^3^	2.977 × 10^3^	3.393 × 10^3^	3.311 × 10^3^	3.053 × 10^3^	3.005 × 10^3^
	Std	1.037 × 10^3^	1.021 × 10^2^	**2.621 × 10^1^**	2.197 × 10^2^	1.745 × 10^2^	5.459 × 10^1^	4.212 × 10^1^
	P	1.734 × 10^−6^	1.734 × 10^−6^	4.534 × 10^−4^	1.734 × 10^−6^	1.734 × 10^−6^	1.921 × 10^−6^	1.494 × 10^−5^
	Rank	14	8	2	12	10	6	3
Mean Rank		13.8	5.7	6.9	9.1	9.1	5.3	4
+/=/−		10/0/0	9/1/0	9/0/1	10/0/0	10/0/0	10/0/0	9/0/1

## References

[1] Jones M., Djahel S., Welsh K. (2023). Path-planning for unmanned aerial vehicles with environment complexity considerations: A survey. ACM Comput. Surv..

[2] Meng W., Zhang X., Zhou L., Guo H., Hu X. (2025). Advances in UAV Path Planning: A Comprehensive Review of Methods, Challenges, and Future Directions. Drones.

[3] Aljalaud F., Kurdi H., Youcef-Toumi K. (2023). Bio-inspired multi-UAV path planning heuristics: A review. Mathematics.

[4] Wayahdi M.R., Ginting S.H.N., Syahputra D. (2021). Greedy, A-Star, and Dijkstra’s algorithms in finding shortest path. Int. J. Adv. Data Inf. Syst..

[5] Hart P.E., Nilsson N.J., Raphael B. (1968). A formal basis for the heuristic determination of minimum cost paths. IEEE Trans. Syst. Sci. Cybern..

[6] Koenig S., Likhachev M. (2002). D* lite. Proceedings of the Eighteenth National Conference on Artificial Intelligence.

[7] Ma C.S., Miller R.H. (2006). MILP optimal path planning for real-time applications. Proceedings of the 2006 American Control Conference, Minneapolis, MN, USA, 14–16 June 2006.

[8] Mac T.T., Copot C., Tran D.T., De Keyser R. (2016). Heuristic approaches in robot path planning: A survey. Robot. Auton. Syst..

[9] Kennedy J., Eberhart R. (1995). Particle swarm optimization. Proceedings of ICNN’95-International Conference on Neural Networks.

[10] Storn R., Price K. (1997). Differential evolution–a simple and efficient heuristic for global optimization over continuous spaces. J. Glob. Optim..

[11] Mirjalili S., Lewis A. (2016). The whale optimization algorithm. Adv. Eng. Softw..

[12] Mirjalili S. (2015). Moth-flame optimization algorithm: A novel nature-inspired heuristic paradigm. Knowl.-Based Syst..

[13] Chopra N., Ansari M.M. (2022). Golden jackal optimization: A novel nature-inspired optimizer for engineering applications. Expert Syst. Appl..

[14] Xue J., Shen B. (2023). Dung beetle optimizer: A new meta-heuristic algorithm for global optimization. J. Supercomput..

[15] Fu Y., Liu D., Chen J., He L. (2024). Secretary bird optimization algorithm: A new metaheuristic for solving global optimization problems. Artif. Intell. Rev..

[16] Qi Y., Jiang H., Huang G., Yang L., Wang F., Xu Y. (2025). Multi-UAV path planning considering multiple energy consumptions via an improved bee foraging learning particle swarm optimization algorithm. Sci. Rep..

[17] Zeng H., Tong L., Xia X. (2024). Multi-UAV cooperative coverage search for various regions based on differential evolution algorithm. Biomimetics.

[18] Yu B., Fan S., Cui W., Xia K., Wang L. (2024). A Multi-UAV cooperative mission planning method based on SA-WOA algorithm for three-dimensional space atmospheric environment detection. Robotica.

[19] Karthik K., Balasubramanian C., Praveen R. (2025). Hybrid golden Jackal and moth flame optimization algorithm based coverage path planning in heterogeneous UAV networks. Sci. Rep..

[20] Lou T., Wang Y., Yue Z., Zhao L. (2024). Multi-UAV collaborative trajectory planning for 3D terrain based on CS-GJO algorithm. Complex Syst. Model. Simul..

[21] Yang L., Zhang X., Li Z., Li L., Shi Y. (2025). A LODBO algorithm for multi-UAV search and rescue path planning in disaster areas. Chin. J. Aeronaut..

[22] Zheng X., Liu R., Liu X. (2025). Simulation Application of Adaptive Strategy Hybrid Secretary Bird Optimization Algorithm in Multi-UAV 3D Path Planning. Computers.

[23] Abualigah L., Yousri D., Abd Elaziz M., Ewees A.A., Al-Qaness M.A., Gandomi A.H. (2021). Aquila optimizer: A novel meta-heuristic optimization algorithm. Comput. Ind. Eng..

[24] Taleb S.M., Yasin E.T., Ait Saadi A., Dogan M., Yahia S., Meraihi Y., Koklu M., Mirjalili S., Ramdane-Cherif A. (2025). A comprehensive survey of aquila optimizer: Theory, variants, hybridization, and applications. Arch. Comput. Methods Eng..

[25] Zeng Q., Zhou Y., Zhou G., Luo Q. (2025). Multi-strategies enhanced aquila optimizer for global optimization: Comprehensive review and comparative analysis. J. Comput. Des. Eng..

[26] Al-Majidi S.D., Alturfi A.M., Al-Nussairi M.K., Hussein R.A., Salgotra R., Abbod M.F. (2025). A robust automatic generation control system based on hybrid Aquila Optimizer-Sine Cosine Algorithm. Results Eng..

[27] Bai L., Pei Z., Wang J., Zhou Y. (2025). Multi-Strategy Improved Aquila Optimizer Algorithm and Its Application in Railway Freight Volume Prediction. Electronics.

[28] Wang X., Li R., Luo X., Guan X. (2025). Improved differential mutation aquila optimizer-based optimization dispatching strategy for hybrid energy ship power system. IEEE Trans. Transp. Electrif..

[29] Singla M.K., Muhammed Ali S.A., Kumar R., Jangir P., Khishe M., Gulothungan G., Mahmoud H.A. (2025). Revolutionizing proton exchange membrane fuel cell modeling through hybrid aquila optimizer and arithmetic algorithm optimization. Sci. Rep..

[30] Zhang J., Gao Z., Li S., Zhao J. (2025). An improved aquila optimizer with local escaping operator and its application in uav path planning. Recent Adv. Comput. Sci. Commun..

[31] Liu X., Wang L., Ma Y., Shao P. (2025). Collaborative Trajectory Planning for Stereoscopic Agricultural Multi-UAVs Driven by the Aquila Optimizer. Comput. Mater. Contin..

[32] Tang X., Jia C., Qu P. (2025). EAPO: A Multi-Strategy-Enhanced Artificial Protozoa Optimizer and Its Application to 3D UAV Path Planning. Mathematics.

[33] Xu Z., Wang Z., Liu R., Huang C., Shi Y., Wang M., Chen H. (2025). Efficient multi-UAV path planning in dynamic and complex environments using hybrid polar lights optimization. J. King Saud Univ. Comput. Inf. Sci..

[34] Liu X., Wang F., Liu Y., Li L. (2025). A Multi-Objective Black-Winged Kite Algorithm for Multi-UAV Cooperative Path Planning. Drones.

[35] Zhang X., Yu G., Jin Y., Qian F. (2023). Elitism-based transfer learning and diversity maintenance for dynamic multi-objective optimization. Inf. Sci..

[36] Yang Q., Yan J.Q., Gao X.D., Xu D.D., Lu Z.Y., Zhang J. (2022). Random neighbor elite guided differential evolution for global numerical optimization. Inf. Sci..

[37] Hansen N., Müller S.D., Koumoutsakos P. (2003). Reducing the time complexity of the derandomized evolution strategy with covariance matrix adaptation (CMA-ES). Evol. Comput..

[38] Mirjalili S. (2016). SCA: A sine cosine algorithm for solving optimization problems. Knowl.-Based Syst..

[39] Heidari A.A., Mirjalili S., Faris H., Aljarah I., Mafarja M., Chen H. (2019). Harris hawks optimization: Algorithm and applications. Future Gener. Comput. Syst..

[40] Sánchez Cortez J.A., Peraza Vázquez H., Peña Delgado A.F. (2025). A Novel Bio-Inspired Optimization Algorithm Based on Mantis Shrimp Survival Tactics. Mathematics.

[41] Cheng J., De Waele W. (2024). Weighted average algorithm: A novel meta-heuristic optimization algorithm based on the weighted average position concept. Knowl.-Based Syst..

[42] Zhao S., Zhang T., Ma S., Wang M. (2023). Sea-horse optimizer: A novel nature-inspired meta-heuristic for global optimization problems. Appl. Intell..

[43] Amiri M.H., Mehrabi Hashjin N., Montazeri M., Mirjalili S., Khodadadi N. (2024). Hippopotamus optimization algorithm: A novel nature-inspired optimization algorithm. Sci. Rep..

[44] Lian J., Hui G., Ma L., Zhu T., Wu X., Heidari A.A., Chen Y., Chen H. (2024). Parrot optimizer: Algorithm and applications to medical problems. Comput. Biol. Med..

[45] Olorunda O., Engelbrecht A.P. (2008). Measuring exploration/exploitation in particle swarms using swarm diversity. Proceedings of the 2008 IEEE Congress on Evolutionary Computation (IEEE World Congress on Computational Intelligence), Hong Kong, China, 1–6 June 2008.

[46] Črepinšek M., Liu S.H., Mernik M. (2013). Exploration and exploitation in evolutionary algorithms: A survey. ACM Comput. Surv. (CSUR).

